# Stereoselective Alkylation of Chiral Titanium(IV) Enolates with *tert*-Butyl Peresters

**DOI:** 10.1021/acs.orglett.1c03366

**Published:** 2021-10-26

**Authors:** Marina Pérez-Palau, Nil Sanosa, Pedro Romea, Fèlix Urpí, Rosa López, Enrique Gómez-Bengoa, Mercè Font-Bardia

**Affiliations:** ‡ Secció de Química Orgànica, Departament de Química Inorgànica i Orgànica and Institut de Biomedicina de la Universitat de Barcelona (IBUB), Universitat de Barcelona, Carrer Martí i Franqués 1-11, 08028 Barcelona, Catalonia, Spain; & Departamento de Química Orgánica I, Universidad del Pais Vasco, UPV/EHU, Apdo. 1072, 20080 San Sebastián, Spain; # Unitat de Difracció de RX. CCiTUB. Universitat de Barcelona. Carrer Solé i Sabarís 1-3, 08028 Barcelona, Catalonia, Spain

## Abstract

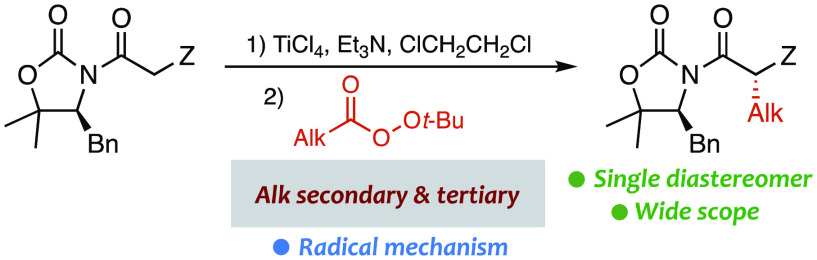

Here, we present a new stereoselective alkylation of titanium(IV) enolates of chiral *N*-acyl oxazolidinones with *tert*-butyl peresters from Cα-branched aliphatic carboxylic acids, which proceeds through the decarboxylation of the peresters and the subsequent formation of alkyl radicals to produce the alkylated adducts with an excellent diastereoselectivity. Theoretical calculations account for the observed reactivity and the outstanding stereocontrol. Importantly, the resultant compounds can be easily converted into ligands for asymmetric and catalytic transformations.

The need for more efficient and broad scope methods for the stereoselective construction of chiral molecular architectures is an endless source of inspiration for the development of new carbon–carbon bond-forming reactions.^1^ In this context and despite the advances reported in the last decades, the α-alkylation of carbonyl compounds still remains as a challenging objective.^2^ It is certainly true that successful methods based on the alkylation of metal enolates and enamines are widespread, but they are usually restricted to a privileged set of alkylating agents, namely sterically unhindered and active alkyl halides or sulfonates, able to react through an S_N_2-like mechanism.^3−5^ Alternative methods based on an S_N_1-like mechanism have been also reported, but they mostly require stabilized carbenium or oxocarbenium intermediates.^6−8^ As a result, the chemo- and stereoselective introduction of any secondary or tertiary alkyl groups continues to be an unresolved issue.^9^

Radical chemistry may offer an appealing way to achieve such an objective. Indeed, the tremendous success of the SOMO activation mode concept coined by MacMillan in the context of the direct and asymmetric alkylation of aldehydes illustrates the synthetic potential of the radical approach.^10^ Inspired by these ideas and considering the biradical character of the titanium(IV) enolates,^11^ we envisaged that they might undergo highly stereoselective alkylations provided that the required radical intermediates were generated in the reaction mixture. The feasibility of such an approach was clearly demonstrated in the alkylation of chiral *N*-acyl oxazolidinones with diacyl peroxides (Scheme 1).^12−14^ Unfortunately, diacyl peroxides from α-branched aliphatic carboxylic acids are difficult to manipulate, which made the reaction with tertiary alkyl groups particularly elusive. In the search for more stable carboxylic acid derivatives to enable the introduction of secondary and tertiary alkyl groups we focused our attention on redox-active esters (Scheme 1).^15^ Widely used phthalimide-derived esters^16^ containing an O–N bond proved to be unreactive, but peresters containing an O–O bond turned out to be much more satisfactory.^17^ Herein, we describe the chemo- and stereoselective Cα alkylation of titanium(IV) enolates from chiral *N*-acyl oxazolidinones with *tert*-butyl peresters from branched aliphatic carboxylic acids, which permits the stereocontrolled introduction of secondary and tertiary alkyl groups with moderate to high yields (Scheme 1). Importantly, this method gives a straightforward access to enantiomerically pure intermediates that can be employed as precursors for ligands in catalytic and asymmetric synthesis.^18^

**Scheme 1:**
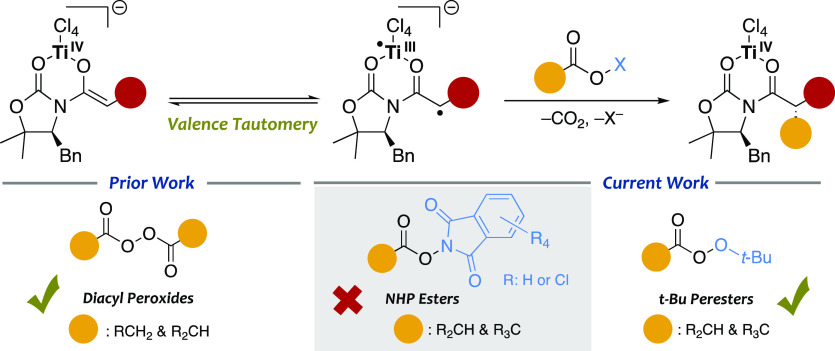
Stereoselective Decarboxylative Alkylation of Titanium(IV) Enolates from Chiral *N*-Acyloxazolidinones

Taking advantage of our experience, we were pleased to observe that the titanium(IV) enolate of (*S*) 4-benzyl-5,5-dimethyl-*N*-propanoyl-1,3-oxazolidin-2-one (**1** in Table 1) reacted with the *tert*-butyl perester from 1-adamantanecarboxylic acid (**a** in Table 1) under mild conditions similar to those employed for the alkylation with diacyl peroxides.^12^ Indeed, the alkylated adduct **1a** was isolated with a high yield and an excellent diastereoselectivity (74% and dr 97:3, see Table 1) through the simple stirring of a mixture of the titanium(IV) enolate of **1** with 1.5 equiv of **a** in 1,2-dichloroethane for 1.5 h at room temperature. Slight variations of such conditions also gave the desired adduct **1a** but in lower yields (Table 1).

**Table 1:**
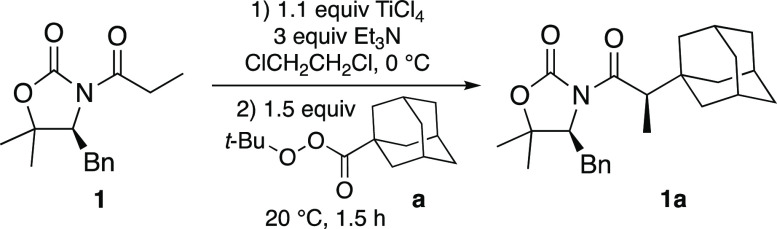
Examination of the Alkylation Conditions

entry	changes on the reaction conditions	yield^a^ (%)
1	none	74
2	*i*-Pr_2_NEt	66
3	2 equiv of **a**	62
4	0 °C for 2 h	58
5	2 equiv of **a** at 0 °C for 2 h	60

a Isolated yield after chromatographic purification of **1a**

The experimental procedure was next applied to a number of *tert*-butyl peresters from Cα branched carboxylic acids.^19^ The introduction of tertiary alkyl groups proved to be possible in variable yields and heavily dependent on their structure but with outstanding stereocontrol since a single diastereomer (dr ≥97:3) of the alkylated adducts **1a**–**d** was observed in all cases. Indeed, the results summarized in Scheme 2 show that they range from excellent for **1a** (74%) to low for **1d** in which perester **d** contains a *tert*-butyl-like chain possessing an aryl ether (23%). Importantly, peresters **b**–**d** react slowly compared to **a**, and we have occasionally observed the formation of the carboxylic acid derived from the reaction of the titanium enolate from **1** with the carbon dioxide released in the perester decarboxylation. Therefore, slow kinetics allow undesired side reactions to emerge and reduce the overall yield. Remarkably, the stereochemical outcome of the alkylation was firmly established through X-ray analysis of *tert*-butyl alkylated adduct **1c**.

**Scheme 2:**
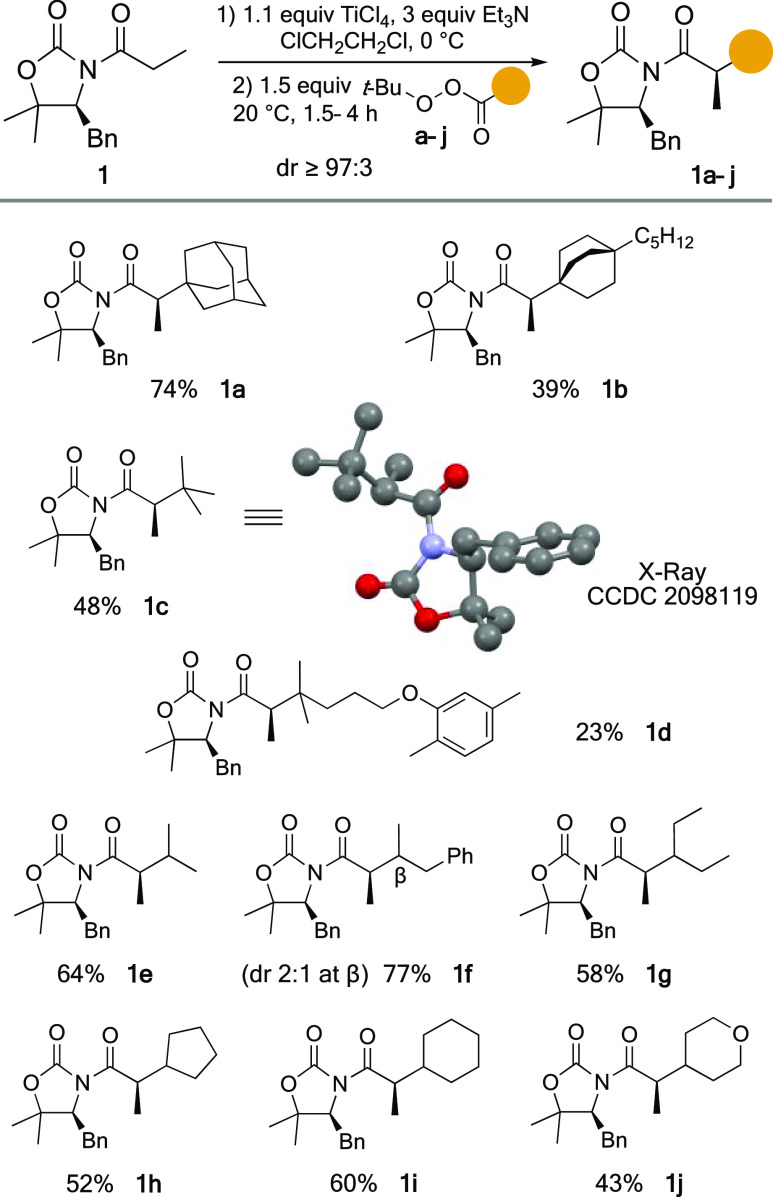
Alkylation of **1** with *tert*-Butyl Peresters **a**–**j** from Cα-Branched Carboxylic Acids

The reaction with secondary alkyl groups proved to be much more successful. As summarized in Scheme 2, the reaction with *tert*-butyl peresters **e**–**j** with Cα acyclic and cyclic aliphatic chains also gave the corresponding adducts **1e**–**j** as a single diastereomer (dr ≥97:3) in good to high yields. Finally, it is worth pointing out the lack of stereocontrol of the β-stereocenter in the alkylation with perester **f**, so adduct **1f** was isolated as a 2:1 mixture of two diastereomers (Scheme 2).

Having established the scope of the alkylating agent, we next examined the influence of the *N*-acyl group on the outcome of the alkylation with **a**. The reaction turned out to be sensitive to steric hindrance but at the same time chemoselective. Indeed, a variety of functional groups as double or triple bonds, esters, or phenyl rings may be embedded in the acyl chain and produce the corresponding alkylated adducts in yields up to 67% (Scheme 3). Importantly, protected α-hydroxy and α-amino acyl derivatives (α-OTBS and α-pyrrole, **8** and **9**, respectively, in Scheme 3) proved to be successful platforms from which the alkylated adducts **8a** and **9a** were obtained in high yields in a multigram scale, which demonstrates the robustness of the method and represents a straightforward way to get access to enantiomerically pure α-hydroxy and α-amino acids.

**Scheme 3:**
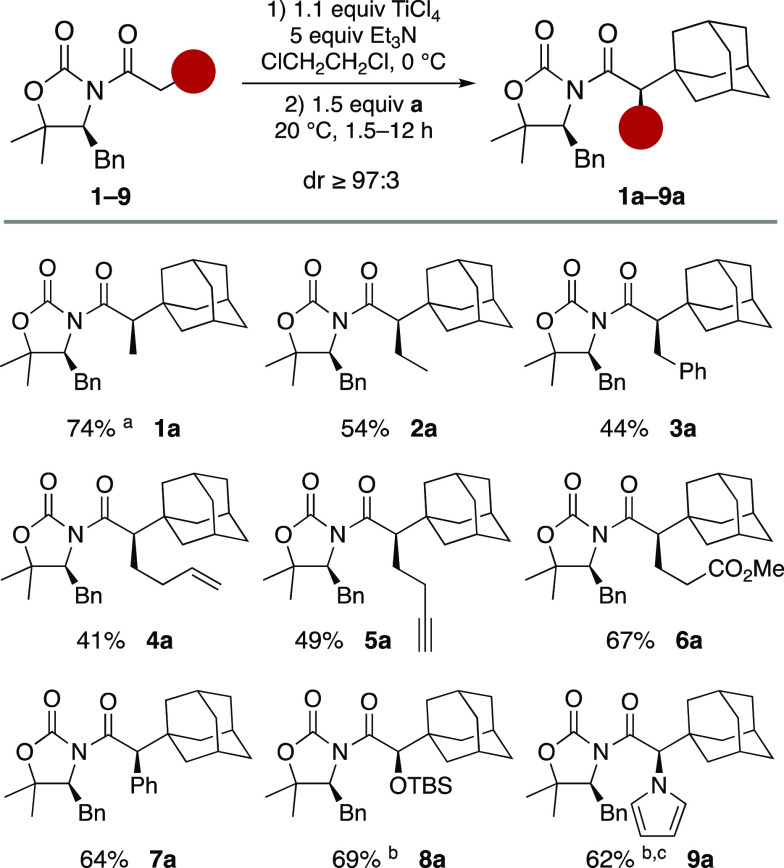
Alkylation of **1**–**9** with *tert*-Butyl Perester of 1-Adamantanecarboxylic Acid (**a**) 3 equiv of Et_3_N was employed. Yield at 6.5 mmol scale. The enolization was carried out at −20 °C.

At this point, we carried out a comprehensive theoretical study to unveil the origin of the observed reactivity and selectivity. As for the reaction with diacyl peroxides,^12^ DFT calculations^20^ of the alkylation of **1** with perester **a** indicated that it also may proceed through an electron transfer from **I** (Scheme 4), the biradical form of titanium enolates,^11^ to the σ* of the O–O bond of **a**. Thus, a single-electron transfer (SET) redox reaction causes the formation of the Ti(IV) radical **II** by a one electron loss and triggers the cleavage of the O–O bond, which produces an oxygen radical and an oxygen anion species. Due to the lack of symmetry of the perester, such a fragmentation may produce up to four different species shown in Scheme 4. These species may be in an equilibrium favoring the carboxylate anion **III**, more stable than the radical counterpart **IV**. However, **IV** is a very unstable intermediate and undergoes a spontaneous decarboxylation to the corresponding tertiary radical **V** in an almost barrierless step (ΔΔ*G*^⧧^ < 5 kcal mol^–1^); importantly, a parallel decomposition of anion **III** is precluded by kinetic and thermodynamic reasons (Δ*G*° ≈ 50 kcal mol^–1^). Thus, a Curtin–Hammett model may account for the formation of the adamantly radical **V**, which combines with the highly reactive Ti(IV) radical **II** to lead to the alkylated product **1a** after C–C bond formation and decoordination of the titanium.

**Scheme 4:**
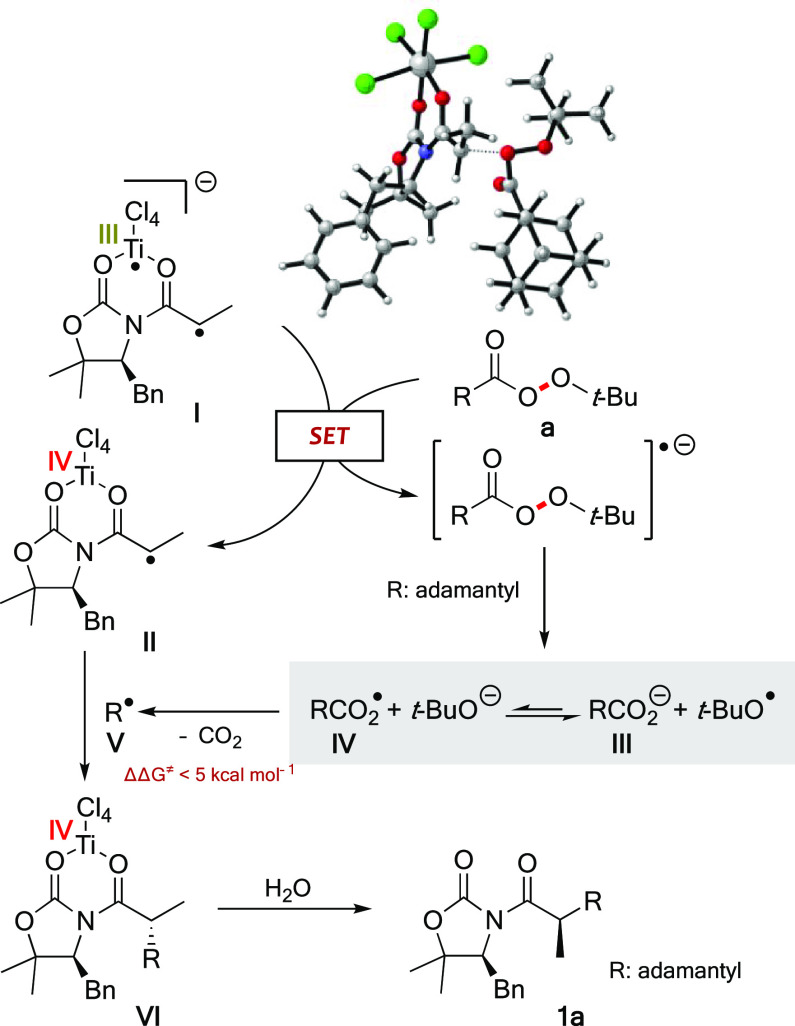
Mechanistic Proposal

Given the short distance at which the reagents must approach for the occurrence of the electron transfer, and due to the bulkiness of **I** and **a**, a good diastereoselectivity was ensured. Thereby, a remarkable minimum energy difference of at least 5.0 kcal·mol^–1^ corresponding to a dr >99:1 was calculated for the approach of distinct conformations of the perester to both π-faces of the enolate, with C–O distances ranging from 1.8 to 4 Å. This effect can be visualized in the 3D-representation shown in Scheme 4 of the approach between **I** and **a**, where the bulky adamantyl perester and the benzyl directing group are located at opposite faces of the enolate. Therefore, such a proposal accounts for both the observed reactivity and stereoselectivity since carbon-centered alkyl radicals are involved in the alkylation, whose stereochemical outcome hinges on the approach of the entire perester to the less sterically shielded *Si* π-face of the enolate.

Eventually, the easy access to α-adamantyl alkylated adducts **1a**–**9a** led us to explore their conversion into enantiomerically pure building blocks and derivatives that might be employed as ligands for chiral catalysts. The results matched our expectations (Scheme 5). Indeed, reductive removal of the chiral auxiliary from **1a** with LiBH_4_ gave the corresponding alcohol **10** in 64% yield. Furthermore, 1,2-dihydroxy and 2-amino-1-hydroxy derivatives **11** and **12**, respectively, were synthesized from adducts **8a** and **9a** in a similar way, which represents a straightforward approach to such interesting ligands.^21^

**Scheme 5:**
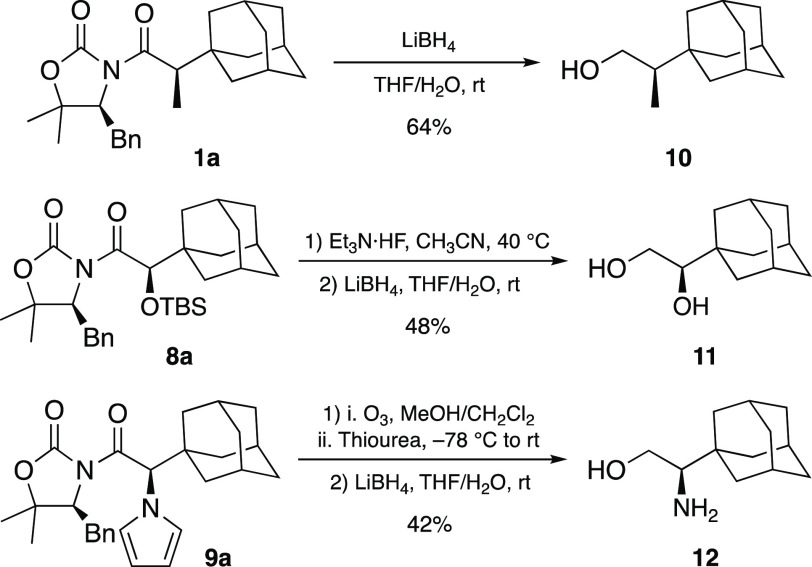
Synthesis of Enantiomerically Pure Derivatives

In summary, we have developed a highly stereoselective alkylation of titanium(IV) enolates of a variety of chiral *N*-acyl oxazolidinones with *tert*-butyl peresters from Cα branched aliphatic acids under experimentally mild conditions. The resultant alkylated adducts are isolated in moderate to high yields as a single diastereomer (dr ≥97:3), which represents an appealing entry to the challenging alkylation of metal enolates with secondary or tertiary alkyl groups. Computational studies have revealed that the success of such an approach is based on the reduction of the *tert*-butyl perester by the enolate, which triggers a radical-like transformation. Finally, it should be noted that this method complements a parallel and previously reported introduction of secondary and primary alkyl groups based on the use of diacyl peroxides. All together, both pieces of reactivity permit the diastereoselective Cα alkylation of titanium enolates with a broad range of alkyl groups (Scheme 6).

**Scheme 6:**
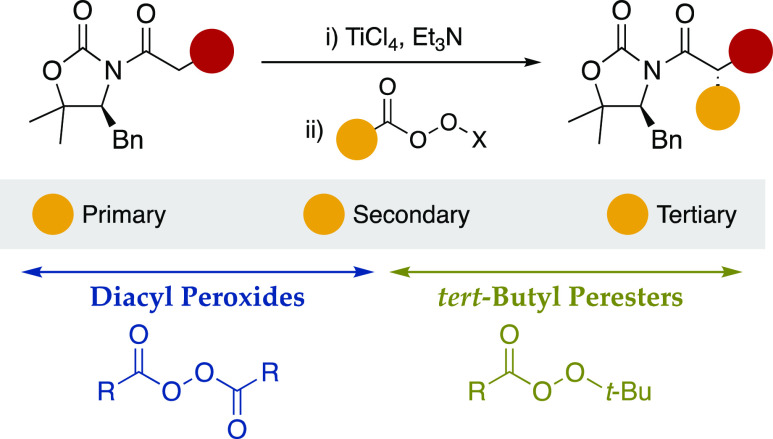
Cα Alkylation

## Supporting Information Available

Experimental details, compound characterization, and copies of ^1^H and ^13^C NMR spectra (PDF)
Crystallographic data for **1c** (PDF)

## Accession Codes

CCDC 2098119 contains the supplementary crystallographic data for this paper. These data can be obtained free of charge via www.ccdc.cam.ac.uk/data_request/cif, or by emailing data_request@ccdc.cam.ac.uk, or by contacting The Cambridge Crystallographic Data Centre, 12 Union Road, Cambridge CB2 1EZ, UK; fax: +44 1223 336033.
