# Supplementary material for: Stereoselective Alkylation of Chiral Titanium(IV) Enolates with tert-Butyl Peresters
Source: Org Lett. 2021 Oct 26;23(22):8852–6. doi: 10.1021/acs.orglett.1c03366 (PMC8609576; doi:10.1021/acs.orglett.1c03366)
Supplement: Supplementary file 1 — ol1c03366_si_001.pdf [file ol1c03366_si_001.pdf]

Supporting Information

-

Experimental Procedures and Spectra

Stereoselective Alkylation of Chiral Titanium(IV) Enolates with *tert*-Butyl Peresters

Marina Pérez-Palau,[†] Nil Sanosa,[†,¶] Pedro Romea,[*,†] Fèlix Urpí,[*,†] Rosa López,[&] Enrique Gómez-Bengoa,[*,&] and Mercè Font-Bardia[#]

† Secció de Química Orgànica, Departament de Química Inorgànica i Orgànica and Institut de Biomedicina de la Universitat de Barcelona (IBUB), Universitat de Barcelona, Carrer Martí i Franqués 1-11, 08028 Barcelona, Catalonia, Spain

& Departamento de Química Orgánica I, Universidad del Pais Vasco, UPV/EHU, Apdo. 1072, 20080 San Sebastián, Spain

# Unitat de Difracció de RX. CCiTUB. Universitat de Barcelona. Carrer Solé i Sabarís 1-3, 08028 Barcelona, Catalonia, Spain

Table of Contents

1 General Information 2

2 Preparation of Starting Materials 3

2.1 Synthesis of *N*-Acyl Oxazolidinones 3

2.2 Synthesis of *tert*-Butyl Peresters 4

3 Optimization Tables 9

3.1 Screening of the Reaction Conditions with **1a** as Model Substrate 9

3.2 Attempts to Minimize By-Product **SI2** 9

4 Scope of the Alkylation Reaction 11

4.1 Alkylation of **1** with *tert*-Butyl Peresters **a**–**j** (Scheme 2) 11

4.2 Alkylation of **1**–**9** with *tert*-Butyl 1-Adamantane Carboperoxoate **a** (Scheme 3) 17

5 Synthesis of Enantiomerically Pure Derivatives (Scheme 5) 22

6 X-Ray Analysis 25

7 ^{1}H NMR and ^{13}C NMR Spectra 27

8 References 101

1 General Information

Unless otherwise noted, reactions were conducted in oven-dried glassware under inert atmosphere of N_2 with anhydrous solvents. The solvents and reagents were dried and purified when necessary according to standard procedures. Commercially available reagents were used as received.

Hot plates with aluminium blocks were used to carry out reactions that required heating.

Analytical thin-layer chromatography (TLC) was carried out on Merck silica gel 60 F_{254} plates and analyzed by UV (254 nm) and stained with phosphomolybdic acid or $KMnO_4$; column chromatographies were carried under low pressure (flash) conditions and performed on SDS silica gel 60 (35–70 μm). Eluents are indicated in brackets in each case. R_f values are approximate.

Melting points (mp) were determined with a Stuart SMP10 apparatus and are uncorrected.

Specific rotations ($[\alpha]_D^{20}$) were determined at 20 °C on a Perkin-Elmer 241 MC polarimeter equipped with a sodium lamp (λ 589 nm, D-line). Concentration (g/dL) and solvent used are indicated in brackets.

IR spectra (Attenuated Total Reflectance, ATR) were recorded on a Nicolet 6700 FT-IR Thermo Scientific spectrometer and only the more representative frequencies (ν) are reported in cm^{-1}.

^1H NMR (400 MHz) and ^{13}C NMR (100.6 MHz) spectra were recorded at room temperature on a Varian Mercury 400 or a Bruker 400 Avance III. Chemical shifts (δ) are quoted in ppm and referenced to internal TMS (δ 0.00 for ^1H NMR) and $CDCl_3$ (δ 77.0 for ^{13}C NMR). Data are reported as follows: chemical shift (number of protons, multiplicity, coupling constant(s)); multiplicity is reported as follows: s, singlet; d, doublet; t, triplet; q, quartet; quint, quintet; sext, sextet; hept, heptuplet; oct, octet; m, multiplet; br, broad signal (and their corresponding combinations); coupling constants (*J*) are quoted in Hz. Where necessary, 2D techniques (COSY, HSQC) were also used to assist on structure elucidation.

High resolution mass spectra (HRMS) were obtained with an Agilent 1100 spectrometer with a TOF analyzer by the Unitat d'Espectrometria de Masses, Universitat de Barcelona.

2 Preparation of Starting Materials

2.1 Synthesis of *N*-Acyl Oxazolidinones

Chiral auxiliary, (*S*)-4-benzyl-5,5-dimethyl-1,3-oxazolidin-2-one (**SI1**), was prepared according to the procedure described by Davies.[1] *N*-Acyl oxazolidinones **1–6** and **8–9** were prepared according to the literature.[2]

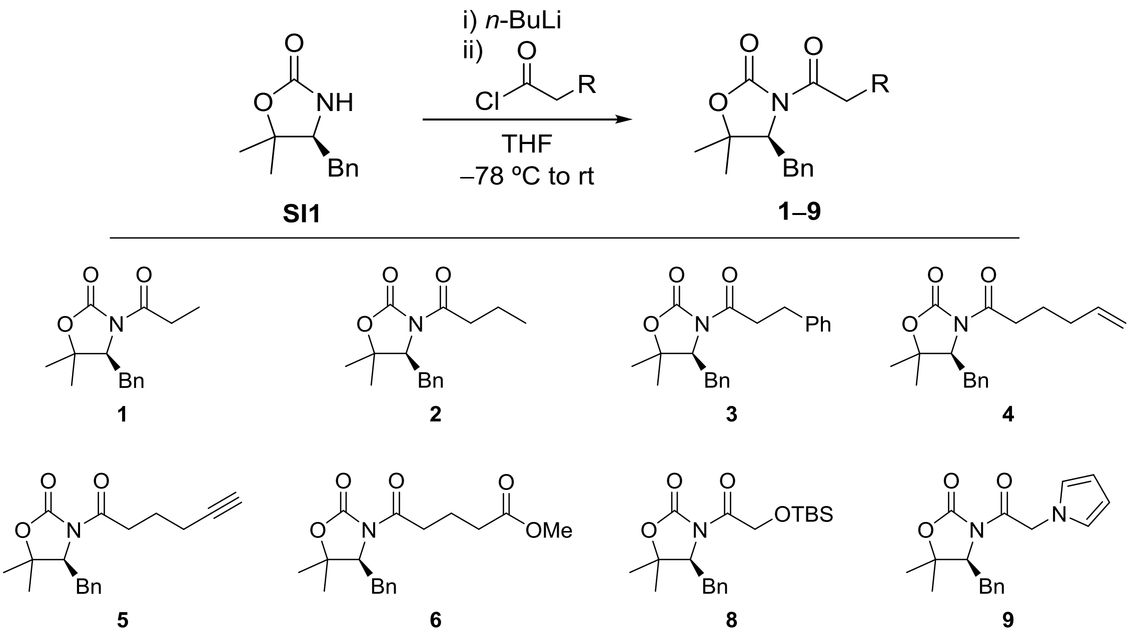

(*S*)-4-Benzyl-5,5-dimethyl-*N*-phenylacetyl-1,3-oxazolidin-2-one (7)

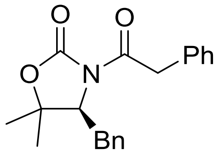

7

A 2.5 M solution of *n*-BuLi in hexanes (1.6 mL, 3.9 mmol) was added dropwise to a solution of **SI1** (616 mg, 3.0 mmol) in THF (15 mL) at −78 °C under nitrogen. The solution was stirred for 15 min and 2-phenylacetyl chloride (1.02 g, 6.6 mmol) was added dropwise. The final mixture was stirred at −78 °C for 20 min, allowed to warm to rt and stirred for 2 h. The reaction mixture was quenched with sat NH_4Cl (10 mL) and concentrated. The residue was partitioned between water and EtOAc (20 mL each). The aqueous layer was extracted with further EtOAc (2 × 20 mL). The combined organic extracts were washed with sat $NaHCO_3$ (50 mL), brine (50 mL), dried with anhydrous $MgSO_4$ and concentrated under reduced pressure. Purification of the crude product by flash column chromatography (60:40 to 40:60 hexanes/CH_2Cl_2) afforded **7** (536 mg, 1.66 mmol, 55% yield) as a white solid. **mp** 71–76 °C; **R_f** (70:30 hexanes/CH_2Cl_2) 0.5; $[\alpha]_D^{20}$ −20.7 (*c* 1.0, $CHCl_3$); **IR** (ATR) ν 3031,

2979, 2928, 1765, 1711, 1396, 1354, 1294, 1277, 1243, 1162, 1099 cm^{-1}; **^{1}H NMR** (400 MHz, $CDCl_3$) δ 7.36–7.17 (10H, m), 4.50 (1H, dd, *J* = 9.6, 3.7 Hz), 4.28 (1H, d, *J* = 15.7 Hz), 4.27 (1H, d, *J* = 15.7 Hz), 3.14 (1H, dd, *J* = 14.4, 3.7 Hz), 2.85 (1H, dd, *J* = 14.4, 9.6 Hz), 1.36 (3H, s), 1.31 (3H, s); **^{13}C NMR** (100.6 MHz, $CDCl_3$) δ 171.4 (C), 152.6 (C), 136.8 (C), 133.6 (C), 129.6 (CH), 129.0 (CH), 128.6 (CH), 128.5 (CH), 127.1 (CH), 126.7 (CH), 82.3 (C), 63.8 (CH), 41.8 (CH_2), 35.2 (CH_2), 28.5 (CH_3), 22.3 (CH_3); **HRMS** (+ESI) *m/z*: $[M+Na]^+$ Calcd for $C_{20}H_{21}NNaO_3$ 346.1414; Found 346.1413.

2.2 Synthesis of *tert*-Butyl Peresters

General Procedure 1. Preparation of *tert*-Butyl Peresters

tert-Butyl Peresters were synthesized according to the literature.[3]

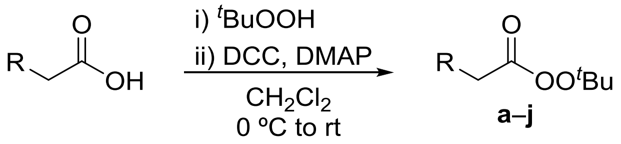

70% ^{t}BuOOH in water (4.5 to 5.0 mmol, 0.9 to 1.0 equiv) was added to a solution of the corresponding carboxylic acid (5.0 mmol, 0.5 M, 1.0 equiv) in CH_2Cl_2 (10 mL) at 0 °C. The resultant solution was vigorously stirred for 10 min at 0 °C. Then, DMAP (61 mg, 0.5 mmol, 0.1 equiv), DCC (1.13 g, 5.5 mmol, 1.1 equiv) and CH_2Cl_2 (5 mL) were added in one portion and the reaction was vigorously stirred at rt for 16 h. After the addition of hexanes (15 mL), the mixture was filtered, dried with anhydrous $MgSO_4$, and concentrated. The residue was purified by short flash column chromatography to afford the *tert*-butyl perester.

tert-Butyl 1-adamantanecarboperoxoate (a)

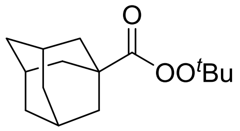

a

It was prepared following General Procedure 1 from 1-adamantanecarboxylic acid (1.98 g, 11.0 mmol), ^{t}BuOOH (1.3 mL, 10.0 mmol), DMAP (134 mg, 1.1 mmol) and DCC (250 g, 12.1 mmol). The residue was purified by flash column chromatography (from 60:40 to 40:60 hexanes/CH_2Cl_2) to afford **a** (1.63 g, 6.5 mmol, 65% yield) as a colorless oil. **R_f** (50:50 hexanes/CH_2Cl_2) 0.3; **IR** (ATR) ν 2980, 2905, 2852, 1762, 1452, 1365, 1168, 1028 cm^{-1}; **^{1}H NMR** (400 MHz, $CDCl_3$) δ 2.03 (3H, br m), 1.97–1.95 (6H, m), 1.76–1.79 (6H, m), 1.32 (9H, s); **^{13}C NMR** (100.6 MHz, $CDCl_3$) δ 174.2 (C), 83.3 (C), 41.1 (C), 38.8 (CH_2), 36.3 (CH_2), 27.8 (CH), 26.1 (CH_3).

***tert*-Butyl 4-pentylbicyclo[2.2.2]octane-1-carboperoxoate (b)**

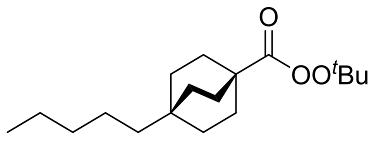

b

It was prepared following General Procedure 1 from 4-pentylbicyclo[2.2.2]octane-1-carboxylic acid (449 mg, 2.0 mmol), ^{t}BuOOH (260 µL, 2.0 mmol), DMAP (24 mg, 0.2 mmol) and DCC (454 mg, 2.2 mmol). The residue was purified by flash column chromatography (95:5 hexanes/EtOAc) to afford **b** (423 mg, 1.4 mmol, 71% yield) as a colorless oil. **R$_f$** (90:10 hexanes/EtOAc) 0.6; **IR** (ATR) ν 2925, 2859, 1765, 1456, 1365, 1184, 1120, 1029 cm^{-1}; **^{1}H NMR** (400 MHz, $CDCl_3$) δ 1.86–1.77 (6H, m), 1.45–1.35 (6H, m), 1.31 (9H, s), 1.27–1.03 (8H, m), 0.87 (3H, t, J = 7.2 Hz); **^{13}C NMR** (100.6 MHz, $CDCl_3$) δ 174.8 (C), 83.3 (C), 41.3 (CH_2), 34.9 (C), 32.7 (CH_2), 30.3 (CH_2), 28.6 (CH_2), 28.1 (C), 26.1 (CH_3), 23.3 (CH_2), 22.6 (CH_2), 14.1 (CH_3).

***tert*-Butyl 2,2-dimethylpropaneperoxoate (c)**

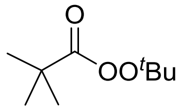

c

It was prepared following General Procedure 1 from pivalic acid (510 mg, 5.0 mmol). The residue was purified by flash column chromatography (95:5 hexanes/EtOAc) to afford **c** (457 mg, 2.6 mmol, 58% yield) as a colorless oil. **R$_f$** (90:10 hexanes/EtOAc) 0.5; **IR** (ATR) ν 2979, 2934, 1765, 1479, 1366, 1192, 1091 cm^{-1}; **^{1}H NMR** (400 MHz, $CDCl_3$) δ 1.33 (9H, s), 1.26 (9H, s); **^{13}C NMR** (100.6 MHz, $CDCl_3$) δ 175.0 (C), 83.3 (C), 38.8 (C), 27.2 (CH_3), 26.1 (CH_3).

***tert*-Butyl 5-(2,5-dimethylphenoxy)-2,2-dimethylpentaneperoxoate (d)**

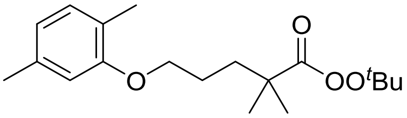

d

It was prepared following General Procedure 1 from 5-(2,5-dimethylphenoxy)-2,2-dimethylpentanoic acid (501 mg, 2.0 mmol), ^{t}BuOOH (260 µL, 2.0 mmol), DMAP (24 mg, 0.2 mmol), and DCC (454 mg, 2.2 mmol). The residue was purified by flash column chromatography (from 80:20 to 50:50 hexanes/CH_2Cl_2) to afford **d** (185 mg, 0.57 mmol, 29% yield) as a white solid. **R$_f$** (70:30 hexanes/CH_2Cl_2) 0.2; **IR** (ATR) ν 2981, 2924, 1758, 1510, 1471, 1366, 1259, 1103, 1038 cm^{-1}; **^{1}H NMR** (400 MHz, $CDCl_3$) δ 7.00 (1H, br d, J = 7.5 Hz), 6.65 (1H, br d, J = 7.5 Hz), 6.61 (1H, br s), 3.93 (2H, t, J = 5.7 Hz), 2.30 (3H, s), 2.17 (3H, s), 1.85–1.72 (4H, m), 1.33 (9H, s), 1.28 (6H, s); **^{13}C NMR** (100.6 MHz, $CDCl_3$) δ 174.3 (C), 156.9 (C), 136.4 (C), 130.3

(CH), 123.5 (C), 120.7 (CH), 111.9 (CH), 83.3 (C), 67.7 (CH_2), 42.3 (C), 37.3 (CH_2), 26.2 (CH_3), 25.2 (CH_3), 25.1 (CH_2), 21.4 (CH_3), 15.7 (CH_3).

***tert*-Butyl isobutaneperoxoate (e)**

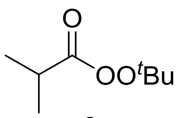

e

It was prepared following General Procedure 1 from isobutyric acid (280 µg, 3.0 mmol), ^{t}BuOOH (390 µL, 3.0 mmol), DMAP (37 mg, 0.3 mmol), and DCC (743 mg, 3.6 mmol). The residue was purified by flash column chromatography (95:5 hexanes/EtOAc) to afford **e** (347 mg, 2.2 mmol, 72% yield) as a colorless oil. $\mathbf{R_f}$ (90:10 hexanes/EtOAc) 0.5; **IR** (ATR) ν 2979, 2937, 2878, 1772, 1470, 1366, 1191, 1104, 1072, 1030 cm^{-1}; **^{1}H NMR** (400 MHz, $CDCl_3$) δ 2.61 (1H, hept, *J* = 7.0 Hz), 1.33 (9H, s), 1.22 (6H, d, *J* = 7.0 Hz); **^{13}C NMR** (100.6 MHz, $CDCl_3$) δ 174.2 (C), 83.3 (C), 31.9 (CH), 26.1 (CH_3), 19.0 (CH_3).

***tert*-Butyl 2-methyl-3-phenylpropaneperoxoate (f)**

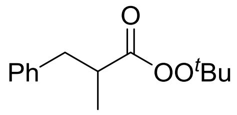

f

It was prepared following General Procedure 1 from 2-methyl-3-phenylpropanoic acid (542 mg, 3.3 mmol), ^{t}BuOOH (390 µL, 3.0 mmol), DMAP (37 mg, 0.3 mmol), and DCC (743 mg, 3.6 mmol). The residue was purified by flash column chromatography (50:50 hexanes/CH_2Cl_2) to afford **f** (648 mg, 2.7 mmol, 92% yield) as a white solid. $\mathbf{R_f}$ (50:50 hexanes/CH_2Cl_2) 0.2; **IR** (ATR) ν 2982, 2933, 1758, 1454, 1366, 1190, 1092 cm^{-1}; **^{1}H NMR** (400 MHz, $CDCl_3$) δ 7.31–7.15 (5H, m), 3.06–2.98 (1H, m), 2.82–2.68 (2H, m), 1.23 (3H, d, *J* = 6.7 Hz), 1.19 (9H, s); **^{13}C NMR** (100.6 MHz, $CDCl_3$) δ 173.3 (C), 138.7 (C), 129.0 (CH), 128.5 (CH), 126.5 (CH), 83.3 (C), 39.7 (CH_2), 39.4 (CH), 26.0 (CH_3), 17.3 (CH_3).

***tert*-Butyl 2-ethylbutaneperoxoate (g)**

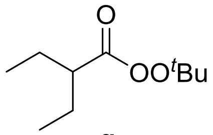

g

It was prepared following General Procedure 1 from 2-ethylbutyric acid (380 µL, 3.0 mmol), ^{t}BuOOH (390 µL, 3.0 mmol), DMAP, (37 mg, 0.3 mmol) and DCC (743 mg, 3.6 mmol). The residue was purified by flash column chromatography (95:5 hexanes/EtOAc) to afford **g** (481 mg, 2.6 mmol, 85% yield) as a colorless oil. $\mathbf{R_f}$ (90:10 hexanes/EtOAc) 0.7; **IR** (ATR) ν 2967, 2934, 2878, 1770, 1460, 1365, 1190, 1071 cm^{-1}; **^{1}H NMR** (400 MHz,

$CDCl_3$) δ 2.22 (1H, tt, *J* = 8.8, 5.4 Hz), 1.74–1.61 (2H, m), 1.61–1.49 (2H, m), 1.34 (9H, s), 0.94 (6H, t, *J* = 7.4 Hz); **^{13}C NMR** (100.6 MHz, $CDCl_3$) δ 173.4 (C), 82.9 (C), 46.7 (CH), 26.2 (CH_3), 25.2 (CH_2), 11.8 (CH_3).

tert-Butyl cyclopentanecarboperoxoate (h)

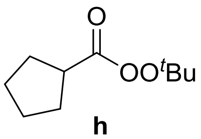

h

It was prepared following General Procedure 1 from cyclopentanecarboxylic acid (360 µL, 3.3 mmol), ^{t}BuOOH (390 µL, 3.0 mmol), DMAP (37 mg, 0.3 mmol), and DCC (743 mg, 3.6 mmol). The residue was purified by flash column chromatography (50:50 hexanes/CH_2Cl_2) to afford **h** (499 mg, 2.7 mmol, 89% yield) as a colorless oil. **R_f** (60:40 hexanes/CH_2Cl_2) 0.2; **IR** (ATR) ν 2976, 2872, 1769, 1365, 1190, 1094 cm^{-1}; **^{1}H NMR** (400 MHz, $CDCl_3$) δ 2.74 (1H, p, *J* = 8.0 Hz), 1.99–1.68 (6H, m), 1.65–1.55 (2H, m), 1.32 (9H, s); **^{13}C NMR** (100.6 MHz, $CDCl_3$) δ 173.9 (C), 83.1 (C), 40.9 (CH), 30.1 (CH_2), 26.0 (CH_3), 25.7 (CH_2).

tert-Butyl cyclohexanecarboperoxoate (i)

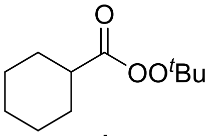

i

It was prepared following General Procedure 1 from cyclohexanecarboxylic acid (641 mg, 5.0 mmol). The residue was purified by flash column chromatography (50:50 hexanes/CH_2Cl_2) to afford **i** (843 mg, 4.0 mmol, 88% yield) as a colorless oil. **R_f** (50:50 hexanes/CH_2Cl_2) 0.3; **IR** (ATR) ν 2981, 2932, 2856, 1768, 1451, 1365, 1188, 1146, 1084, 1014 cm^{-1}; **^{1}H NMR** (400 MHz, $CDCl_3$) δ 2.37 (1H, tt, *J* = 11.4, 3.7 Hz), 1.94–1.85 (2H, m), 1.81–1.75 (2H, m), 1.68–1.63 (1H, m), 1.59–1.45 (2H, m), 1.36–1.31 (1H, m), 1.32 (9H, s), 1.30–1.24 (2H, m); **^{13}C NMR** (100.6 MHz, $CDCl_3$) δ 172.9 (C), 83.1 (C), 41.0 (CH), 29.0 (CH_2), 26.0 (CH_3), 25.4 (CH_2), 25.2 (CH_2).

tert-Butyl tetrahydropyrane-4-carboperoxoate (j)

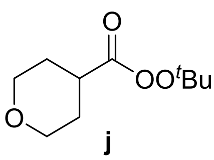

j

It was prepared following General Procedure 1 from tetrahydropyrane-4-carboxylic acid (390 mg, 3.0 mmol), ^{t}BuOOH (390 µL, 3.0 mmol), DMAP (37 mg, 0.3 mmol), and DCC (743 mg, 3.6 mmol). The residue was purified by flash column chromatography (90:10 hexanes/EtOAc) to afford **j** (506 mg, 2.5 mmol, 83% yield) as

a colorless oil. **R$_f$** (90:10 hexanes/EtOAc) 0.2; **IR** (ATR) ν 2979, 2847, 1768, 1446, 1366, 1241, 1189, 1122, 1087, 1027 cm^{-1}; **^{1}H NMR** (400 MHz, $CDCl_3$) δ 3.98 (2H, dt, *J* = 11.6, 3.5 Hz), 3.49–3.40 (2H, m), 2.64 (1H, tt, *J* = 10.1, 5.2 Hz), 1.94–1.79 (4H, m), 1.33 (9H, s); **^{13}C NMR** (100.6 MHz, $CDCl_3$) δ 171.5 (C), 83.5 (C), 66.8 (CH_2), 38.3 (CH), 28.6 (CH_2), 26.1 (CH_3).

3 Optimization Tables

3.1 Screening of the Reaction Conditions with 1a as Model Substrate

Neat $TiCl_4$ (61 µL, 0.55 mmol, 1.1 equiv) was added dropwise to a solution of *N*-acyl oxazolidinone **1** (0.50 mmol, 0.25 M, 1.0 equiv) in DCE (2 mL) at 0 °C. After 5 min, R_3N (2.5 mmol, 2.5 equiv) was added dropwise and the resultant deep purple mixture was stirred at 0 °C for 40 min. A solution of the *tert*-butyl perester **a** (1.5–2.0 equiv) in DCE (1 mL) was added via *cannula*. The reaction was stirred at "Temperature" for 1 to 2 h. Then, it was quenched with sat NH_4Cl (3 mL). The layers were separated (2 M HCl could be added to facilitate the separation) and the aqueous layer was extracted with CH_2Cl_2 (2 × 10 mL). The combined organic extracts were dried with anhydrous $MgSO_4$ and the volatiles evaporated. The residue was purified by flash column chromatography to afford **1a** as a single diastereomer.

Table 1. Screening of the reaction conditions

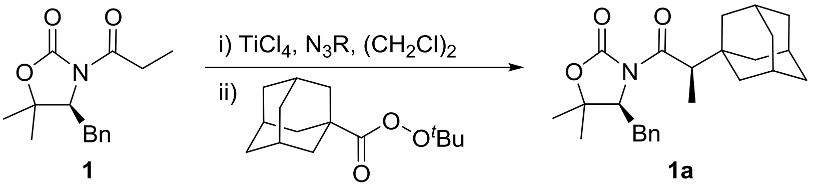

Entry	R_3N	Equiv. of Perester	Temperature	Time	Yield
1	Et_3N	1.5	rt	1.5 h	74%
2		2.0		1.5 h	62%
3		1.5	0 °C	2 h	58%
4		2.0		2 h	60%
5	*i*-Pr_2NEt	1.5	rt	1 h	66%

3.2 Attempts to Minimize By-Product SI2

The same procedure was applied to synthesis of **1c**. However, by-product **SI2** (two diastereomers) was obtained in a significant amount together with the desired product, resulting in low yields. Changes in the conditions were attempted to minimize the formation of **SI2**, without any success.

Other unsuccessful changes, in which **1c** was not isolated, include the use of different equivalents of perester **c**, addition of different equivalents of $TiCl_4$, or different reaction temperatures.

Table 2. Changes to minimize formation of SI2

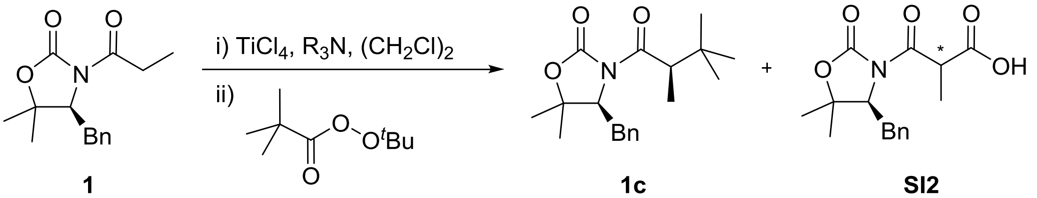

1 1c SI2

Entry	Change from standard conditions	Yield 1c
1	-	48%
2	16 h	42%
3	*i*-Pr_2NEt, 16 h	40%
4	DMA, 16 h	35%

(*S*)-4-Benzyl-*N*-[(2-carboxypropanoyl]- 5,5-dimethyl-1,3-oxazolidin-2-one (SI2)

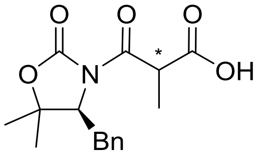

SI2

Characterization of one diastereomer. White solid; **R_f** (50:50 hexanes/EtOAc) 0.2; **IR** (ATR) ν br 3500–2400, 2931, 1772, 1698, 1455, 1362, 1276, 1237, 1208, 1182 1160, 1080 cm^{-1}; **^{1}H NMR** (400 MHz, $CDCl_3$) δ 7.37–7.13 (5H, m), 4.59 (1H, q, J = 7.2 Hz), 4.53 (1H, dd, J = 10.3, 2.9 Hz), 3.27 (1H, dd, J = 14.7, 2.9 Hz), 2.90 (1H, dd, J = 14.7, 10.3 Hz), 1.52 (3H, d, J = 7.2 Hz), 1.37 (6H, s); **^{13}C NMR** (100.6 MHz, $CDCl_3$) δ 175.2 (C), 169.4 (C), 152.6 (C), 137.0 (C), 129.0 (CH), 128.7 (CH), 126.7 (CH), 82.9 (C), 64.0 (CH), 45.5 (CH), 34.6 (CH_2), 28.7 (CH_3), 22.4 (CH_3), 13.2 (CH_3); **HRMS** (+ESI) *m/z*: $[M+H]^+$ Calcd for $C_{16}H_{20}NO_5$ 306.1336; Found 306.1345.

4 Scope of the Alkylation Reaction

General Procedure 2. Alkylation with *tert*-Butyl Peresters

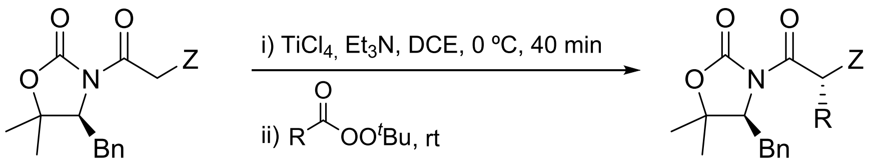

1-9 **1a–j, 2a–9a**

Neat $TiCl_4$ (61 µL, 0.55 mmol, 1.1 equiv) was added dropwise to a solution of *N*-acyl oxazolidinone (**1−9**) (0.50 mmol, 0.25 M, 1.0 equiv) in DCE (2 mL) at 0 °C. After 5 min, Et_3N (210 to 350 µL, 1.5 to 2.5 mmol, 3.0 to 5.0 equiv) was added dropwise and the resultant deep purple mixture was stirred at 0 °C for 40 min. A solution of the *tert*-butyl perester (**a−j**) (0.75 mmol, 0.75 M, 1.5 equiv) in DCE (1 mL) was added via *cannula*. The reaction was allowed to warm to rt and stirred for 1.5 to 12 h. Then, it was quenched with sat NH_4Cl (3 mL). The layers were separated (2 M HCl could be added to facilitate the separation) and the aqueous layer was extracted with CH_2Cl_2 (2 × 10 mL). The combined organic extracts were dried with anhydrous $MgSO_4$ and the solvent evaporated. The residue was purified by flash column chromatography to afford the corresponding alkylated products as a single diastereomer.

4.1 Alkylation of 1 with *tert*-Butyl Peresters a−j (Scheme 2)

(*S*)-*N*-[(*R*)-2-Adamantylpropanoyl]-4-benzyl-5,5-dimethyl-1,3-oxazolidin-2-one (1a)

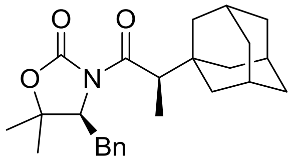

1a

It was prepared following General Procedure 2 from *N*-acyl oxazolidinone **1** (261 mg, 1.0 mmol), $TiCl_4$ (121 µL, 1.1 mmol), Et_3N (420 µL, 3.0 mmol) and *tert*-butyl perester **a** (379 mg, 1.5 mmol) for 90 min. Purification of the residue by flash column chromatography (95:5 hexanes/EtOAc) afforded **1a** (295 mg, 0.74 mmol, 74% yield) as a white solid. **mp** 132–133 °C; **R$_f$** (90:10 hexanes/EtOAc) 0.5; $[\alpha]_D^{20}$ −56.9 (*c* 1.0, $CHCl_3$); **IR** (ATR) ν 2906, 2846, 1773, 1685, 1454 cm^{-1}; **^{1}H NMR** (400 MHz, $CDCl_3$) δ 7.38−7.17 (5H, m), 4.57 (1H, dd, *J* = 10.3, 3.2 Hz), 3.80 (1H, q, *J* = 7.0 Hz), 3.27 (1H, dd, *J* = 14.4, 3.2 Hz), 2.84 (1H, dd, *J* = 14.4, 10.3 Hz), 1.98−1.93 (3H, m), 1.82−1.77 (3H, m), 1.70−1.57 (6H, m), 1.54−1.51 (3H, m), 1.34 (3H, s), 1.30 (3H, s), 1.10 (3H, d, *J* = 7.0 Hz); **^{13}C NMR** (100.6 MHz, $CDCl_3$) δ 176.9 (C), 152.7 (C), 137.1 (C), 129.0 (CH), 128.6 (CH), 126.7 (CH), 81.3 (C), 64.0 (CH), 45.5 (CH), 39.3 (CH_2), 36.9 (CH_2), 35.9 (C), 35.3 (CH_2), 28.6 (CH),

28.5 (CH_3), 22.5 (CH_3), 11.4 (CH_3); **HRMS** (+ESI) *m/z*: $[M+H]^+$ Calcd for $C_{25}H_{34}NO_3$ 396.2533; Found 396.2534.

(*S*)-4-Benzyl-5,5-dimethyl-*N*-[(*R*)-2-(4-pentylbicyclo[2.2.2]octan-1-yl)propanoyl]-1,3-oxazolidin-2-one (1b)

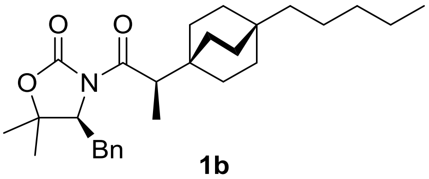

1b

It was prepared following General Procedure 2 from *N*-acyl oxazolidinone **1** (131 mg, 0.5 mmol), $TiCl_4$ (61 µL, 0.55 mmol), Et_3N (210 µL, 1.50 mmol) and peroxide **b** (222 mg, 0.75 mmol) for 4 h. Purification of the residue by flash column chromatography (from 99:1 to 95:5 hexanes/EtOAc) afforded **1b** (86 mg, 0.19 mmol, 39% yield) as a colorless oil. $\mathbf{R_f}$ (90:10 hexanes/EtOAc) 0.4; $[\alpha]_D^{20}$ −63.6 (*c* 1.0, $CHCl_3$); **IR** (ATR) ν 2925, 2856, 1771, 1691, 1455, 1376, 1351, 1274, 1207, 1178, 1095 cm^{-1}; **^{1}H NMR** (400 MHz, $CDCl_3$) δ 7.34–7.20 (5H, m), 4.55 (1H, dd, *J* = 10.3, 3.3 Hz), 3.82 (1H, q, *J* = 7.0 Hz), 3.22 (1H, dd, *J* = 14.4, 3.3 Hz), 2.83 (1H, dd, *J* = 14.4, 10.3 Hz), 1.68–1.56 (3H, m), 1.44–1.24 (11H, m), 1.33 (3H, s), 1.30 (3H, s), 1.23–1.10 (4H, m), 1.07 (3H, d, *J* = 7.0 Hz), 1.04–0.99 (2H, m), 0.86 (3H, t, *J* = 7.2 Hz); **^{13}C NMR** (100.6 MHz, $CDCl_3$) δ 177.1 (C), 152.6 (C), 137.1 (C), 129.0 (CH), 128.6 (CH), 126.7 (CH), 81.3 (C), 63.9 (CH), 43.5 (CH), 41.6 (CH_2), 35.3 (CH_2), 34.6 (C), 32.8 (CH_2), 31.0 (CH_2), 30.2 (C), 28.5 (CH_3), 28.4 (CH_2), 23.3 (CH_2), 22.7 (CH_2), 22.4 (CH_3), 14.1 (CH_3), 12.5 (CH_3); **HRMS** (+ESI) *m/z*: $[M+H]^+$ Calcd for $C_{28}H_{42}NO_3$ 440.3159; Found 440.3152.

(*S*)-4-Benzyl-*N*-[(*R*)-2-*tert*-butylpropanoyl]-5,5-dimethyl-1,3-oxazolidin-2-one (1c)

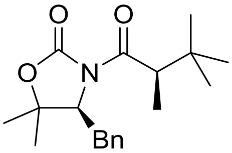

1c

It was prepared following General Procedure 2 from *N*-acyl oxazolidinone **1** (131 mg, 0.5 mmol), $TiCl_4$ (61 µL, 0.55 mmol), Et_3N (210 µL, 1.5 mmol) and *tert*-butyl perester **c** (131 mg, 0.75 mmol) for 90 min. Purification of the residue by flash column chromatography (95:5 hexanes/EtOAc) afforded **1c** (77 mg, 0.24 mmol, 48% yield) as a white solid. **mp** 95–97 °C; $\mathbf{R_f}$ (90:10 hexanes/EtOAc) 0.5; $[\alpha]_D^{20}$ −52.7 (*c* 1.0, $CHCl_3$); **IR** (ATR) ν 2967, 1770, 1692, 1376, 1231, 1275, 1231, 1175, 1103, 1082 cm^{-1}; **^{1}H NMR** (400 MHz, $CDCl_3$) δ 7.35–7.18 (5H, m), 4.56 (1H, dd, *J* = 10.5, 3.0 Hz), 3.93 (1H, q, *J* = 7.0 Hz), 3.25 (1H, dd, *J* = 14.3, 3.0 Hz), 2.82 (1H, dd, *J* = 14.3, 10.5 Hz), 1.33 (3H, s), 1.30 (3H, s), 1.14 (3H, d, *J* = 7.0 Hz), 1.00 (9H, s); **^{13}C NMR** (100.6 MHz, $CDCl_3$) δ 177.3 (C), 152.6 (C), 137.1 (C), 128.9 (CH), 128.7 (CH), 126.7 (CH), 81.3 (C), 64.0 (CH),

44.7 (CH), 35.1 (CH_2), 33.9 (C), 28.6 (CH_3), 27.4 (CH_3), 22.5 (CH_3), 13.1 (CH_3); **HRMS** (+ESI) *m/z*: $[M+H]^+$ Calcd for $C_{19}H_{28}NO_3$ 318.2064; Found 318.2067.

(*S*)-4-Benzyl-5,5-dimethyl-*N*-[(*R*)-6-(2,5-dimethylphenoxy)-2,3,3-trimethylhexanoyl]-1,3-oxazolidin-2-one (1d)

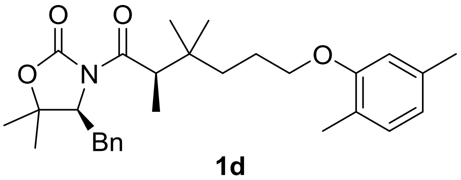

1d

It was prepared following General Procedure 2 from *N*-acyl oxazolidinone **1** (131 mg, 0.5 mmol), $TiCl_4$ (61 µL, 0.55 mmol), Et_3N (210 µL, 1.50 mmol) and peroxide **d** (242 mg, 0.75 mmol) for 2.5 h. Purification of the residue by flash column chromatography (from 99:1 to 95:5 hexanes/EtOAc) afforded **1d** (54 mg, 0.12 mmol, 23% yield) as a colorless oil. $\mathbf{R_f}$ (95:5 hexanes/EtOAc) 0.4; $[\alpha]_D^{20}$ −52.8 (*c* 1.0, $CHCl_3$); **IR** (ATR) ν 2962, 1770, 1693, 1508, 1376, 1350, 1265, 1234, 1207, 1177, 1129, 1103 cm^{-1}; ^{1}**H NMR** (400 MHz, $CDCl_3$) δ 7.33–7.17 (5H, m), 6.98 (1H, d, *J* = 7.5 Hz), 6.65 (1H, d, *J* = 7.5 Hz), 6.62 (1H, s), 4.56 (1H, dd, *J* = 10.5, 3.0 Hz), 4.06 (1H, q, *J* = 7.0 Hz), 3.92 (2H, t, *J* = 6.3 Hz), 3.24 (1H, dd, *J* = 14.4, 3.0 Hz), 2.77 (1H, dd, *J* = 14.4, 10.5 Hz), 2.30 (3H, s), 2.15 (3H, s), 1.85–1.76 (2H, m), 1.60–1.48 (2H, m), 1.31 (3H, s), 1.30 (3H, s), 1.16 (3H, d, *J* = 7.0 Hz), 1.03 (3H, s), 1.01 (3H, s); ^{13}**C NMR** (100.6 MHz, $CDCl_3$) δ 177.2 (C), 157.1 (C), 152.6 (C), 137.1 (C), 136.4 (C), 130.2 (C), 128.9 (CH), 128.6 (CH), 126.7 (CH), 123.6 (CH), 120.6 (CH), 112.0 (CH), 81.4 (C), 68.4 (CH_2), 64.0 (CH), 43.4 (CH), 36.6 (CH_2), 36.2 (CH_2), 35.1 (CH_2), 28.6 (CH_3), 24.2 (CH_3), 24.2 (CH_3), 24.0 (CH_2), 22.5 (CH_3), 21.4 (CH_3), 15.8 (CH_3), 12.8 (CH_3); **HRMS** (+ESI) *m/z*: $[M+Na]^+$ Calcd for $C_{29}H_{39}NNaO_4$ 488.2771; Found 488.2762.

(*S*)-4-Benzyl-*N*-[(*R*)-2,3-dimethylbutanoyl]-5,5-dimethyl-1,3-oxazolidin-2-one (1e)

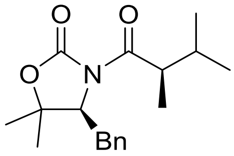

1e

It was prepared following General Procedure 2 from *N*-acyl oxazolidinone **1** (131 mg, 0.50 mmol), $TiCl_4$ (61 µL, 0.55 mmol), Et_3N (210 µL, 0.75 mmol) and *tert*-butyl perester **e** (120 mg, 0.75 mmol) for 2.5 h. Purification of the residue by flash column chromatography (from 99:1 to 95:5 hexanes/EtOAc) afforded **1e** (97 mg, 0.35 mmol, 64% yield) as a colorless oil. $\mathbf{R_f}$ (90:10 hexanes/EtOAc) 0.3; $[\alpha]_D^{20}$ −53.8 (*c* 1.0, $CHCl_3$); **IR** (ATR) ν 3024, 2964, 2926, 2869, 1767, 1688 cm^{-1}; ^{1}**H NMR** (400 MHz, $CDCl_3$) δ 7.33–7.20 (5H, m), 4.56 (1H, dd, *J* = 10.0, 3.5 Hz), 3.65 (1H, quint, *J* = 6.8 Hz), 3.16 (1H, dd, *J* = 14.4, 3.5 Hz), 2.85 (1H, dd, *J* = 14.4, 10.0 Hz), 2.03 (1H, oct, *J* = 6.8 Hz), 1.35 (3H, s), 1.33 (3H, s), 1.11 (3H, d, *J* = 6.8 Hz), 0.96 (3H, d, *J* = 6.8 Hz),

0.87 (3H, d, *J* = 6.8 Hz); **^{13}C NMR** (100.6 MHz, $CDCl_3$) δ 177.4 (C), 152.4 (C), 137.0 (C), 129.0 (CH), 128.6 (CH), 126.7 (CH), 81.7 (C), 63.7 (CH), 43.3 (CH), 35.3 (CH_2), 30.8 (CH), 28.6 (CH_3), 22.4 (CH_3), 21.0 (CH_3), 18.2 (CH_3), 12.9 (CH_3); **HRMS** (+ESI) *m/z*: $[M+H]^+$ Calcd for $C_{18}H_{26}NO_3$ 304.1907; Found 304.1914.

(4*S*)-4-Benzyl-*N*-[(2*R*)-2,3-dimethyl-4-phenylbutanoyl]-5,5-dimethyl-1,3-oxazolidin-2-one (1f)

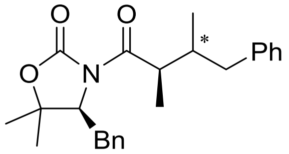

1f

It was prepared following General Procedure 2 from *N*-acyl oxazolidinone **1** (131 mg, 0.50 mmol), $TiCl_4$ (61 μL, 0.55 mmol), Et_3N (210 μL, 0.75 mmol) and *tert*-butyl perester **f** (177 mg, 0.75 mmol) for 1 h. Purification of the residue by flash column chromatography (from 99:1 to 95:5 hexanes/EtOAc) afforded **1f** (147 mg, 0.39 mmol, 77% yield, d.r. 2:1)) as a colorless oil. The diastereomers were separated by a second flash column chromatography (from 60:40 to 35:65 hexanes/CH_2Cl_2) and characterized individually.

Major diastereomer

R$_f$ (70:30 hexanes/CH_2Cl_2) 0.2; $[\alpha]_D^{20}$ −58.7 (*c* 1.0, $CHCl_3$); **IR** (ATR) ν 2968, 2926, 1771, 1685, 1495, 1454, 1359, 1232, 1101 cm^{-1}; **^{1}H NMR** (400 MHz, $CDCl_3$) δ 7.32–7.11 (10H, m), 4.57 (1H, dd, *J* = 9.9, 3.6 Hz), 3.80 (1H, qd, *J* = 6.9, 5.6 Hz), 3.11 (1H, dd, *J* = 14.4, 3.6 Hz), 2.84 (1H, dd, *J* = 14.4, 9.9 Hz), 2.77 (1H, dd, *J* = 12.9, 3.9 Hz), 2.35 (1H, dd, *J* = 12.9, 10.3 Hz), 2.26–2.16 (1H, m), 1.35 (3H, s), 1.34 (3H, s), 1.15 (3H, d, *J* = 6.9 Hz), 0.74 (3H, d, *J* = 6.8 Hz); **^{13}C NMR** (100.6 MHz, $CDCl_3$) δ 177.0 (C), 152.3 (C), 140.8 (C), 136.9 (C), 129.3 (CH), 129.0 (CH), 128.7 (CH), 128.1 (CH), 126.8 (CH), 125.9 (CH), 81.8 (C), 63.6 (CH), 42.7 (CH), 41.9 (CH_2), 37.5 (CH), 35.4 (CH_2), 28.7 (CH_3), 22.4 (CH_3), 14.2 (CH_3), 12.2 (CH_3); **HRMS** (+ESI) *m/z*: $[M+H]^+$ Calcd for $C_{24}H_{30}NO_3$ 380.2220; Found 380.2224.

Minor diastereomer

R$_f$ (70:30 hexanes/EtOAc) 0.2; $[\alpha]_D^{20}$ −55.4 (*c* 1.0, $CHCl_3$); **IR** (ATR) ν 2970, 2933, 1770, 1692, 1496, 1454, 1350, 1234, 1089 cm^{-1}; **^{1}H NMR** (400 MHz, $CDCl_3$) δ 7.33–7.09 (10H, m), 4.57 (1H, dd, *J* = 10.1, 3.3 Hz), 3.84 (1H, qd, *J* = 6.8, 5.2 Hz), 3.18 (1H, dd, *J* = 14.3, 3.3 Hz), 2.91–2.80 (2H, m), 2.26–2.11 (2H, m), 1.36 (3H, s), 1.34 (3H, s), 1.25 (3H, d, *J* = 6.8 Hz), 0.85 (3H, d, *J* = 6.3 Hz); **^{13}C NMR** (100.6 MHz, $CDCl_3$) δ 177.1 (C), 152.4 (C), 140.7 (C), 137.0 (C), 129.1 (CH), 129.0 (CH), 128.7 (CH), 128.2 (CH), 126.8 (CH), 125.8 (CH), 81.8 (C), 63.8 (CH), 42.7 (CH), 38.7 (CH_2), 37.9 (CH), 35.3 (CH_2), 28.7 (CH_3), 22.4 (CH_3), 17.2 (CH_3), 13.4 (CH_3); **HRMS** (+ESI) *m/z*: $[M+H]^+$ Calcd for $C_{24}H_{30}NO_3$ 380.2220; Found 380.2221.

(*S*)-4-Benzyl-*N*-[(*R*)-3-ethyl-2-methylpentanoyl]-5,5-dimethyl-1,3-oxazolidin-2-one (1g)

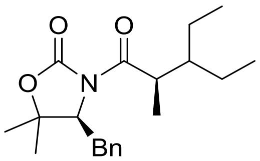

1g

It was prepared following General Procedure 2 from *N*-acyl oxazolidinone **1** (131 mg, 0.50 mmol), $TiCl_4$ (61 µL, 0.55 mmol), Et_3N (210 µL, 0.75 mmol) and *tert*-butyl perester **g** (141 mg, 0.75 mmol) for 2.5 h. Purification of the residue by flash column chromatography (from 99:1 to 95:5 hexanes/EtOAc) afforded **1g** (96 mg, 0.29 mmol, 58% yield) as a colorless oil. **R_f** (90:10 hexanes/EtOAc) 0.4; $[\alpha]_D^{20}$ −44.7 (*c* 1.0, $CHCl_3$); **IR** (ATR) ν 2963, 2933, 1770, 1694, 1351, 1275, 1231, 1097 cm^{-1}; **^{1}H NMR** (400 MHz, $CDCl_3$) δ 7.31–7.29 (4H, m), 7.26–7.20 (1H, m), 4.56 (1H, dd, *J* = 10.1, 3.4 Hz), 3.88 (1H, quint, *J* = 6.8 Hz), 3.16 (1H, dd, *J* = 14.3, 3.4 Hz), 2.83 (1H, dd, *J* = 14.3, 10.1 Hz), 1.74–1.62 (1H, m), 1.52–1.36 (2H, m), 1.35 (3H, s), 1.31 (3H, s), 1.29–1.17 (2H, m), 1.08 (3H, d, *J* = 6.8 Hz), 0.92 (3H, t, *J* = 7.4 Hz), 0.85 (3H, t, *J* = 7.5 Hz); **^{13}C NMR** (100.6 MHz, $CDCl_3$) δ 178.0 (C), 152.3 (C), 137.0 (C), 129.0 (CH), 128.6 (CH), 126.7 (CH), 81.6 (C), 63.7 (CH), 42.8 (CH), 39.7 (CH), 35.2 (CH_2), 28.7 (CH_3), 23.6 (CH_2), 22.5 (CH_3), 21.3 (CH_2), 12.6 (CH_3), 11.2 (CH_3), 11.1 (CH_3); **HRMS** (+ESI) *m/z*: $[M+Na]^+$ Calcd for $C_{20}H_{29}NNaO_3$ 354.2040; Found 354.2047.

(*S*)-4-Benzyl-*N*-[(*R*)-2-cyclopentylpropanoyl]-5,5-dimethyl-1,3-oxazolidin-2-one (1h)

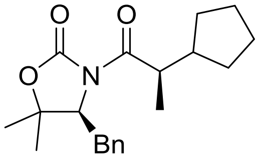

1h

It was prepared following General Procedure 2 from *N*-acyl oxazolidinone **1** (131 mg, 0.50 mmol), $TiCl_4$ (61 µL, 0.55 mmol), Et_3N (210 µL, 0.75 mmol) and *tert*-butyl perester **h** (140 mg, 0.75 mmol) for 1 h. Purification of the residue by flash column chromatography (from 99:1 to 95:5 hexanes/EtOAc) afforded **1h** (86 mg, 0.26 mmol, 52% yield) as a colorless oil. **R_f** (90:10 hexanes/EtOAc) 0.2; $[\alpha]_D^{20}$ −55.5 (*c* 1.0, $CHCl_3$); **IR** (ATR) ν 3027, 2945, 2857, 1770, 1688, 1270, 1169, 1093 cm^{-1}; **^{1}H NMR** (400 MHz, $CDCl_3$) δ 7.33–7.20 (5H, m), 4.54 (1H, dd, *J* = 10.1, 3.3 Hz), 3.72 (1H, dq, *J* = 9.1, 6.8 Hz), 3.18 (1H, dd, *J* = 14.4, 3.3 Hz), 2.85 (1H, dd, *J* = 14.4, 10.1 Hz), 2.17–2.06 (1H, m), 1.81–1.46 (6H, m), 1.35 (3H, s), 1.33 (3H, s), 1.26–1.14 (2H, m), 1.18 (3H, d, *J* = 6.8 Hz); **^{13}C NMR** (100.6 MHz, $CDCl_3$) δ 177.9 (C), 152.4 (C), 137.0 (C), 129.0 (CH), 128.6 (CH), 126.7 (CH), 81.7 (C), 63.8 (CH), 43.4 (CH), 42.4 (CH), 35.2 (CH_2), 30.8 (CH_2), 29.8 (CH_2), 28.6 (CH_3), 25.2 (CH_2), 25.0 (CH_2), 22.4 (CH_3), 16.2 (CH_3); **HRMS** (+ESI) *m/z*: $[M+H]^+$ Calcd for $C_{20}H_{28}NO_3$ 330.2064; Found 330.2072.

(*S*)-4-Benzyl-*N*-[(*R*)-2-cyclohexylpropanoyl]-5,5-dimethyl-1,3-oxazolidin-2-one (1i)

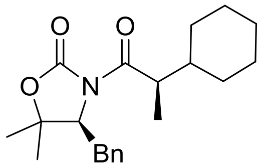

1i

It was prepared following General Procedure 2 from *N*-acyl oxazolidinone **1** (131 mg, 0.50 mmol), $TiCl_4$ (61 µL, 0.55 mmol), Et_3N (210 µL, 0.75 mmol) and *tert*-butyl perester **i** (150 mg, 0.75 mmol) for 1 h. Purification of the residue by flash column chromatography (95:5 hexanes/EtOAc) afforded **1i** (104 mg, 0.30 mmol, 60% yield) as a colorless oil. $\mathbf{R_f}$ (90:10 hexanes/EtOAc) 0.4; $[\alpha]_D^{20}$ −71.3 (*c* 1.0, $CHCl_3$); **IR** (ATR) ν 2923, 2851, 1768, 1689, 1451, 1375, 1347, 1274, 1236, 1201, 1093 cm^{-1}; **^{1}H NMR** (400 MHz, $CDCl_3$) δ 7.33–7.20 (5H, m), 4.56 (1H, dd, *J* = 9.9, 3.6 Hz), 3.68 (1H, quint, *J* = 6.9 Hz), 3.15 (1H, dd, *J* = 14.3, 3.6 Hz), 2.87 (1H, dd, *J* = 14.3, 9.9 Hz), 1.75–1.59 (6H, m), 1.36 (3H, s), 1.33 (3H, s), 1.29–1.13 (3H, m), 1.12–1.05 (1H, m), 1.11 (3H, d, *J* = 6.9 Hz), 1.03–0.93 (1H, m); **^{13}C NMR** (100.6 MHz, $CDCl_3$) δ 177.5 (C), 152.4 (C), 137.0 (C), 129.0 (CH), 128.6 (CH), 126.7 (CH), 81.7 (C), 63.7 (CH), 42.6 (CH), 40.8 (CH), 35.4 (CH_2), 31.3 (CH_2), 28.7 (CH_2), 28.6 (CH_3), 26.4 (CH_2), 26.3 (CH_2), 26.3 (CH_2), 22.4 (CH_3), 13.6 (CH_3); **HRMS** (+ESI) *m/z*: $[M+H]^+$ Calcd for $C_{21}H_{30}NO_3$ 344.2220; Found 344.2222.

(*S*)-4-Benzyl-5,5-dimethyl-*N*-[(*R*)-2-(tetrahydro-4-pyranyl)propanoyl]-1,3-oxazolidin-2-one (1j)

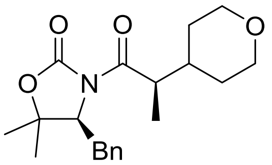

1j

It was prepared following General Procedure 2 from *N*-acyl oxazolidinone **1** (131 mg, 0.5 mmol), $TiCl_4$ (61 µL, 0.55 mmol), Et_3N (210 µL, 1.5 mmol) and *tert*-butyl perester **j** (152 mg, 0.75 mmol) for 2 h. Purification of the residue by flash column chromatography (from 90:10 to 85:15 hexanes/EtOAc) afforded **1j** (74 mg, 0.21 mmol, 43% yield) as a colorless oil. $\mathbf{R_f}$ (90:10 hexanes/EtOAc) 0.3; $[\alpha]_D^{20}$ −66.5 (*c* 1.0, $CHCl_3$); **IR** (ATR) ν 2936, 2842, 1769, 1692, 1375, 1351, 1275, 1207, 1089 cm^{-1}; **^{1}H NMR** (400 MHz, $CDCl_3$) δ 7.34–7.19 (5H, m), 4.57 (1H, dd, *J* = 9.5, 4.1 Hz), 4.02–3.95 (1H, m), 3.91 (1H, dt, *J* = 11.3, 3.0 Hz), 3.70 (1H, p, *J* = 6.9 Hz), 3.42–3.29 (2H, m), 3.11 (1H, dd, *J* = 14.3, 4.1 Hz), 2.89 (1H, dd, *J* = 14.3, 9.5 Hz), 1.96–1.80 (1H, m), 1.52–1.38 (4H, m), 1.38 (3H, s), 1.35 (3H, s), 1.13 (3H, d, *J* = 6.9 Hz); **^{13}C NMR** (100.6 MHz, $CDCl_3$) δ 176.6 (C), 152.4 (C), 136.7 (C), 129.0 (CH), 128.6 (CH), 126.8 (CH), 81.9 (C), 68.0 (CH_2), 67.9 (CH_2), 63.5 (CH), 42.2 (CH), 37.9 (CH), 35.5 (CH_2), 30.9 (CH_2), 28.9 (CH_2), 28.5 (CH_3), 22.2 (CH_3), 13.5 (CH_3); **HRMS** (+ESI) *m/z*: $[M+Na]^+$ Calcd for $C_{20}H_{27}NNaO_4$ 368.1832; Found 368.1837.

4.2 Alkylation of 1–9 with *tert*-Butyl 1-Adamantane Carboperoxoate a (Scheme 3)

(*S*)-*N*-[(*R*)-2-Adamantylbutanoyl]-4-benzyl-5,5-dimethyl-1,3-oxazolidin-2-one (2a)

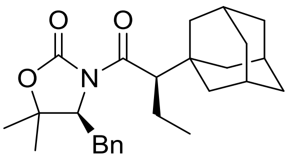

2a

It was prepared following General Procedure 2 from *N*-acyl oxazolidinone **2** (275 mg, 1.0 mmol), $TiCl_4$ (121 μL, 1.1 mmol), Et_3N (700 μL, 5.0 mmol) and *tert*-butyl perester **a** (379 mg, 1.5 mmol) for 3 h. Purification of the residue by flash column chromatography (95:5 hexanes/EtOAc) afforded **2a** (220 mg, 0.54 mmol, 54% yield) as a white solid. **mp** 155–157 °C; $\mathbf{R_f}$ (90:10 hexanes/EtOAc) 0.5; ; $[\alpha]_D^{20}$ –41.0 (*c* 1.0, $CHCl_3$); **IR** (ATR) ν 2964, 2901, 2888, 2846, 1771, 1682, 1347, 1226, 1174 cm^{-1}; **^{1}H NMR** (400 MHz, $CDCl_3$) δ 7.38–7.20 (5H, m), 4.62 (1H, dd, *J* = 10.3, 3.2 Hz), 3.73 (1H, dd, *J* = 11.8, 3.2 Hz), 3.24 (1H, dd, *J* = 14.4, 3.2 Hz), 2.89 (1H, dd, *J* = 14.4, 10.3 Hz), 1.97–1.91 (3H, m), 1.85–1.79 (3H, m), 1.70–1.57 (8H, m), 1.54–1.48 (3H, m), 1.33 (3H, s), 1.32 (3H, s), 0.85 (3H, t, *J* = 7.4 Hz); **^{13}C NMR** (100.6 MHz, $CDCl_3$) δ 176.3 (C), 152.7 (C), 137.3 (C), 129.0 (CH), 128.6 (CH), 126.7 (CH), 81.2 (C), 64.0 (CH), 53.6 (CH), 39.7 (CH_2), 37.0 (CH_2), 36.2 (C), 35.6 (CH_2), 28.6 (CH_3), 28.4 (CH_3), 22.3 (CH_3), 19.7 (CH_2), 12.7 (CH_3); **HRMS** (+ESI) *m/z*: $[M+Na]^+$ Calcd for $C_{26}H_{35}NNaO_3$ 432.2509; Found 432.2512.

(*S*)-*N*-[(*R*)-2-Adamantyl-3-phenylpropanoyl]-4-benzyl-5,5-dimethyl-1,3-oxazolidin-2-one (3a)

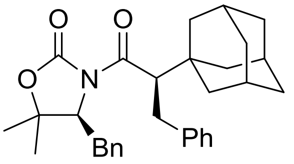

3a

It was prepared following General Procedure 2 from *N*-acyl oxazolidinone **3** (169 mg, 0.5 mmol), $TiCl_4$ (61 μL, 0.55 mmol), Et_3N (350 μL, 2.5 mmol) and *tert*-butyl perester **a** (189 mg, 0.75 mmol) for 2 h. Purification of the residue by flash column chromatography (from 99:1 to 95:5 hexanes/EtOAc) afforded **3a** (103 mg, 0.22 mmol, 44% yield) as a white solid. **mp** 150–155 °C; $\mathbf{R_f}$ (90:10 hexanes/EtOAc) 0.3; $[\alpha]_D^{20}$ –41.4 (*c* 1.0, $CHCl_3$); **IR** (ATR) ν 2922, 2902, 1766, 1687, 1387, 1352, 1244, 1229, 1097 cm^{-1}; **^{1}H NMR** (400 MHz, $CDCl_3$) δ 7.30–7.07 (10H, m), 4.24 (1H, dd, *J* = 10.0, 3.6 Hz), 4.19 (1H, dd, *J* = 11.9, 4.1 Hz), 3.06 (1H, dd, *J* = 14.5, 3.6 Hz), 2.96–2.85 (2H, m), 2.80 (1H, dd, *J* = 14.5, 10.0 Hz), 2.01–1.97 (3H, m), 1.95–1.90 (3H, m), 1.74–1.63 (6H, m), 1.62–1.55 (3H, m), 1.15 (3H, s), 0.57 (3H, s); **^{13}C NMR** (100.6 MHz, $CDCl_3$) δ 174.7 (C), 152.4 (C), 140.1 (C), 137.2 (C), 129.1 (CH), 129.0 (CH), 128.5 (CH), 128.4 (CH), 126.7 (CH), 126.1 (CH), 81.3 (C),

63.5 (CH), 53.7 (CH), 39.7 (CH_2), 36.9 (CH_2), 36.1 (C), 35.7 (CH_2), 32.9 (CH_2), 28.6 (CH), 27.1 (CH_3), 22.0 (CH_3); **HRMS** (+ESI) *m/z*: $[M+H]^+$ Calcd for $C_{31}H_{38}NO_3$ 472.2846; Found 472.2850.

(*S*)-*N*-[(*R*)-2-Adamantyl-5-hexenoyl]-4-benzyl-5,5-dimethyl-1,3-oxazolidin-2-one (4a)

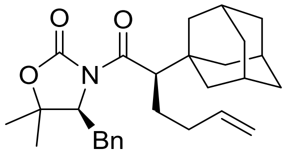

4a

It was prepared following General Procedure 2 from *N*-acyl oxazolidinone **4** (151 mg, 0.5 mmol), $TiCl_4$ (61 µL, 0.55 mmol), Et_3N (350 µL, 2.5 mmol) and *tert*-butyl perester **a** (189 mg, 0.75 mmol) for 2 h. Purification of the residue by flash column chromatography (from 99.5:0.5 to 97.5:2.5 hexanes/EtOAc) afforded **4a** (133 mg, 0.21 mmol, 41% yield) as a colorless oil. $\mathbf{R_f}$ (60:40 hexanes/CH_2Cl_2) 0.4; $[\alpha]_D^{20}$ −28.8 (*c* 1.0, $CHCl_3$); **IR** (ATR) ν 2901, 2847, 1770, 1686, 1348, 1274, 1227, 1205, 1173, 1095 cm^{-1}; **^{1}H NMR** (400 MHz, $CDCl_3$) δ 7.38–7.19 (5H, m), 5.78 (1H, ddt, *J* = 16.8, 10.2, 6.5 Hz), 5.04–4.98 (1H, m), 4.98–4.93 (1H, m), 4.61 (1H, dd, *J* = 10.4, 3.0 Hz), 3.83 (1H, dd, *J* = 11.4, 2.7 Hz), 3.25 (1H, dd, *J* = 14.4, 3.0 Hz), 2.87 (1H, dd, *J* = 14.4, 10.4 Hz), 2.01–1.79 (9H, m), 1.70–1.57 (7H, m), 1.54–1.77 (3H, d, *J* = 12.0 Hz), 1.33 (3H, s), 1.32 (3H, s); **^{13}C NMR** (100.6 MHz, $CDCl_3$) δ 176.0 (C), 152.5 (C), 138.0 (C), 137.2 (CH), 129.0 (CH), 128.6 (CH), 126.7 (CH), 115.0 (CH_2), 81.2 (C), 64.1 (CH), 51.3 (CH), 39.6 (CH_2), 36.9 (CH_2), 36.3 (C), 35.4 (CH_2), 32.5 (CH_2), 28.6 (CH_3), 28.6 (CH), 26.1 (CH_2), 22.4 (CH_3); **HRMS** (+ESI) *m/z*: $[M+H]^+$ Calcd for $C_{28}H_{38}NO_3$ 436.2846; Found 436.2848.

(*S*)-*N*-[(*R*)-2-Adamantyl-5-hexynoyl]-4-benzyl-5,5-dimethyl-1,3-oxazolidin-2-one (5a)

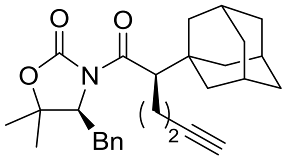

5a

It was prepared following General Procedure 2 from *N*-acyl oxazolidinone **5** (150 mg, 0.5 mmol), $TiCl_4$ (61 µL, 0.55 mmol), Et_3N (350 µL, 2.5 mmol) and *tert*-butyl perester **a** (189 mg, 0.75 mmol) for 2.5 h. Purification of the residue by flash column chromatography (from 99.5:0.5 to 97.5:2.5 hexanes/EtOAc) afforded **5a** (107 mg, 0.25 mmol, 49% yield) as a white solid. **mp** 137–140 °C; $\mathbf{R_f}$ (95:5 hexanes/EtOAc) 0.3; $[\alpha]_D^{20}$ −16.9 (*c* 1.0, $CHCl_3$); **IR** (ATR) ν 3265, 2900, 2888, 2847, 1760, 1694, 1342, 1277, 1203, 1148, 1093 cm^{-1}; **^{1}H NMR** (400 MHz, $CDCl_3$) δ 7.37–7.19 (5H, m), 4.59 (1H, dd, *J* = 10.3, 3.1 Hz), 3.96 (1H, dd, *J* = 11.6, 3.0 Hz), 3.24 (1H, dd, *J* = 14.4, 3.1 Hz), 2.88 (1H, dd, *J* = 14.4, 10.3 Hz), 2.25–2.12 (1H, m), 2.14–2.00 (1H, m), 2.04–1.96 (1H, m), 1.98 (1H, t, *J* = 2.6 Hz), 1.97–1.93 (3H, m), 1.87–1.74 (4H, m), 1.70–1.58 (6H, m), 1.55–1.49 (3H, m), 1.35 (3H, s), 1.32 (3H, s); **^{13}C NMR** (100.6 MHz, $CDCl_3$) δ 175.3 (C), 152.4 (C), 137.2 (C), 129.0

(CH), 128.6 (CH), 126.7 (CH), 83.2 (C), 81.3 (C), 69.5 (CH), 64.2 (CH), 50.6 (CH), 39.5 (CH_2), 36.9 (CH_2), 36.3 (C), 35.5 (CH_2), 28.6 (CH), 28.5 (CH_3), 25.5 (CH_2), 22.4 (CH_3), 17.2 (CH_2); **HRMS** (+ESI) *m/z*: $[M+H]^+$ Calcd for $C_{28}H_{36}NO_3$ 434.2690; Found 434.2688.

(*S*)-*N*-[(*R*)-2-Adamantyl-5-methoxy-5-oxopentanoyl]-4-benzyl-5,5-dimethyl-1,3-oxazolidin-2-one (6a)

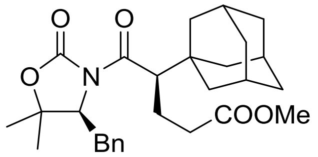

6a

It was prepared following General Procedure 2 from *N*-acyl oxazolidinone **6** (333 mg, 1.0 mmol), $TiCl_4$ (122 µL, 1.1 mmol), Et_3N (700 µL, 5.0 mmol) and *tert*-butyl perester **a** (379 mg, 1.5 mmol) for 12 h. Purification of the residue by flash column chromatography (85:15 hexanes/EtOAc) afforded **6a** (312 mg, 0.67 mmol, 67% yield) as a white solid. **mp** 100–104 °C; $\mathbf{R_f}$ (90:10 hexanes/EtOAc) 0.2; $[\alpha]_D^{20}$ −28.5 (*c* 1.0, $CHCl_3$); **IR** (ATR) ν 2900, 1761, 1734, 1698, 1353, 1272, 1205, 1166, 1105, 1093 cm^{-1}; ^{1}**H NMR** (400 MHz, $CDCl_3$) δ 7.38–7.20 (5H, m), 4.62 (1H, dd, *J* = 10.5, 3.0 Hz), 3.79 (1H, dd, *J* = 11.2, 3.4 Hz), 3.66 (3H, s), 3.25 (1H, dd, *J* = 14.4, 2.9 Hz), 2.87 (1H, dd, *J* = 14.4, 10.5 Hz), 2.32–2.13 (2H, m), 2.07–1.92 (5H, m), 1.84–1.78 (3H, m), 1.70–1.57 (6H, m), 1.55–1.48 (3H, m), 1.37 (3H, s), 1.32 (3H, s); ^{13}**C NMR** (100.6 MHz, $CDCl_3$) δ 175.5 (C), 173.3 (C), 152.5 (C), 137.2 (C), 129.0 (CH), 128.6 (CH), 126.8 (CH), 81.3 (C), 64.0 (CH), 51.6 (CH_3), 50.8 (CH), 39.5 (CH_2), 36.8 (CH_2), 36.4 (C), 35.4 (CH_2), 32.8 (CH_2), 28.6 (CH), 28.4 (CH_3), 22.4 (CH_3), 22.2 (CH_2); **HRMS** (+ESI) *m/z*: $[M+H]^+$ Calcd for $C_{28}H_{38}NO_5$ 468.2744; Found 468.2745.

(*S*)-*N*-[(*R*)-2-Adamantyl-2-phenylacetyl]-4-benzyl-5,5-dimethyl-1,3-oxazolidin-2-one (7a)

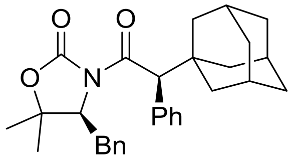

7a

It was prepared following General Procedure 2 from *N*-acyl oxazolidinone **7** (323 mg, 1.0 mmol), $TiCl_4$ (122 µL, 1.1 mmol), Et_3N (700 µL, 5.0 mmol) and *tert*-butyl perester **a** (379 mg, 1.5 mmol) for 3 h. Purification of the residue by flash column chromatography (95:5 hexanes/EtOAc) afforded **7a** (289 mg, 0.64 mmol, 64% yield) as a white solid. **mp** 206–208 °C; $\mathbf{R_f}$ (90:10 hexanes/EtOAc) 0.4; $[\alpha]_D^{20}$ +47.2 (*c* 1.0, $CHCl_3$), [lit.[4] $[\alpha]_D^{20}$ +33.8 (*c* 1.0, $CHCl_3$)]; **IR** (ATR) ν 2903, 1760, 1699, 1348, 1293, 1279, 1213, 1165, 1093 cm^{-1}; ^{1}**H NMR** (400 MHz, $CDCl_3$) δ 7.40–7.20 (10H, m), 4.94 (1H, s), 4.48 (1H, dd, *J* = 9.7, 3.9 Hz), 3.24 (1H, dd, *J* = 14.3, 3.9 Hz), 2.90 (1H, dd, *J* = 14.3, 9.7 Hz), 1.95–1.90 (3H, m), 1.81–1.74 (3H, m), 1.66–1.52 (9H, m), 1.28 (3H, s), 1.01 (3H, s); ^{13}**C NMR** (100.6 MHz, $CDCl_3$) δ 173.0 (C), 152.5 (C), 137.0 (C), 134.4 (C), 130.9 (CH),

129.1 (CH), 128.6 (CH), 127.6 (CH), 127.1 (CH), 126.7 (CH), 81.5 (C), 63.8 (CH), 58.4 (CH), 39.9 (CH_2), 37.2 (C), 36.8 (CH_2), 35.6 (CH_2), 28.6 (CH), 28.1 (CH_3), 22.2 (CH_3); **HRMS** (+ESI) *m/z*: $[M+H]^+$ Calcd for $C_{30}H_{36}NO_3$ 458.2690; Found 458.2704. Data are consistent with a reported example.[4]

(*S*)-*N*-[(*R*)-2-Adamantyl-2-*tert*-butyldimethylsilyloxyacetyl]-4-benzyl-5,5-dimethyl-1,3-oxazolidin-2-one (8a)

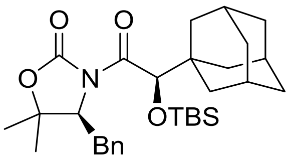

8a

It was prepared following General Procedure 2 from *N*-acyl oxazolidinone **8** (2.45 g, 6.5 mmol), $TiCl_4$ (790 µL, 7.1 mmol), Et_3N (4.5 mL, 32 mmol) and *tert*-butyl perester **a** (2.46 g, 9.8 mmol) for 3 h. Purification of the residue by flash column chromatography (from 99:1 to 95:5 hexanes/EtOAc) afforded **8a** (2.32 mg, 4.5 mmol, 69% yield) as a white solid. **mp** 60–64 °C; **R$_f$** (90:10 hexanes/EtOAc) 0.5; $[\alpha]_D^{20}$ −35.6 (*c* 1.0, $CHCl_3$); **IR** (ATR) ν 2902, 2850, 1771, 1703, 1349, 1252, 1129, 1099 cm^{-1}; **^{1}H NMR** (400 MHz, $CDCl_3$) δ 7.38–7.20 (5H, m), 5.37 (1H, s), 4.65 (1H, dd, *J* = 10.8, 2.9 Hz), 3.25 (1H, dd, *J* = 14.3, 2.6 Hz), 2.83 (1H, dd, *J* = 14.3, 10.8 Hz), 1.99–1.93 (3H, m), 1.81–1.75 (3H, m), 1.70–1.54 (9H, m), 1.33 (3H, s), 1.31 (3H, s), 0.93 (9H, s), 0.08 (3H, s), 0.01 (3H, s); **^{13}C NMR** (100.6 MHz, $CDCl_3$) δ 173.1 (C), 152.5 (C), 136.9 (C), 129.0 (CH), 128.7 (CH), 126.8 (CH), 81.9 (C), 75.4 (CH), 63.9 (CH), 38.4 (C), 37.7 (CH_2), 37.0 (CH_2), 35.3 (CH_2), 28.9 (CH_3), 28.3 (CH), 25.8 (CH_3), 22.6 (CH_3), 18.2 (C), −4.8 (CH_3), −5.2 (CH_3); **HRMS** (+ESI) *m/z*: $[M+H]^+$ Calcd for $C_{30}H_{46}NO_4Si$ 512.3191; Found 512.3191.

(*S*)-*N*-[(*R*)-2-Adamantyl-2-(1-*H*-*N*-pyrrolyl)acetyl]-4-benzyl-5,5-dimethyl-1,3-oxazolidin-2-one (9a)

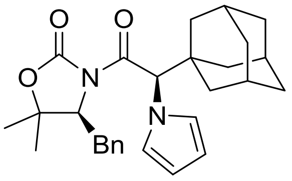

9a

It was prepared following General Procedure 2 from *N*-acyl oxazolidinone **9** (2.00 g, 6.4 mmol), $TiCl_4$ (770 µL, 7.0 mmol), Et_3N (4.5 mL, 32 mmol) (at −20 °C) and *tert*-butyl perester **a** (2.42 g, 9.6 mmol) for 3 h at rt. Purification of the residue by flash column chromatography (from 95:5 to 90:10 hexanes/EtOAc) afforded **9a** (1.77 g, 4.0 mmol, 62% yield) as a white solid. **mp** 210–212 °C; **R$_f$** (90:10 hexanes/EtOAc) 0.4; $[\alpha]_D^{20}$ −22.2 (*c* 1.0, $CHCl_3$); **IR** (ATR) ν 2904, 2846, 1760, 1699, 1348, 1293, 1213, 1166, 1094 cm^{-1}; **^{1}H NMR** (400 MHz, $CDCl_3$) δ 7.37–7.14 (5H, m), 6.88 (2H, t, *J* = 2.2 Hz), 6.12 (2H, m, *J* = 2.2 Hz), 5.99 (1H, s), 4.53 (1H, dd, *J* = 10.0, 3.8 Hz), 3.28 (1H, dd, *J* = 14.3, 3.8 Hz), 2.89 (1H, dd, *J* = 14.3, 10.0 Hz), 2.00–1.93 (3H, m), 1.74–

1.56 (12H, m), 1.34 (3H, s), 1.18 (3H, s); **^{13}C NMR** (100.6 MHz, $CDCl_3$) δ 169.4 (C), 152.4 (C), 136.6 (C), 129.0 (CH), 128.7 (CH), 126.9 (CH), 122.7 (CH), 107.4 (CH), 82.1 (C), 66.3 (CH), 63.9 (CH), 38.8 (CH_2), 38.3 (C), 36.6 (CH_2), 35.2 (CH_2), 28.3 (CH_3), 28.3 (CH), 22.4 (CH_3); **HRMS** (+ESI) *m/z*: $[M+H]^+$ Calcd for $C_{28}H_{35}N_2O_3$ 447.2642; Found 447.2646.

5 Synthesis of Enantiomerically Pure Derivatives (Scheme 5)

(*R*)-2-Adamantyl-1-propanol (10)

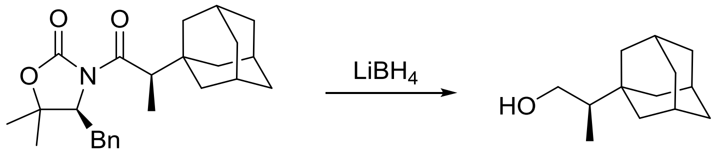

A 2 M solution of $LiBH_4$ in THF (600 µL, 1.2 mmol) was added to a solution of **1a** (158 mg, 0.40 mmol) in THF (3 mL) and H_2O (1 mL) at 0 °C. The reaction mixture was stirred for 3 h at rt. Two batches of $LiBH_4$ 2 M in THF (300 µL, 0.60 mmol) were added after 2.5 h and then 1.5 h. The reaction mixture was quenched with MeOH (500 uL) and partitioned between EtOAc and H_2O (6 mL each). The aqueous layer was extracted with further EtOAc (2 × 6 mL). The combined organic extracts were dried with anhydrous $MgSO_4$ and the volatiles evaporated. Purification of the residue by flash column chromatography (from 90:10 to 85:15 hexanes:EtOAc) afforded **SI1** (50 mg, 0.26 mmol, 64% yield) and **10** (50 mg, 0.26 mmol, 64% yield) as a white solid. **mp** 74–75 °C; **R$_f$** (80:20 hexanes/EtOAc) 0.4; $[\alpha]_D^{20}$ −22.6 (*c* 1.0, $CHCl_3$); **IR** (ATR) br 3304, 2895, 2846, 1447, 1361, 1344, 1030, 1015 cm^{-1}; **^{1}H NMR** (400 MHz, $CDCl_3$) δ 3.84 (1H, dd, *J* = 10.4, 3.9 Hz), 3.36 (1H, dd, *J* = 10.4, 8.4 Hz), 1.98–1.93 (3H, m), 1.73–1.60 (6H, m), 1.60–1.48 (6H, m), 1.28–1.18 (2H, m), 0.92 (3H, d, *J* = 7.0 Hz); **^{13}C NMR** (100.6 MHz, $CDCl_3$) δ 64.4 (CH_2), 46.0 (CH), 39.9 (CH_2), 37.3 (CH_2), 34.1 (C), 28.7 (CH), 10.9 (CH_3); **HRMS** (−ESI) *m/z*: $[M-H]^-$ Calcd for $C_{13}H_{21}O$ 193.1598; Found 193.1600. Data is consistent with a reported example.[5]

(*R*)-1-Adamantyl-1,2-etandiol (11)

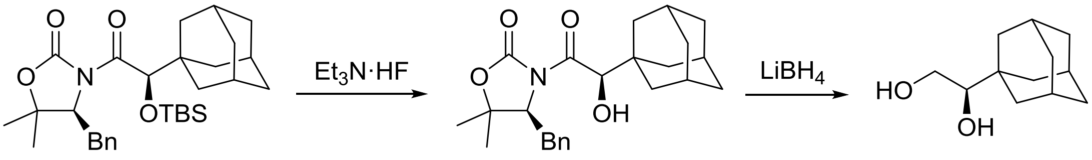

Neat $Et_3N{\cdot}3HF$ (650 µL, 4.0 mmol) was added to a solution of **8a** (256 mg, 0.50 mmol) in ACN (4 mL). The reaction mixture was stirred at 40 °C for 18 h and allowed to cool to rt. The mixture was partitioned between EtOAc and H_2O (7 mL each). The aqueous layer was extracted with further EtOAc (2 × 7 mL). The combined organic extracts were dried with anhydrous $MgSO_4$ and the volatiles evaporated. Purification of the residue by flash column chromatography (from 99:1 to 90:10 hexanes:EtOAc) afforded **SI3** (124 mg, 0.31 mmol, 62% yield) as a caramel white solid. **R$_f$** (80:20 hexanes/EtOAc) 0.4; $[\alpha]_D^{20}$ −61.6 (*c* 1.0, $CHCl_3$); **IR** (ATR) ν br 3500, 2903, 2849, 1776, 1731, 1684, 1353, 1275, 1232, 1209, 1102 cm^{-1}; **^{1}H NMR** (400 MHz, $CDCl_3$) δ 7.37–7.22 (5H, m), 5.11 (1H, d, *J* = 10.0 Hz), 4.65 (1H, dd, *J* = 10.2, 3.7 Hz), 3.26 (1H, dd, *J* = 14.3, 3.7 Hz), 2.93 (1H, d, *J* = 10.5 Hz), 2.87 (1H, dd, *J* = 14.3, 10.2 Hz), 2.03–1.95 (3H, m), 1.80–1.75 (3H, m), 1.73–1.60 (6H,

m), 1.56 (3H, m), 1.38 (3H, s), 1.33 (3H, s); **^{13}C NMR** (100.6 MHz, $CDCl_3$) δ 174.9 (C), 152.1 (C), 136.5 (C), 129.0 (CH), 128.8 (CH), 127.0 (CH), 82.5 (C), 76.2 (CH), 63.9 (CH), 38.3 (C), 37.5 (CH_2), 36.9 (CH_2), 35.3 (CH_2), 28.8 (CH_3), 28.3 (CH), 22.7 (CH_3); **HRMS** (+ESI) *m/z*: $[M+H]^+$ Calcd for $C_{24}H_{32}NO_4$ 398.2326; Found 398.2329.

A 2M solution of $LiBH_4$ in THF (450 μL, 0.90 mmol) was added to a solution of **SI3** (115 mg, 0.29 mmol) in THF (2.4 mL) and H_2O (0.6 mL) at 0 °C. The reaction mixture was stirred for 1 h at 0 °C. The reaction mixture was quenched with MeOH (400 uL) and partitioned between EtOAc and H_2O (6 mL each). The aqueous layer was extracted with further EtOAc (2 × 6 mL). The combined organic extracts were dried with anhydrous $MgSO_4$ and the volatiles evaporated. Purification of the residue by flash column chromatography (from 99.75:0.25 to 98:2 CH_2Cl_2/MeOH) afforded **SI1** (59 mg, 0.29 mmol, 97% yield) and **11** (44 mg, 0.22 mmol, 77% yield; 48% over two steps) as a white solid. **mp** 113–114 °C; **R$_f$** (98:2 CH_2Cl_2/MeOH) 0.3; $[\alpha]_D^{20}$ −10.7 (*c* 1.0, $CHCl_3$); **IR** (ATR) ν br 3270, 2899, 2848, 1449, 1343, 1077, 1051, 1028 cm^{-1}; **^{1}H NMR** (400 MHz, $CDCl_3$) δ 3.74 (1H, dd, *J* = 11.0, 2.9 Hz), 3.55 (1H, dd, *J* = 11.0, 9.4 Hz), 3.22 (1H, dd, *J* = 9.4, 2.9 Hz), 2.47 (2H, br s), 2.03–1.92 (3H, m), 1.78–1.49 (12H, m); **^{13}C NMR** (100.6 MHz, $CDCl_3$) δ 80.0 (CH), 62.2 (CH_2), 38.2 (CH_2), 37.1 (CH_2), 35.6 (C), 28.2 (CH); **HRMS** (+ESI) *m/z*: $[M-H]^-$ Calcd for $C_{12}H_{19}O_2$ 195.1391; Found 195.1393.

(*R*)-2-Adamantyl-2-aminoetanol

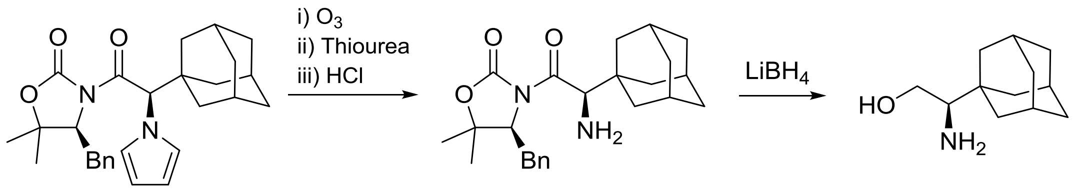

9a **SI4** **12**

Ozone was bubbled through a solution of **9a** (179 mg, 0.4 mmol) in methanol (18 mL) and CH_2Cl_2 (9 mL) at −78 °C for 50 min. Nitrogen was then bubbled through the mixture at −78 °C for 15 min and then a solution of thiourea (64 mg, 0.8 mmol) in methanol (3 mL) was added via *cannula*. The resultant mixture was stirred at −78 °C for 15 min, allowed to warm to rt and stirred for 45 min. Then, the mixture was filtered through a short pad of Celite® and concentrated under reduced pressure. The residue was redissolved in methanol (6 mL) and 4 M HCl in dioxane (5.5 mL, 32 mmol) was added. The mixture was stirred at rt for 16 h and concentrated under reduced pressure (2 M NaOH trap was used). The residue was partitioned between EtOAc and H_2O (10 mL each). The aqueous layer was extracted with further EtOAc (10 mL). The combined organic extracts were washed with brine and dried with anhydrous $MgSO_4$ and the volatiles evaporated. The resultant amine **SI4** was used in the next step without further purification. **^{1}H NMR** (400 MHz, $CDCl_3$) δ 8.46 (3H, br s), 7.50–7.12 (5H, m), 5.17 (1H, s), 4.84 (1H, d, *J* = 9.4 Hz), 3.20 (1H, d, *J* = 14.1 Hz), 2.93–2.87 (1H, m), 2.14–1.86 (6H, m), 1.68 (9H, s), 1.45 (3H, s), 1.31 (3H, s).

A 2 M solution of $LiBH_4$ in THF (600 μL, 1.2 mmol) was added to a solution of non-pure **SI4** (247 mg, ≈0.5 mmol) in THF (3.2 mL) and H_2O (0.8 mL) at 0 °C and the resultant mixture was stirred for 45 min at rt. The

reaction mixture was quenched with MeOH (500 uL) and partitioned between CH_2Cl_2 and H_2O (10 mL each). The aqueous layer was extracted with further CH_2Cl_2 (2 × 10 mL). The combined organic extracts were dried with anhydrous $MgSO_4$ and the volatiles evaporated. Purification of the residue by flash column chromatography (from 95:5 to 70:30 CH_2Cl_2/MeOH) afforded **12** (33 mg, 0.17 mmol, 42% yield over two steps) as a white-off solid. **mp** 94–99 °C; **R_f** (9:1 CH_2Cl_2/MeOH) 0.2; $[\alpha]_D^{20}$ −17.2 (*c* 1.0, $CHCl_3$), [lit.[6] enant. $[\alpha]_D^{20}$ +17.6 (*c* 1.0, $CHCl_3$)]; **IR** (ATR) ν br 3343, 2901, 2848, 1593, 1449, 1057 cm^{-1}; **^{1}H NMR** (400 MHz, $CDCl_3$) δ 3.72 (1H, dd, *J* = 10.3, 3.9 Hz), 3.29 (1H, t, *J* = 10.3 Hz), 2.46–2.26 (4H, m), 2.03–1.95 (3H, m), 1.76–1.69 (3H, m), 1.69–1.59 (3H, m), 1.60–1.49 (6H, m); **^{13}C NMR** (100.6 MHz, $CDCl_3$) δ 62.2 (CH), 61.0 (CH_2), 38.6 (CH_2), 37.1 (CH_2), 35.1 (C), 28.3 (CH); **HRMS** (+ESI) *m/z*: $[M+H]^+$ Calcd for $C_{12}H_{22}NO$ 196.1696; Found 196.1699. Data is consistent with a reported example.[6,7]

6 X-Ray Analysis

Adduct **1c** was dissolved in a vial with the minimum amount of 1:1 CH_2Cl_2/hexanes (1:1). The vial was opened and crystals were allowed to grow at rt for several days. Biggest and clearest crystals were washed with hexanes, dried, and submitted to X-ray analysis described as follows.

A colorless prism-like specimen of $C_{19}H_{27}NO_3$, approximate dimensions 0.100 mm × 0.150 mm × 0.250 mm, was used for the X-ray crystallographic analysis.
The X-ray intensity data were measured on a D8 Venture system equipped with a multilayer monochromator and a Mo microfocus (λ = 1.54178 Å).
The frames were integrated with the Bruker SAINT software package using a narrow-frame algorithm. The integration of the data using an orthorhombic unit cell yielded a total of 16119 reflections to a maximum θ angle of 72.09° (0.81 Å resolution), of which 3420 were independent (average redundancy 4.713, completeness = 95.9%, R_{int} = 2.11%, R_{sig} = 1.67%) and 3409 (99.68%) were greater than 2σ(F^2). The final cell constants of a = 10.1101(2) Å, b = 10.3135(2) Å, c = 17.3285(4) Å, volume = 1806.85(7) $Å^3$, are based upon the refinement of the XYZ-centroids of reflections above 20 σ(I). Data were corrected for absorption effects using the Multi-Scan method (SADABS). The calculated minimum and maximum transmission coefficients (based on crystal size) are 0.6137 and 0.7536.

The structure was solved and refined using the Bruker SHELXTL Software Package, using the space group P 21 21 21, with Z = 4 for the formula unit, $C_{19}H_{27}NO_3$. The final anisotropic full-matrix least-squares refinement on F^2 with 214 variables converged at R1 = 2.57%, for the observed data and wR2 = 6.77% for all data. The goodness-of-fit was 1.064. The largest peak in the final difference electron density synthesis was 0.201 $e^-/Å^3$ and the largest hole was -0.117 $e^-/Å^3$ with an RMS deviation of 0.025 $e^-/Å^3$. On the basis of the final model, the calculated density was 1.167 g/cm^3 and F(000), 688 e^-.

Thermal ellipsoid plot set at 50% probability for 1c

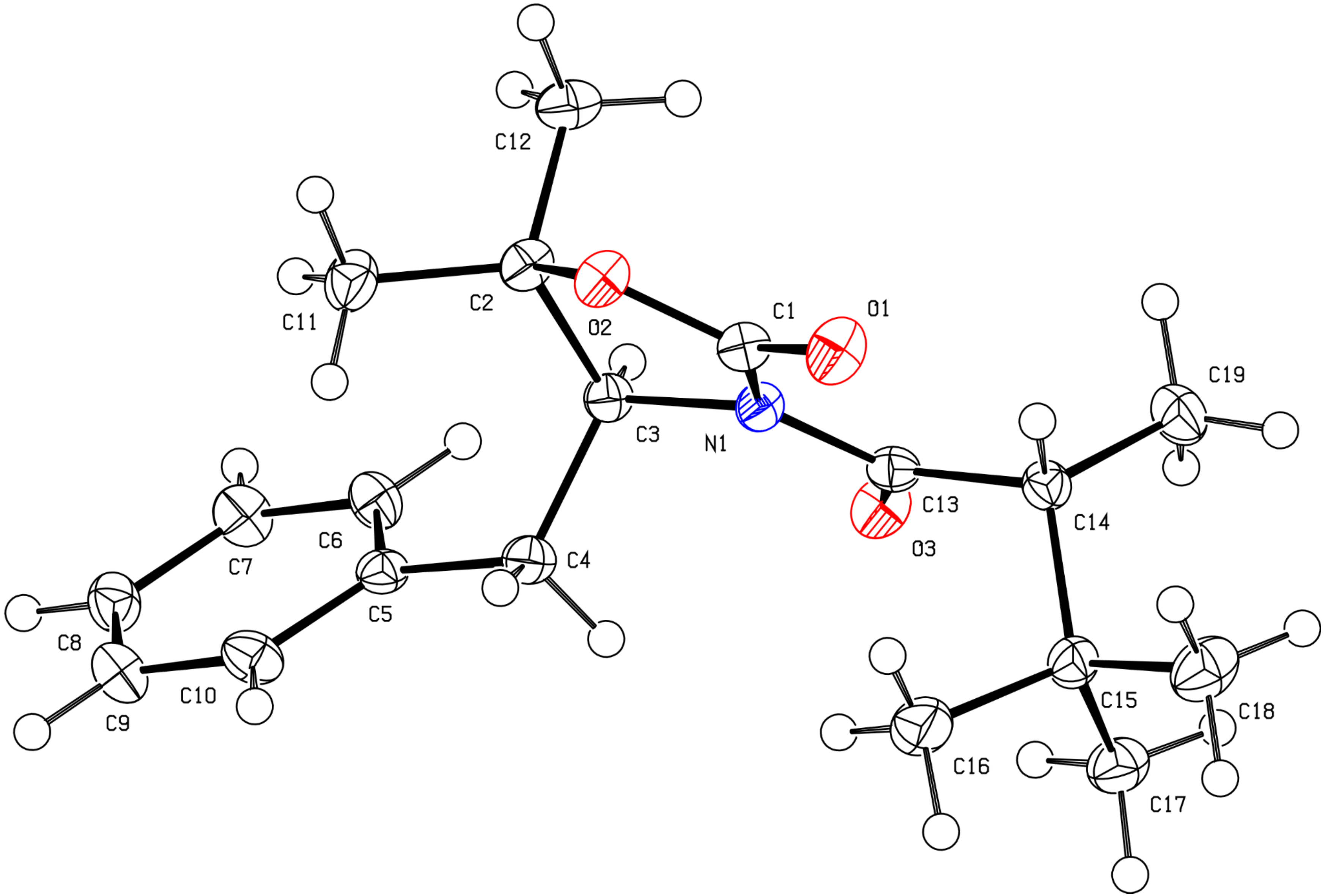

7 ^{1}H NMR and ^{13}C NMR Spectra

^{1}H NMR (400 MHz, $CDCl_3$)

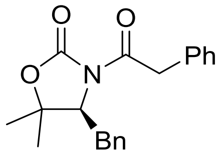

7

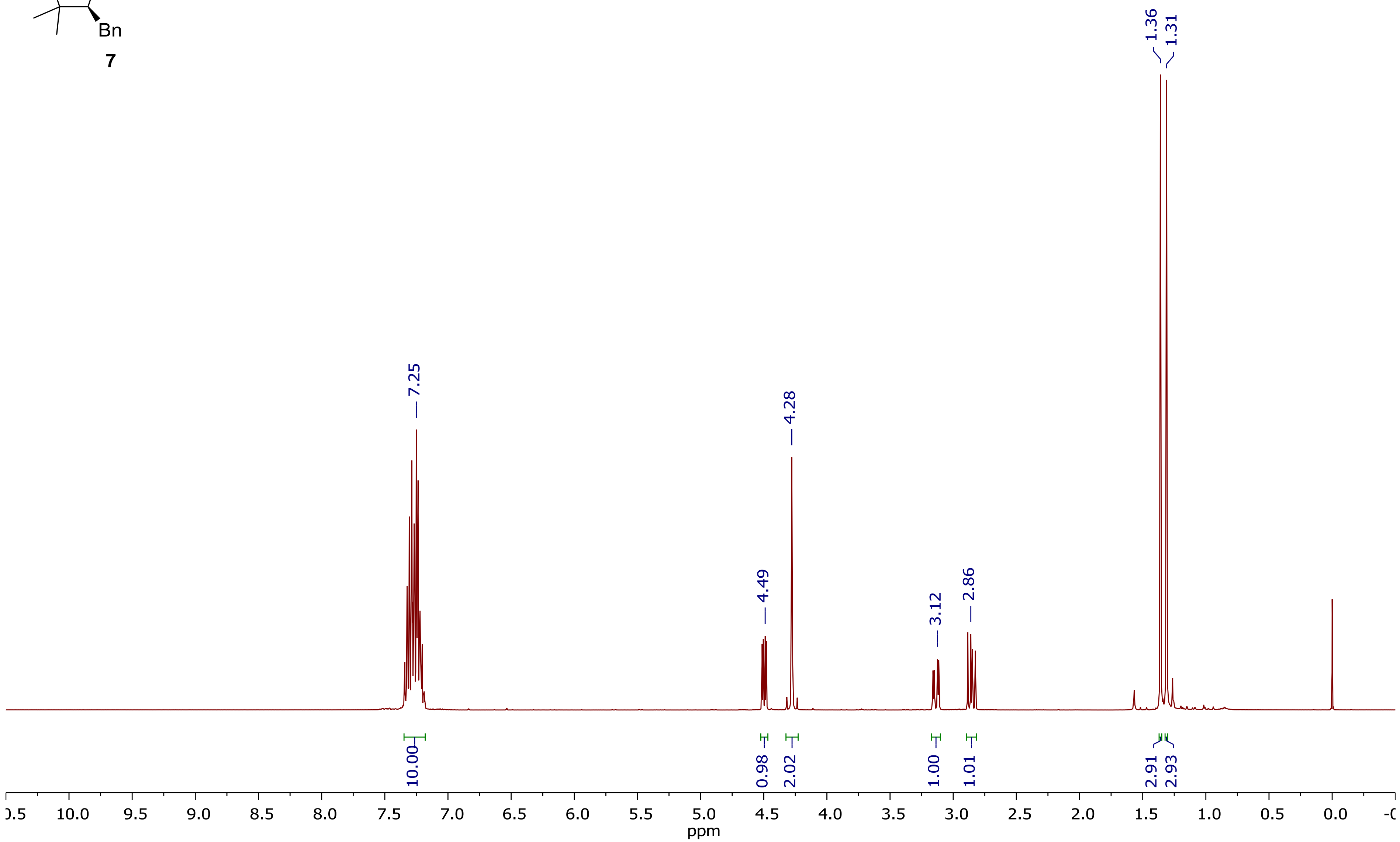

$^{13}C\{^{1}H\}$ NMR (100.6 MHz, $CDCl_3$)

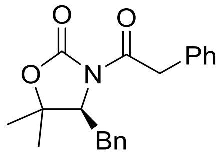

7

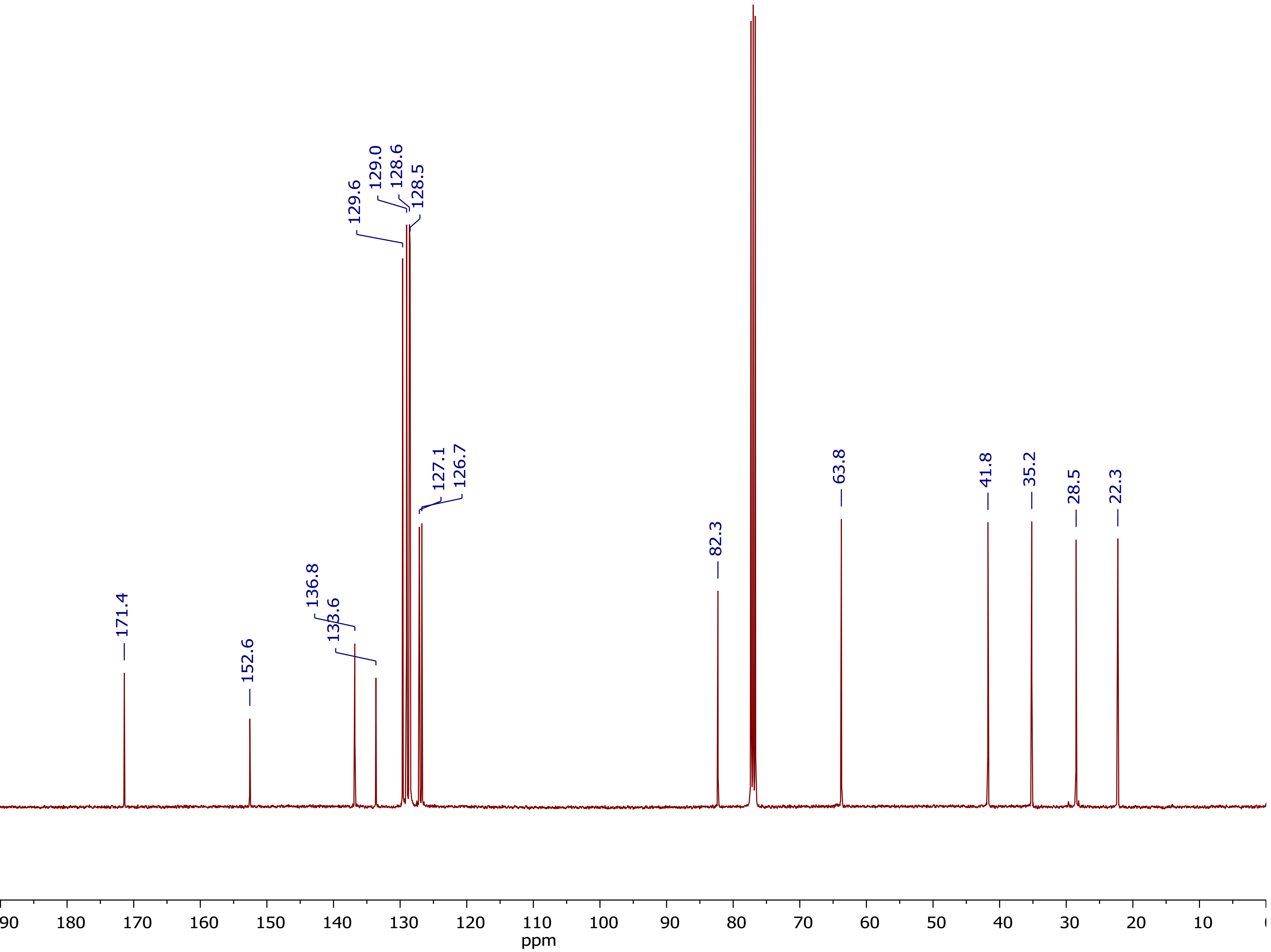

^{1}H NMR (400 MHz, $CDCl_3$)

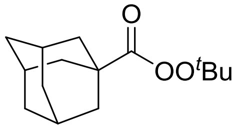

a

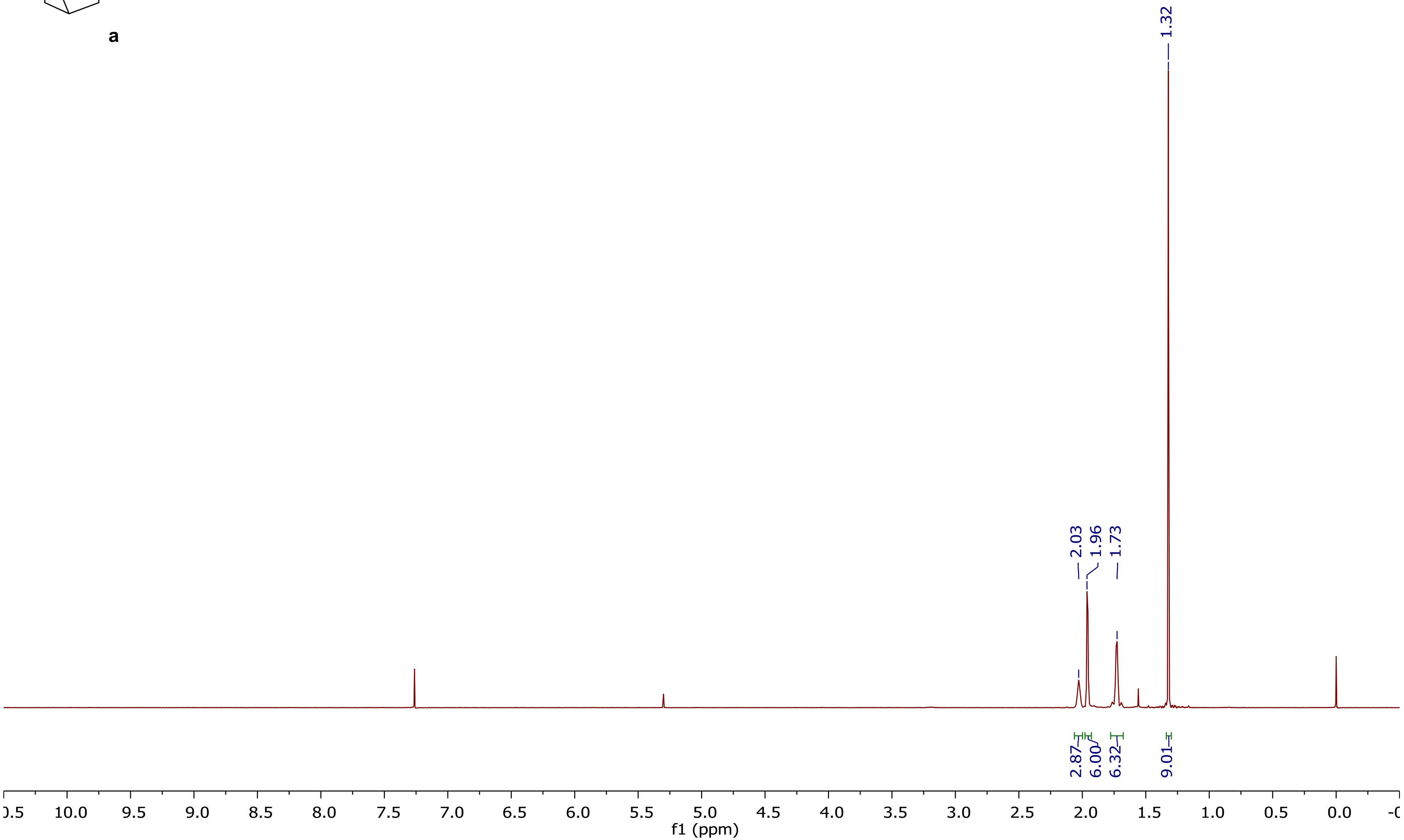

$^{13}C\{^{1}H\}$ NMR (100.6 MHz, $CDCl_3$)

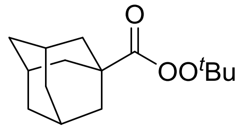

a

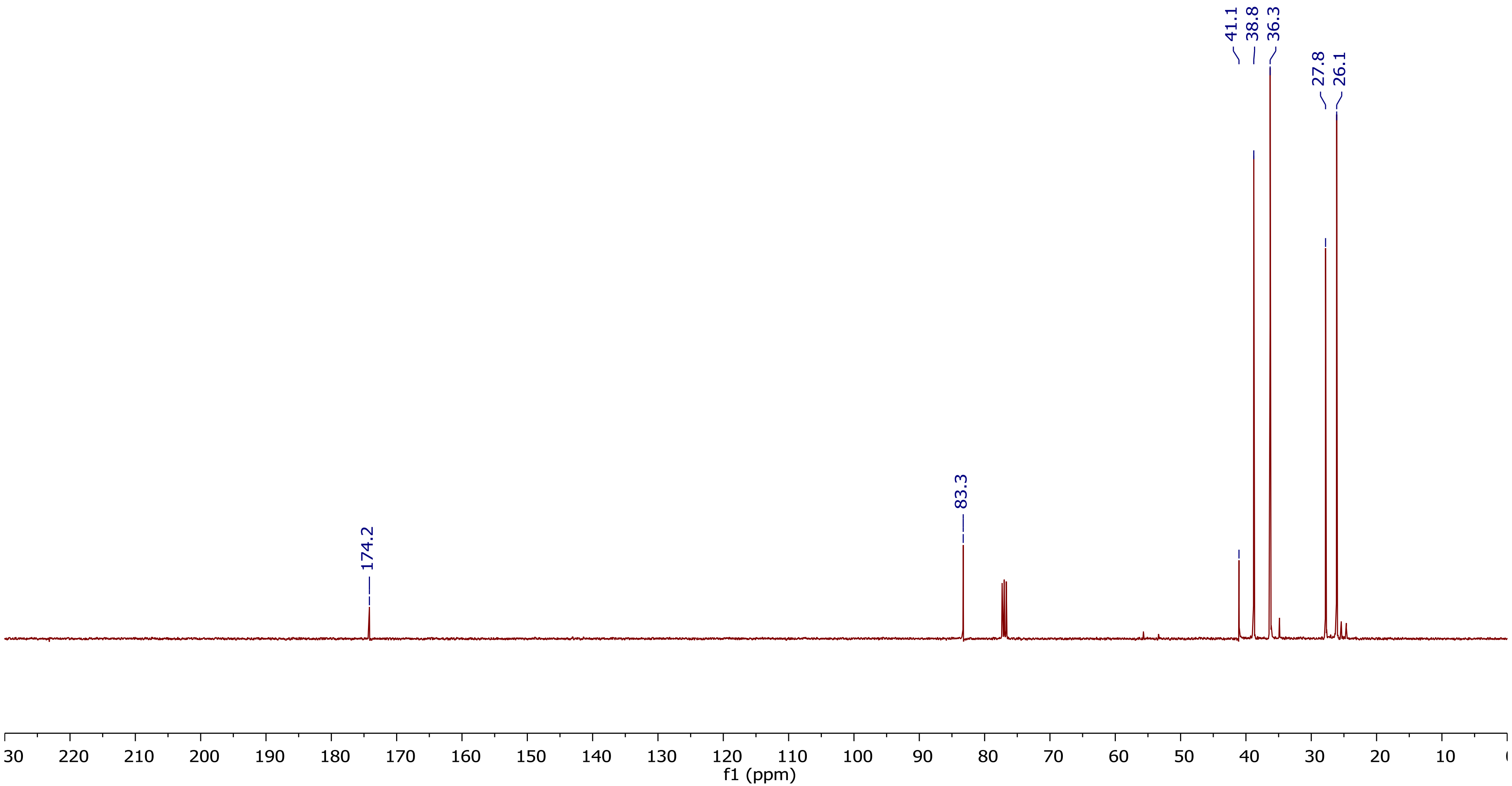

^{1}H NMR (400 MHz, $CDCl_3$)

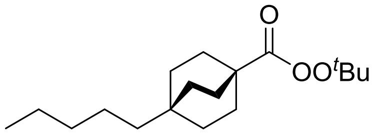

b

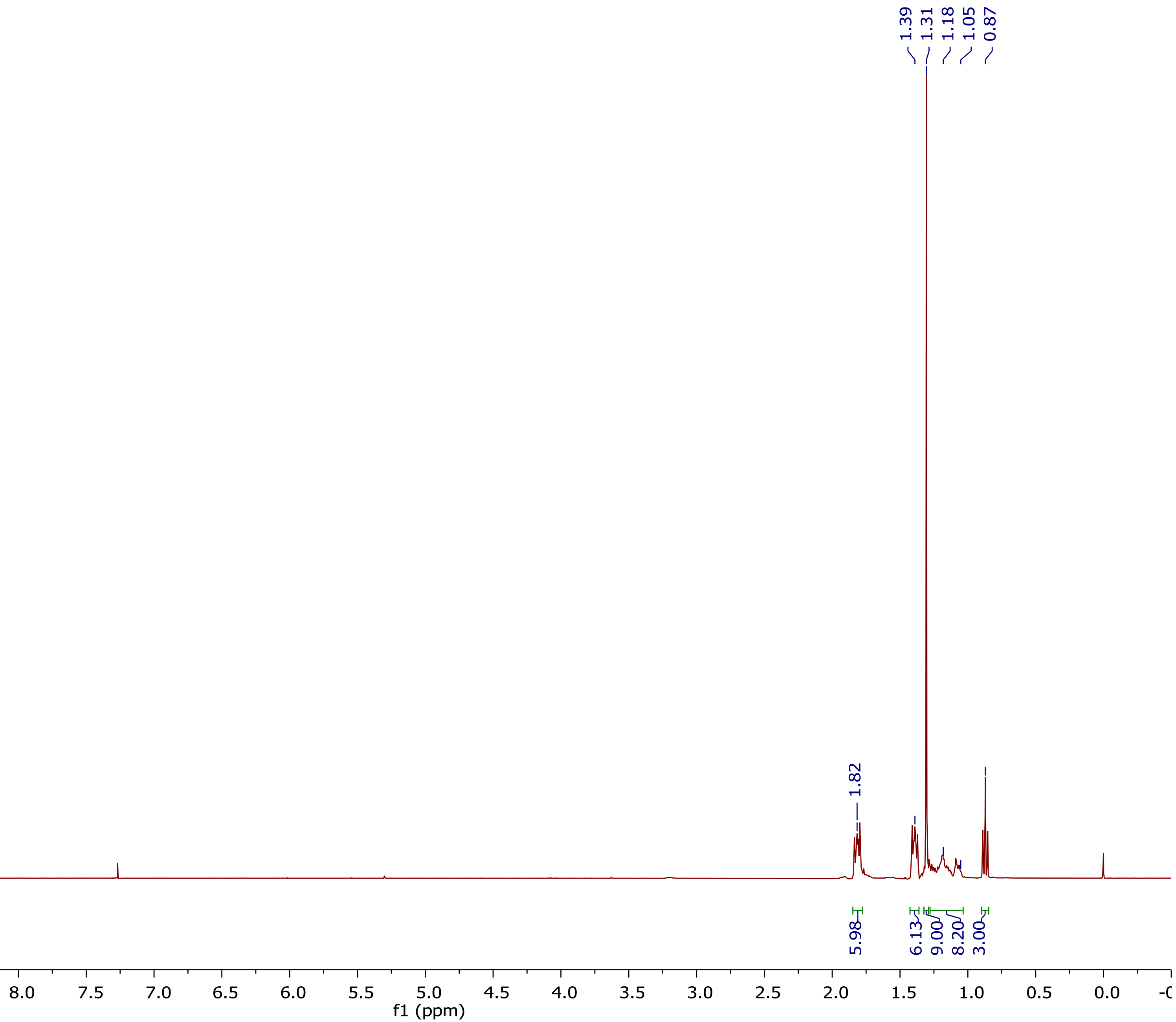

$^{13}C\{^{1}H\}$ NMR (100.6 MHz, $CDCl_3$)

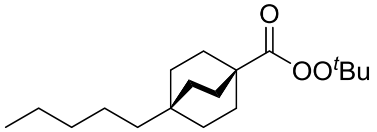

b

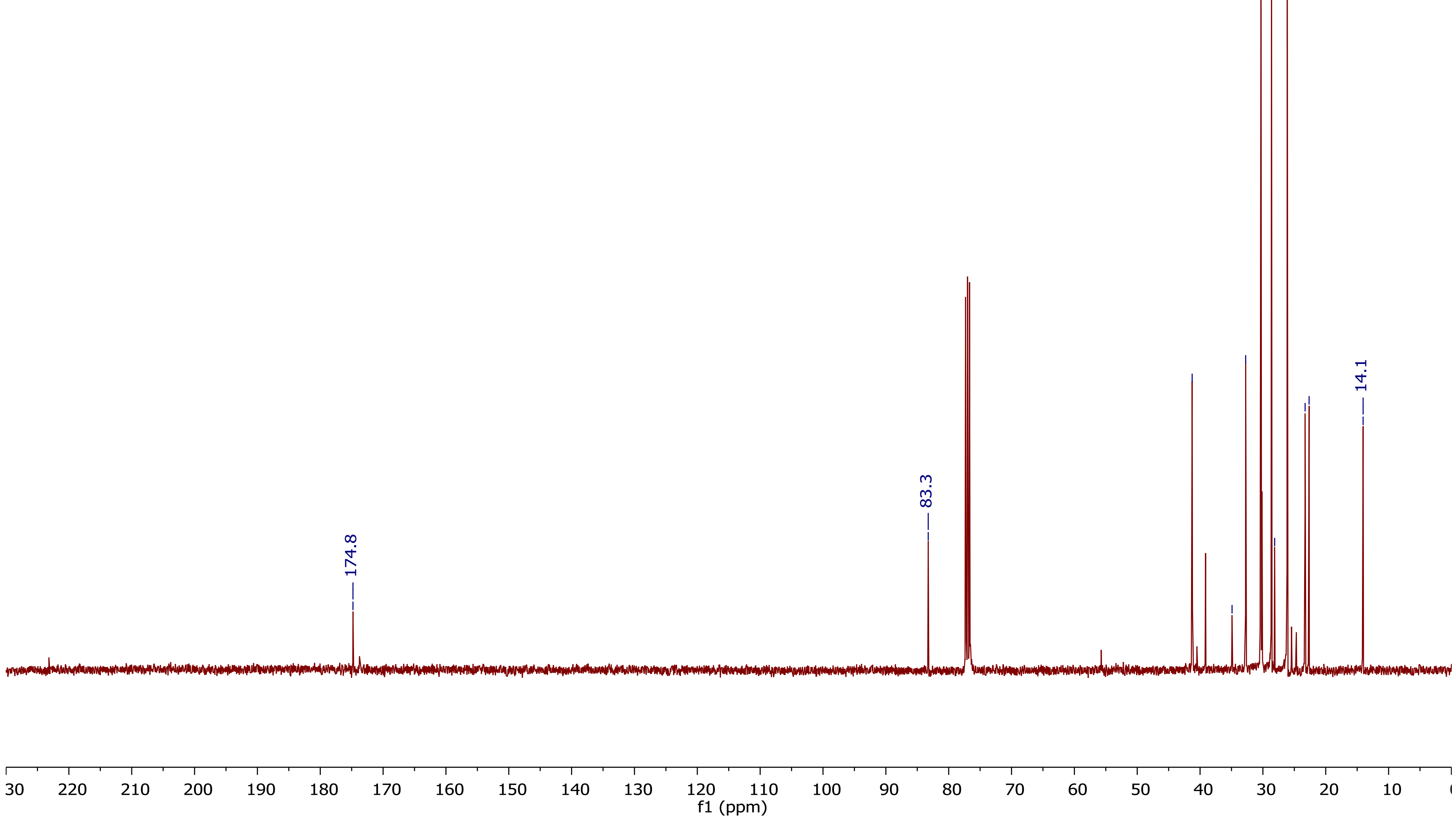

^{1}H NMR (400 MHz, $CDCl_3$)

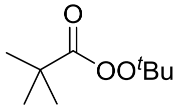

c

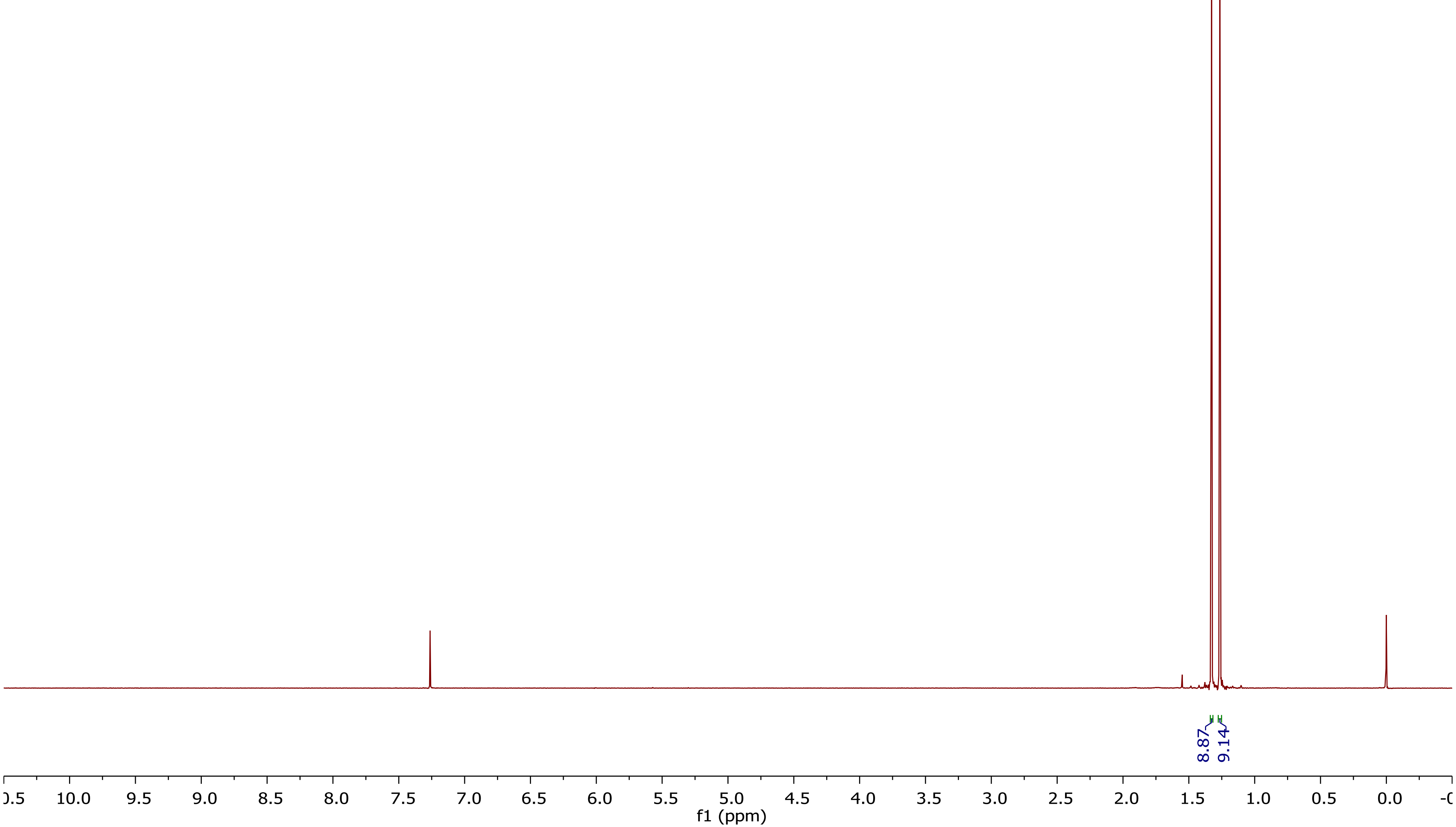

$^{13}C\{^{1}H\}$ NMR (100.6 MHz, $CDCl_3$)

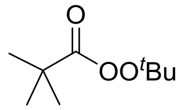

c

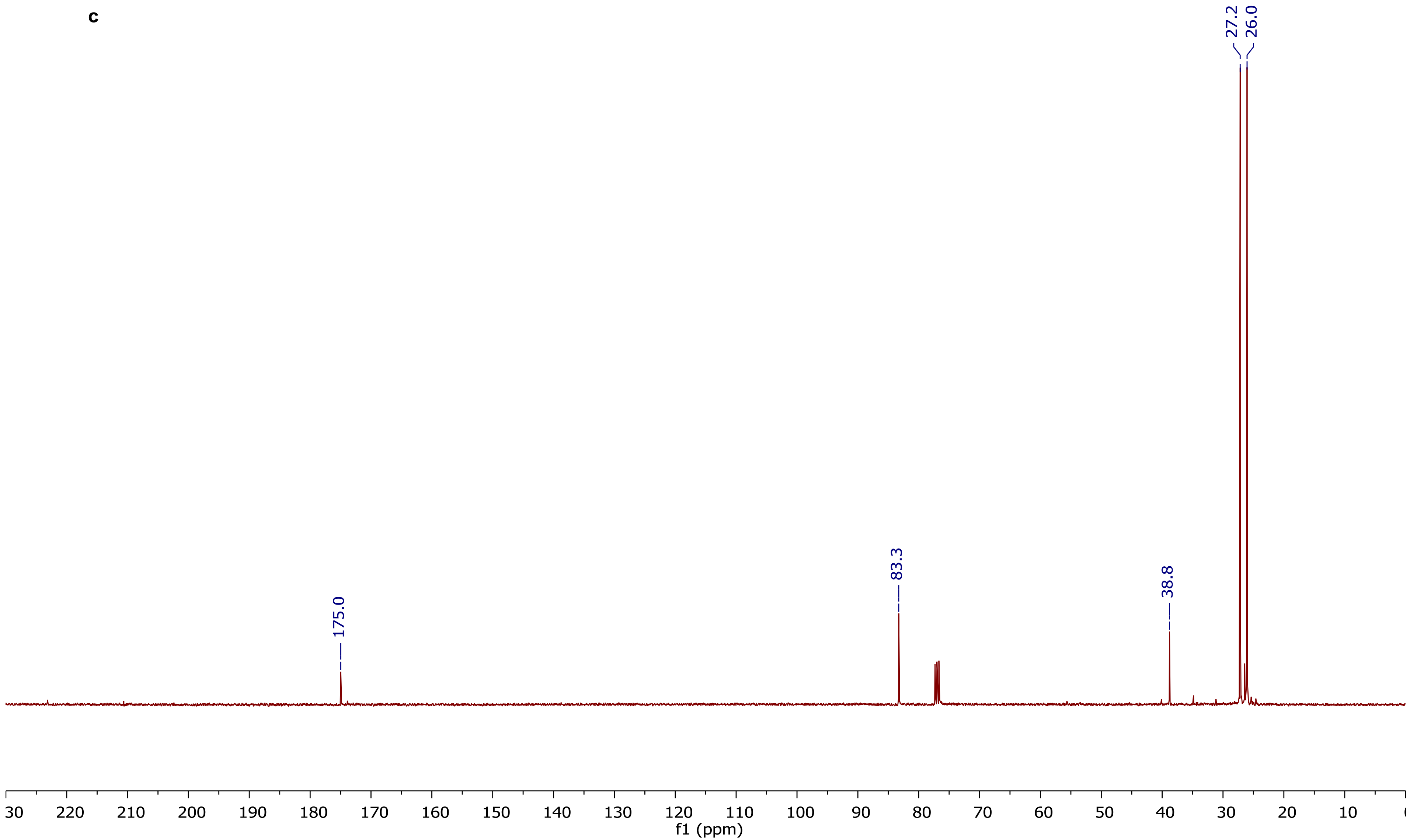

^{1}H NMR (400 MHz, $CDCl_3$)

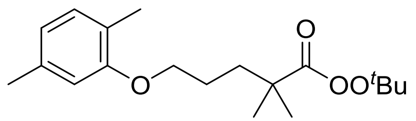

d

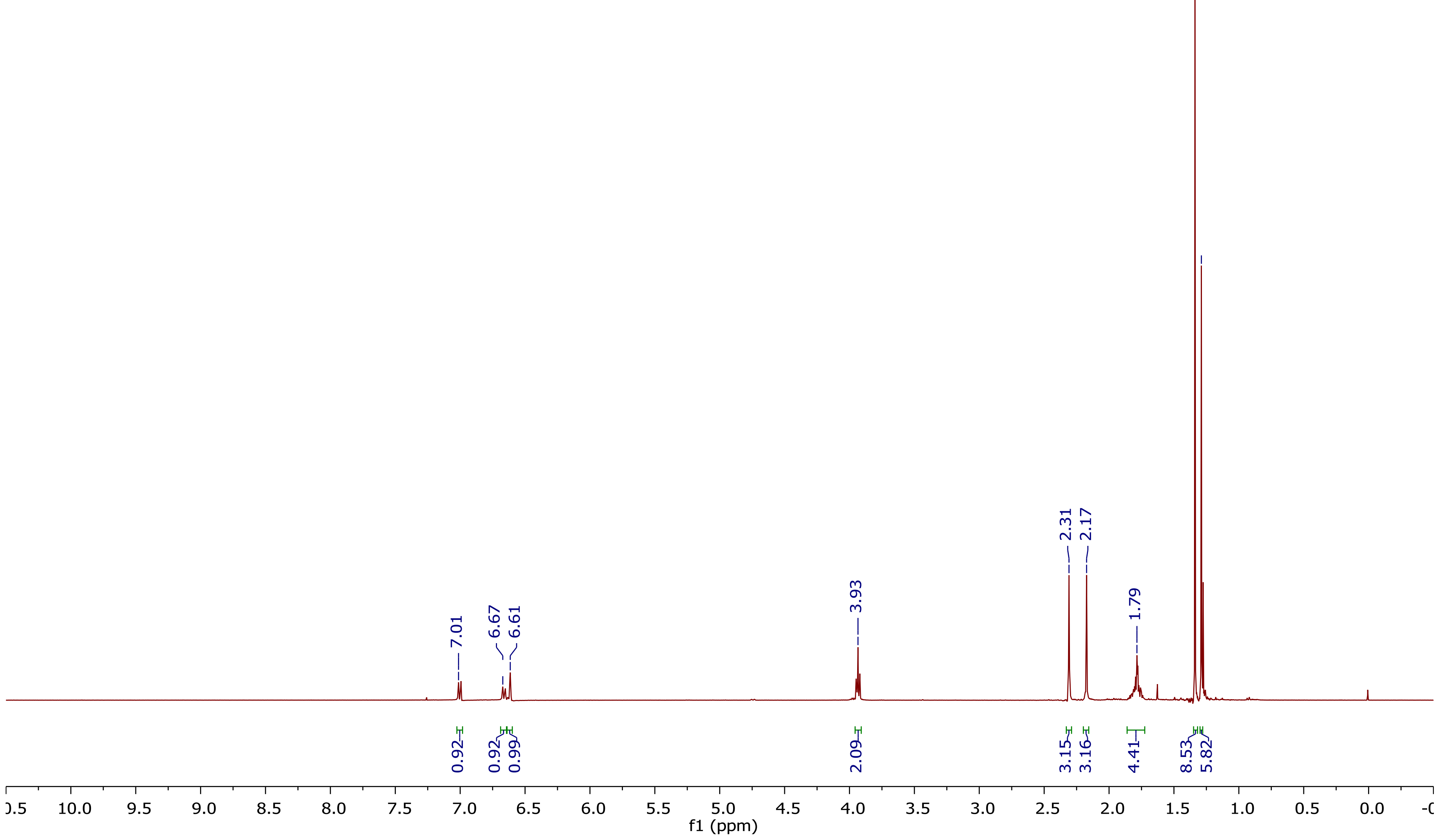

$^{13}C\{^{1}H\}$ NMR (100.6 MHz, $CDCl_3$)

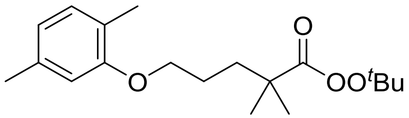

d

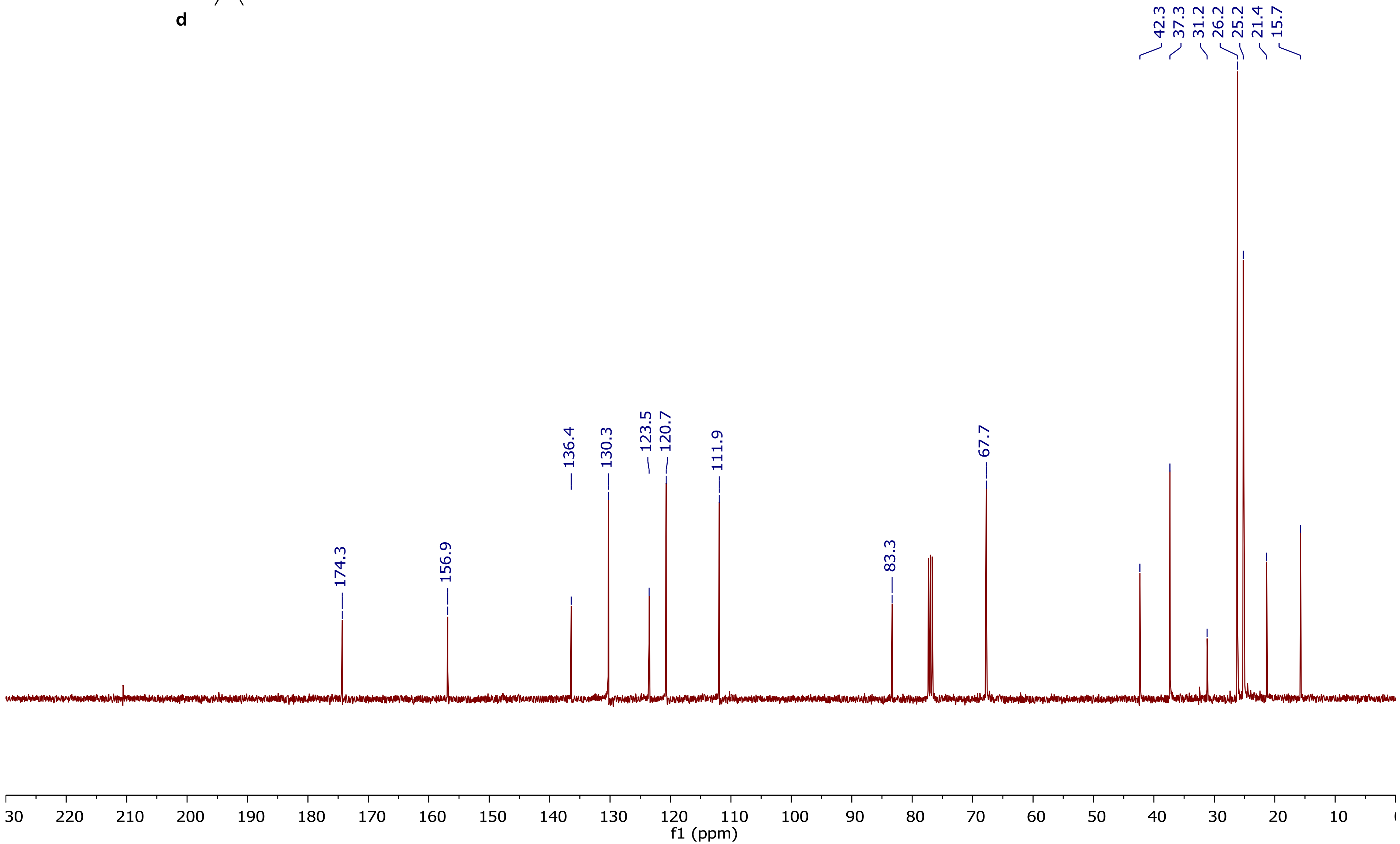

^{1}H NMR (400 MHz, $CDCl_3$)

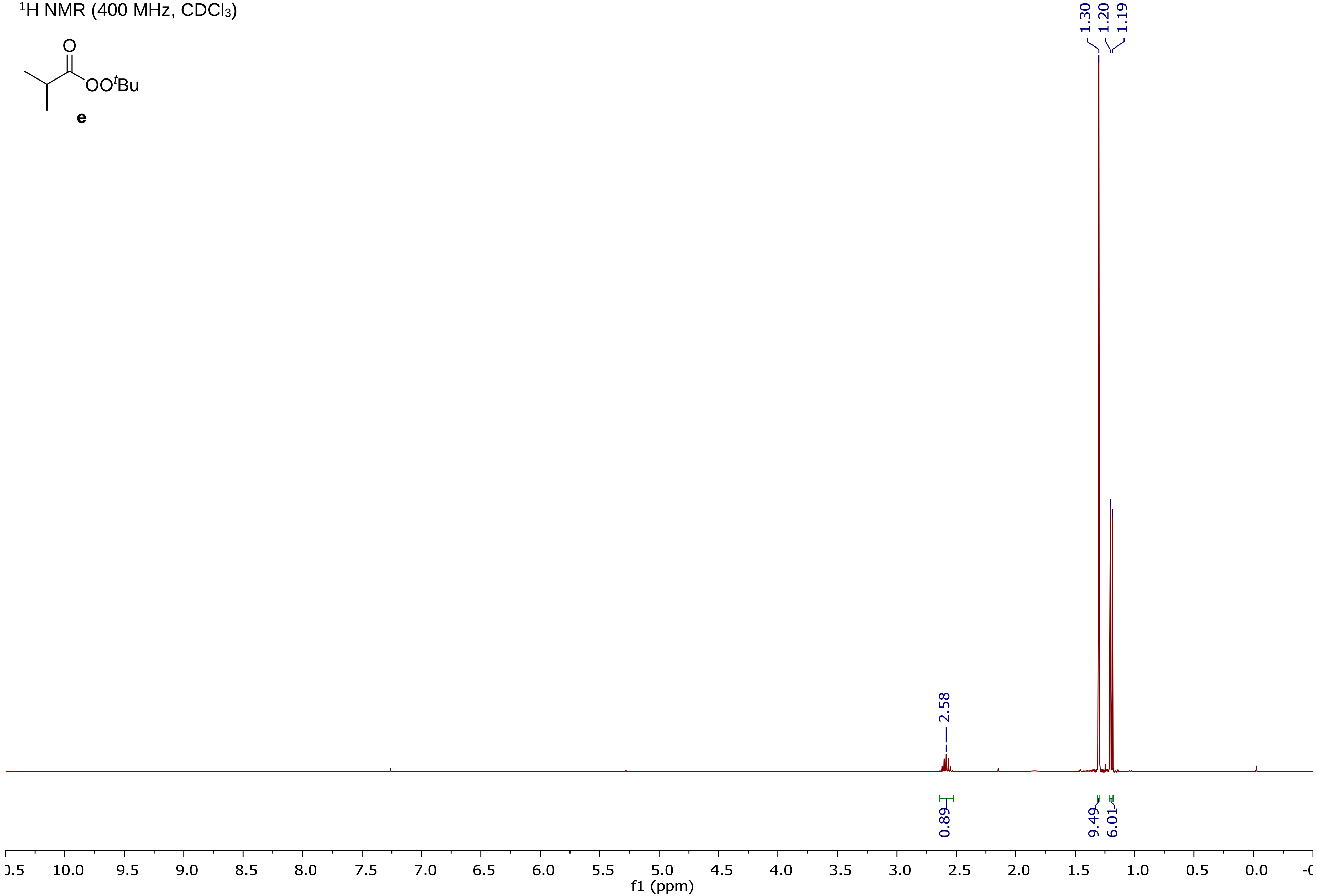

$^{13}C\{^{1}H\}$ NMR (100.6 MHz, $CDCl_3$)

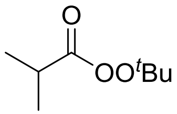

e

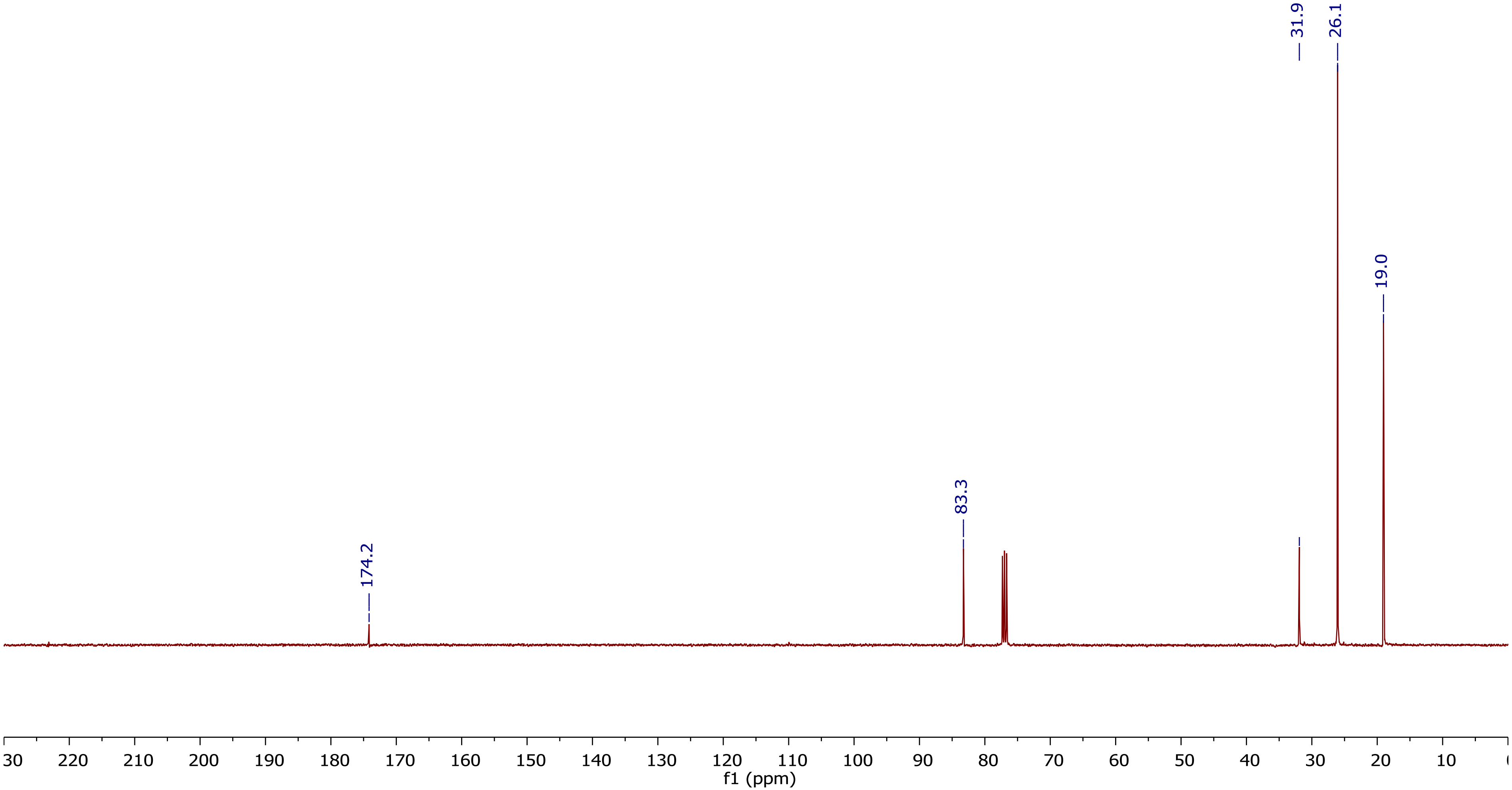

^{1}H NMR (400 MHz, $CDCl_3$)

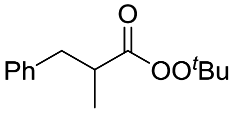

f

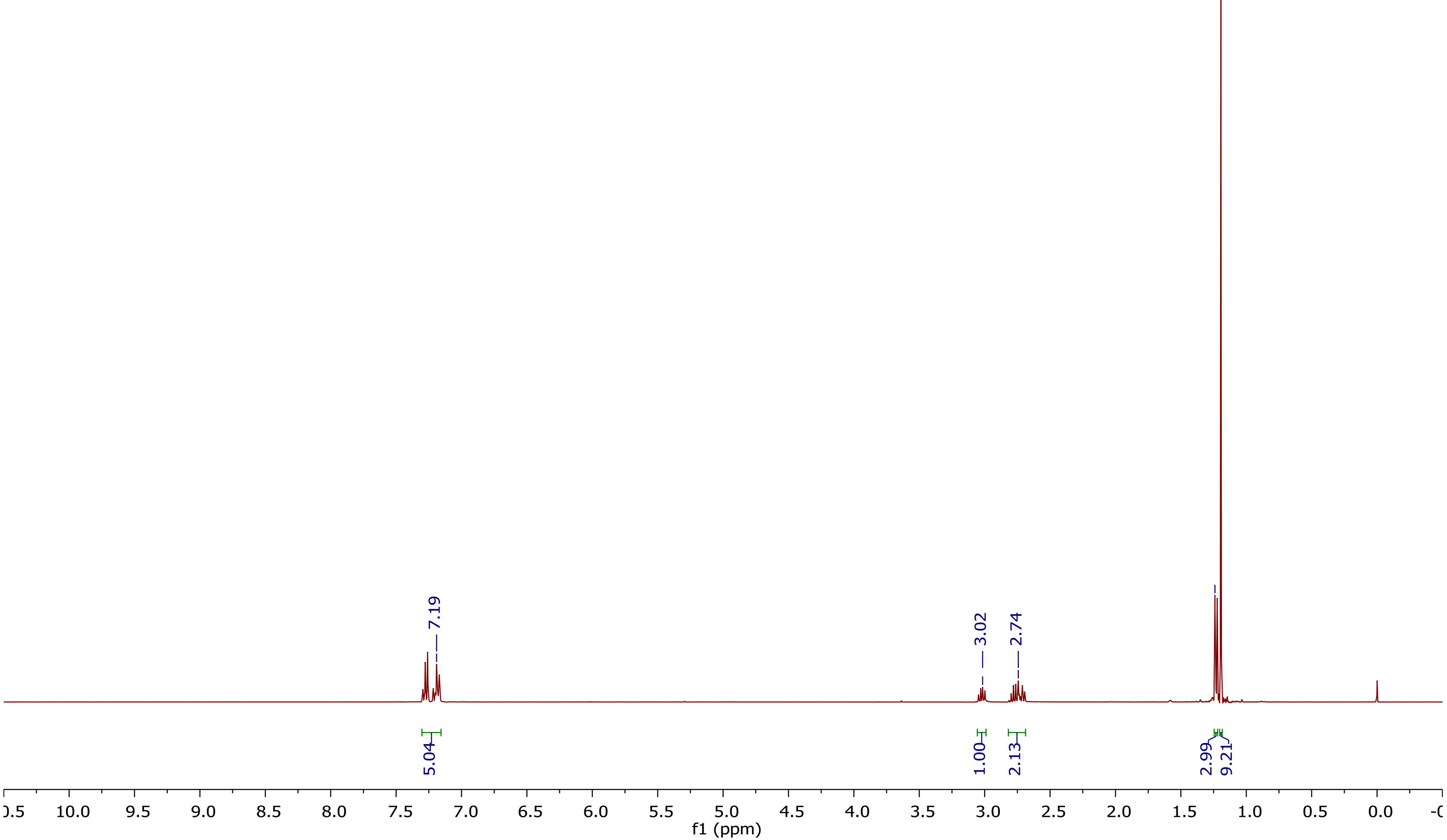

$^{13}C\{^1H\}$ NMR (100.6 MHz, $CDCl_3$)

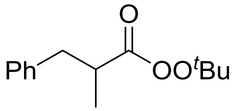

f

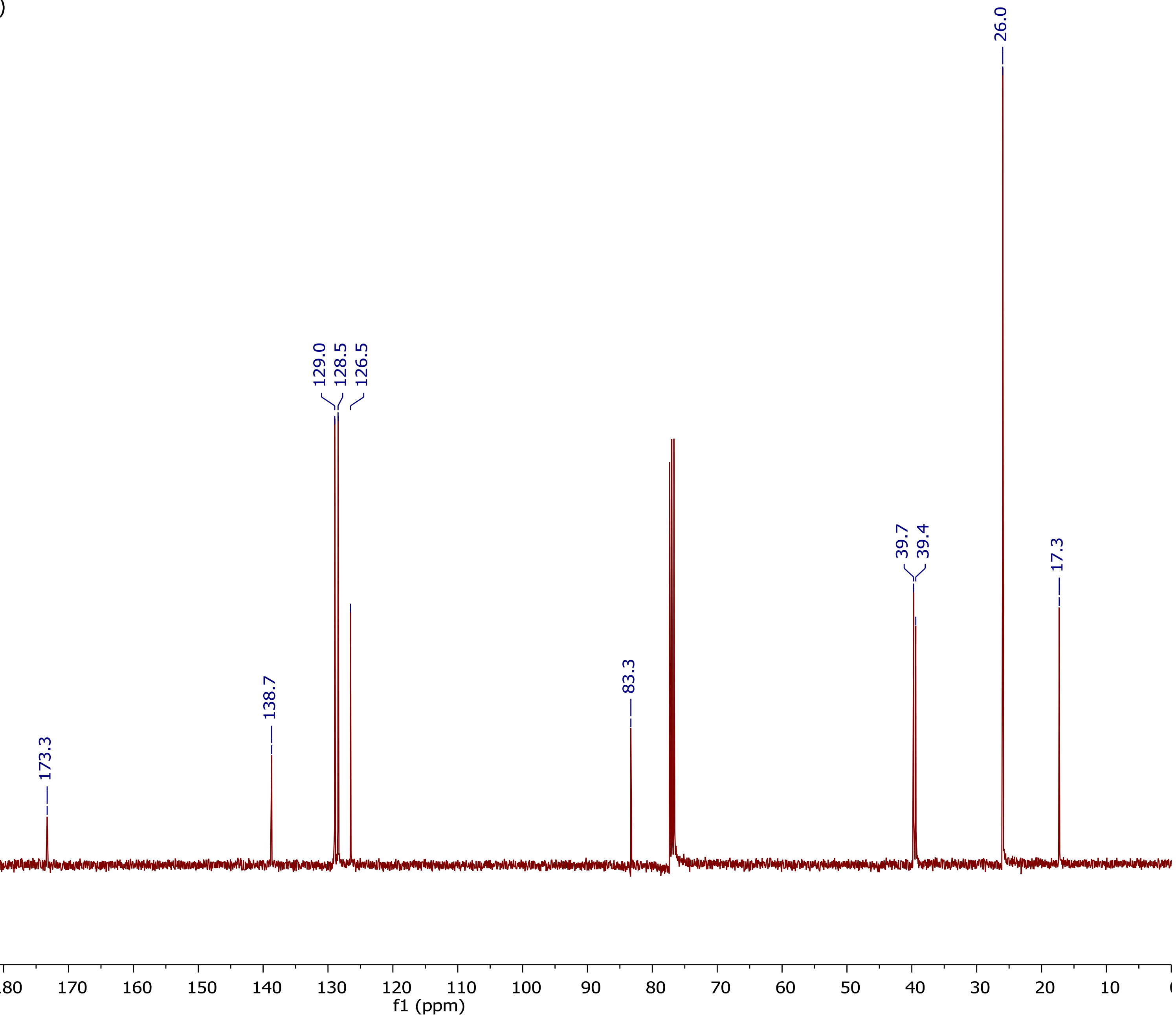

^{1}H NMR (400 MHz, $CDCl_3$)

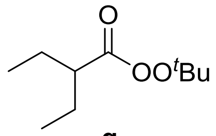

g

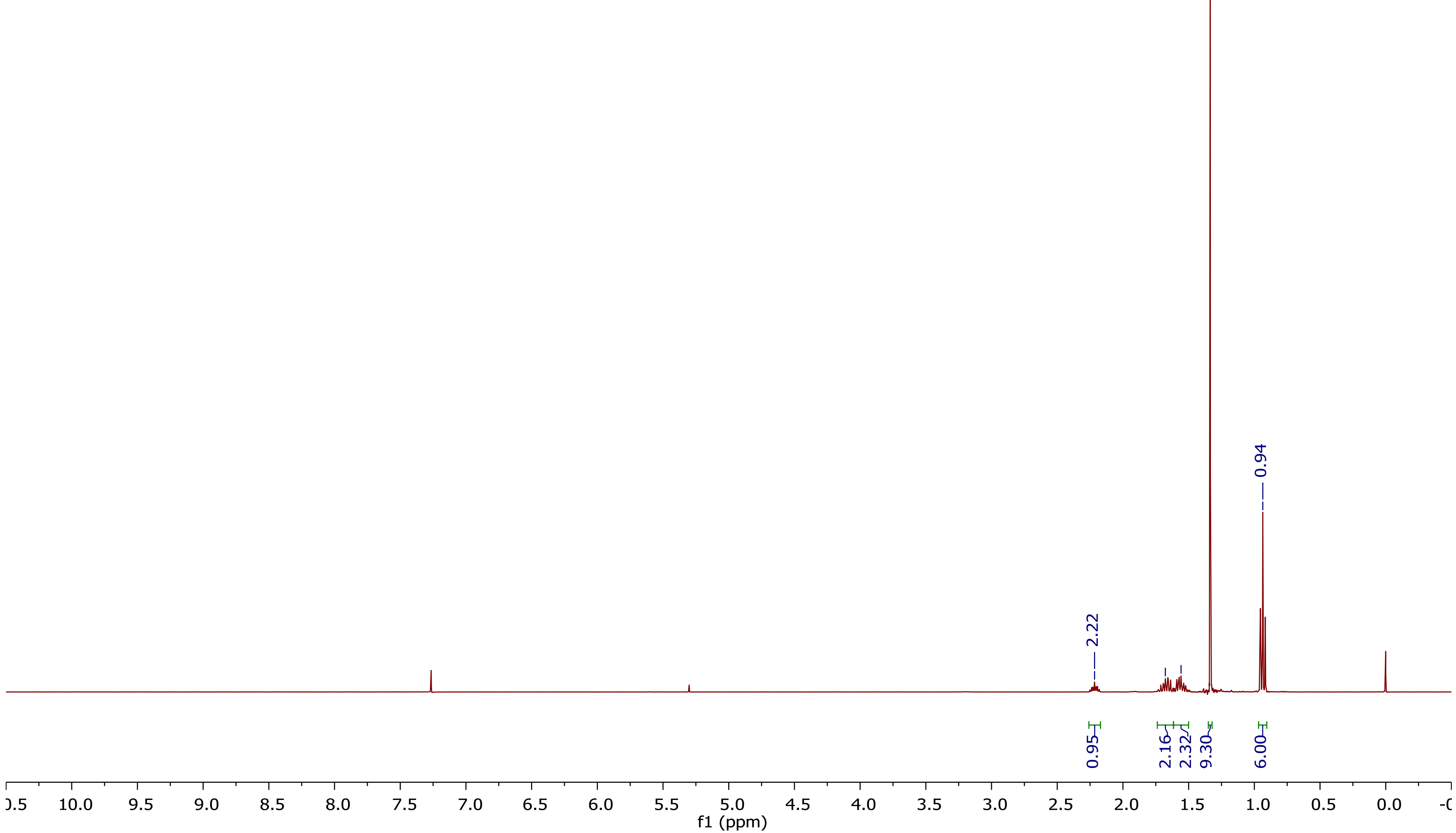

$^{13}C\{^{1}H\}$ NMR (100.6 MHz, $CDCl_3$)

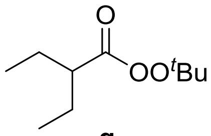

g

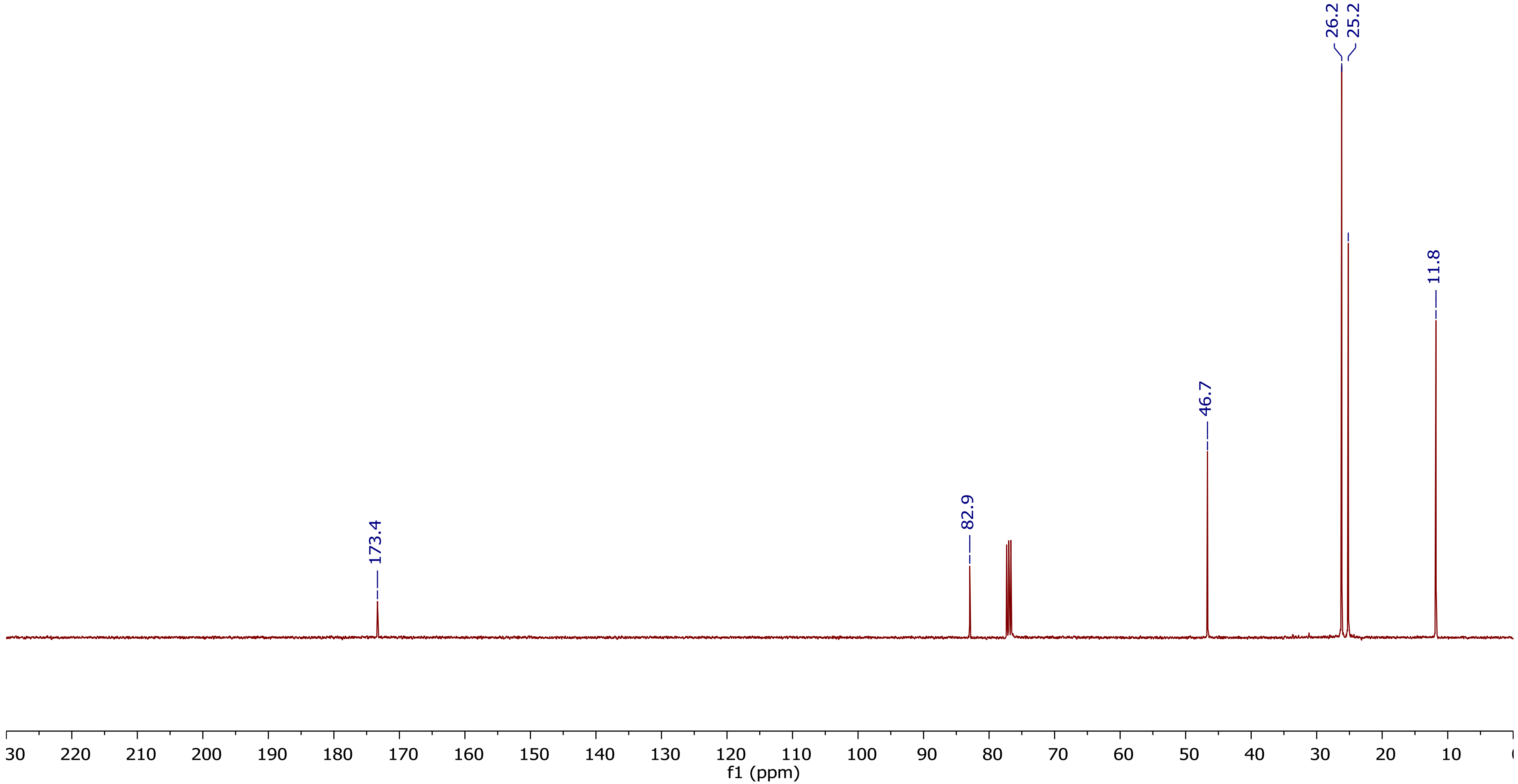

^{1}H NMR (400 MHz, $CDCl_3$)

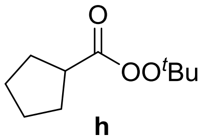

h

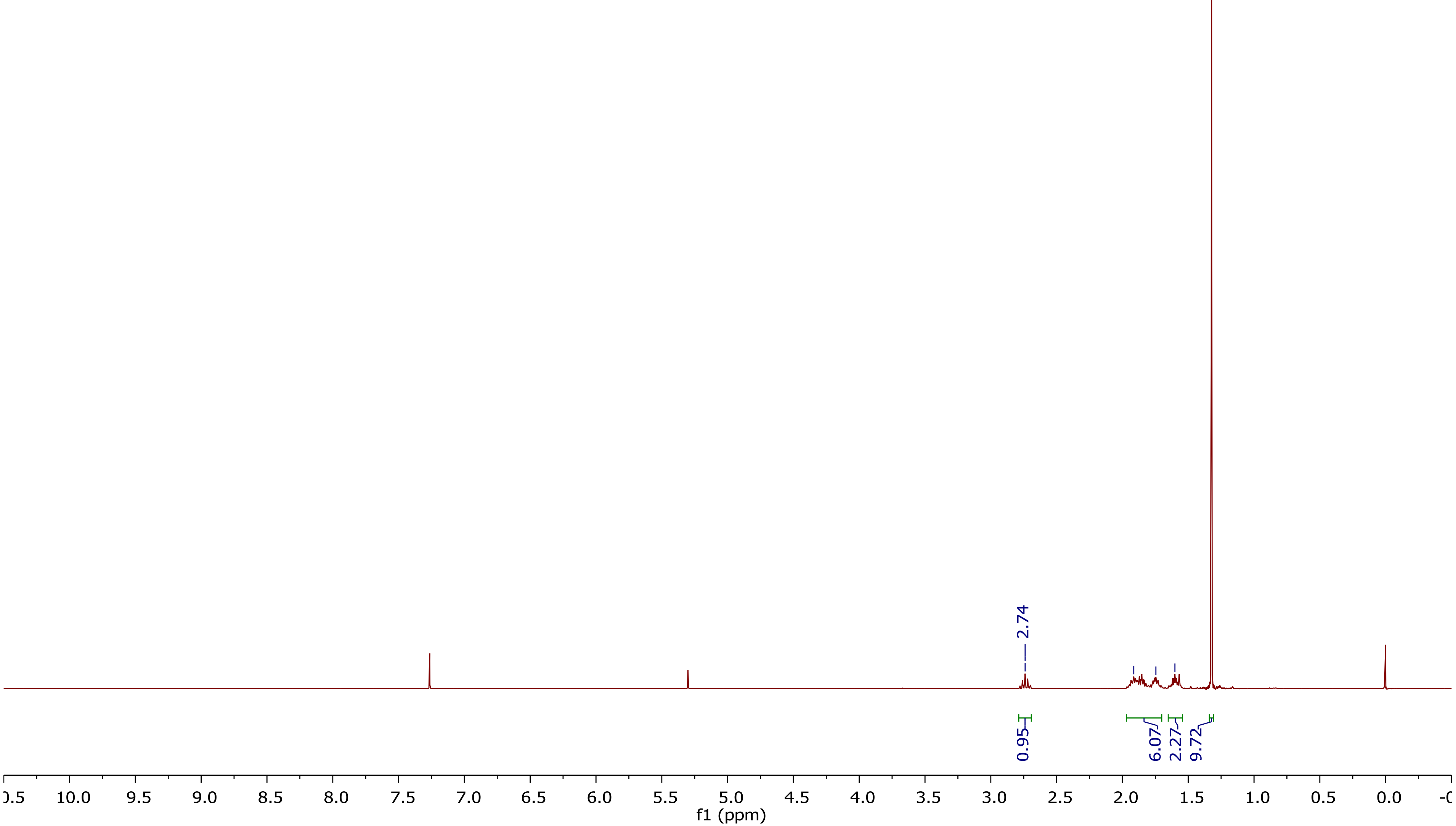

$^{13}C\{^1H\}$ NMR (100.6 MHz, $CDCl_3$)

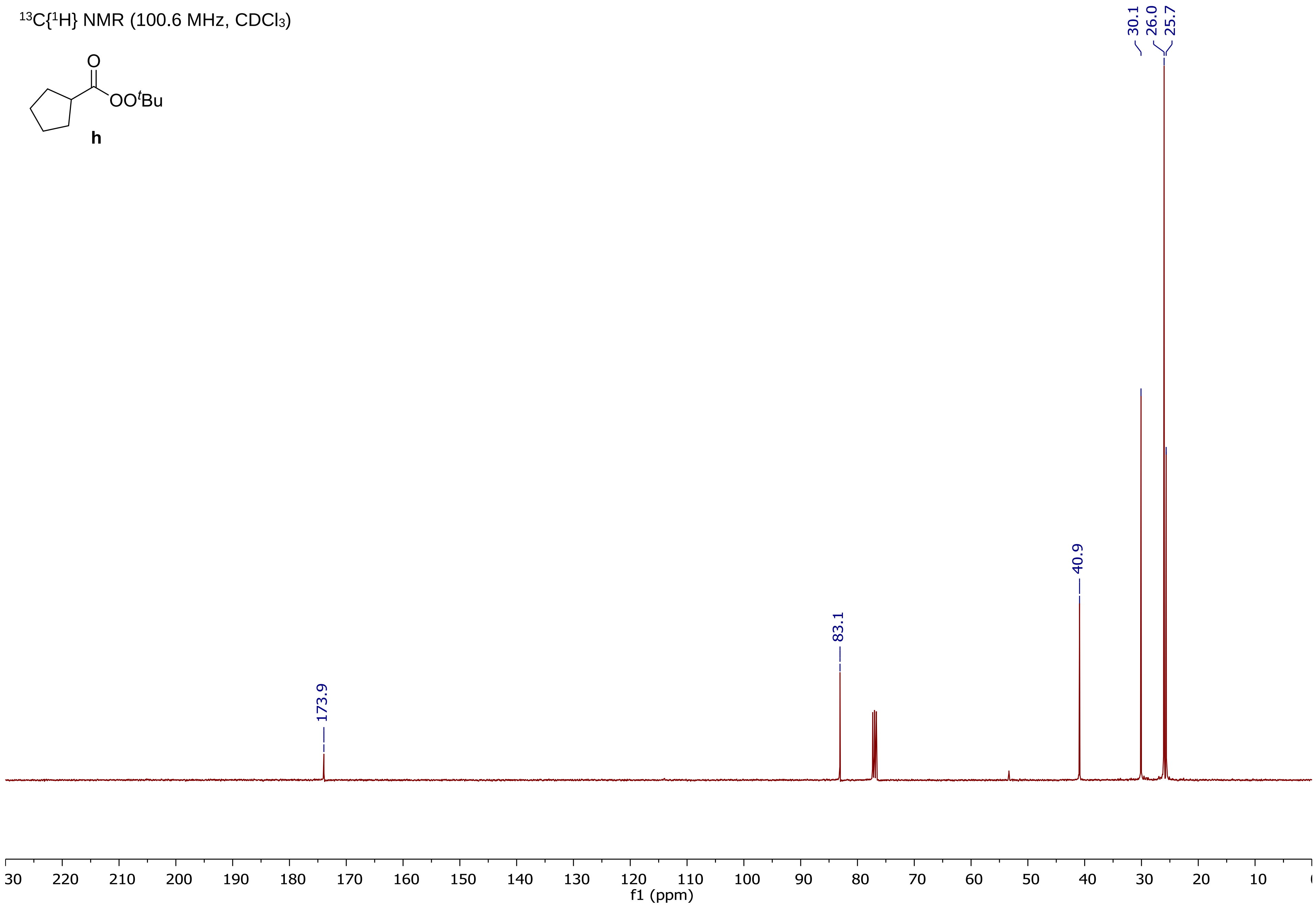

^{1}H NMR (400 MHz, $CDCl_3$)

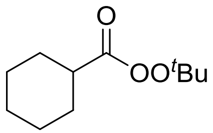

i

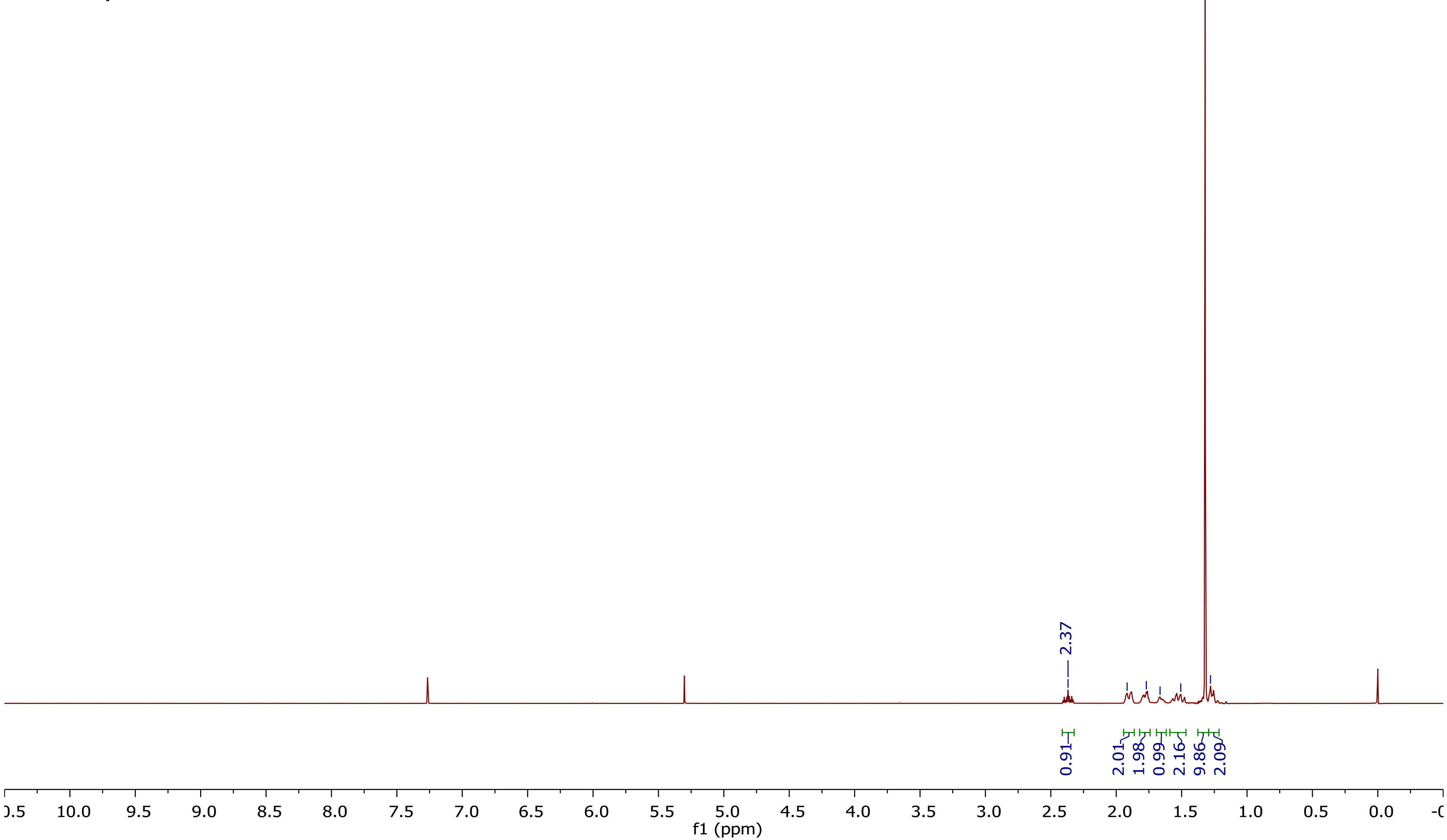

$^{13}C\{^{1}H\}$ NMR (100.6 MHz, $CDCl_3$)

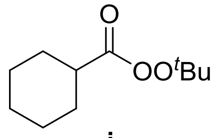

i

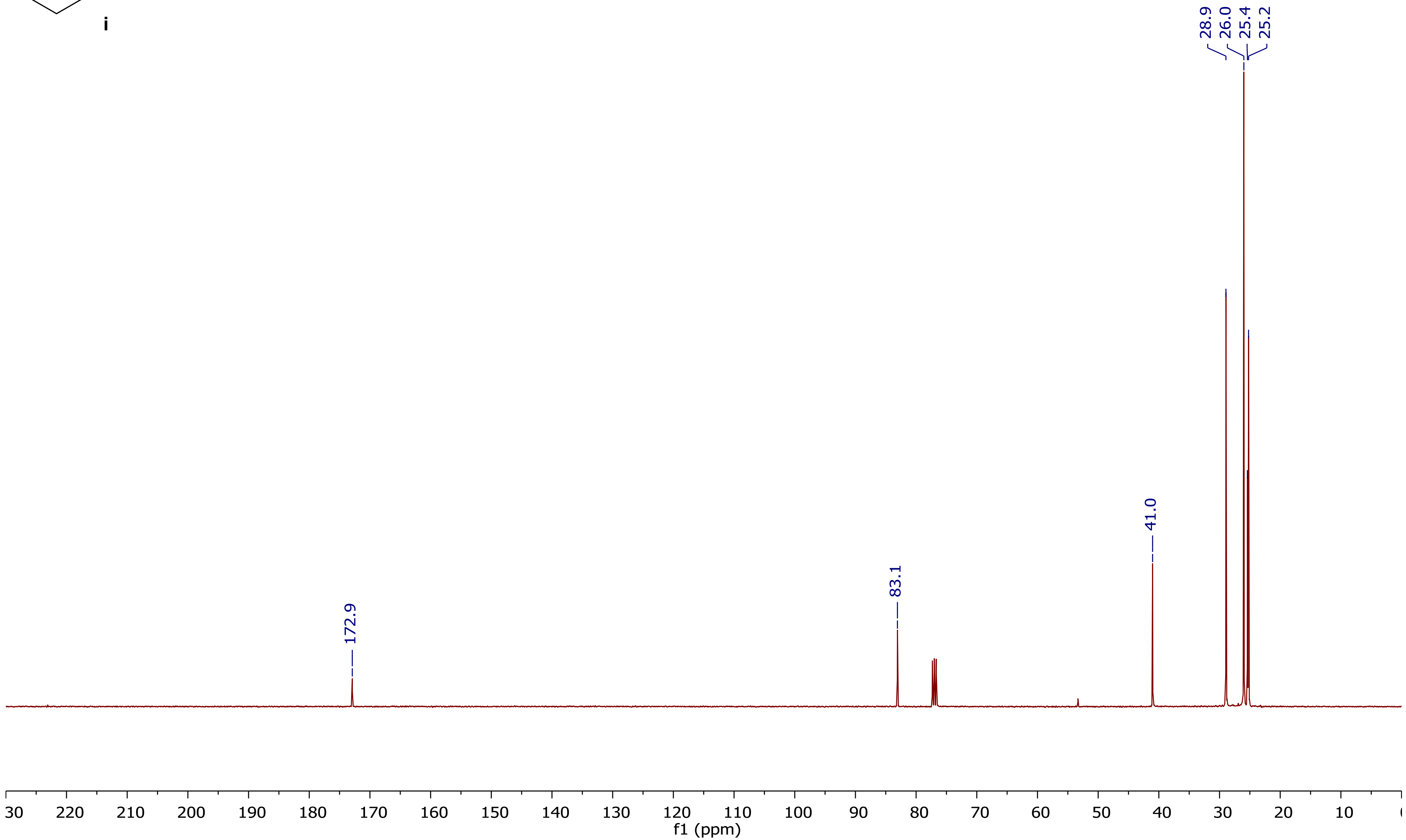

^{1}H NMR (400 MHz, $CDCl_3$)

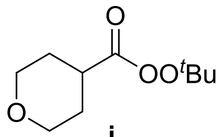

j

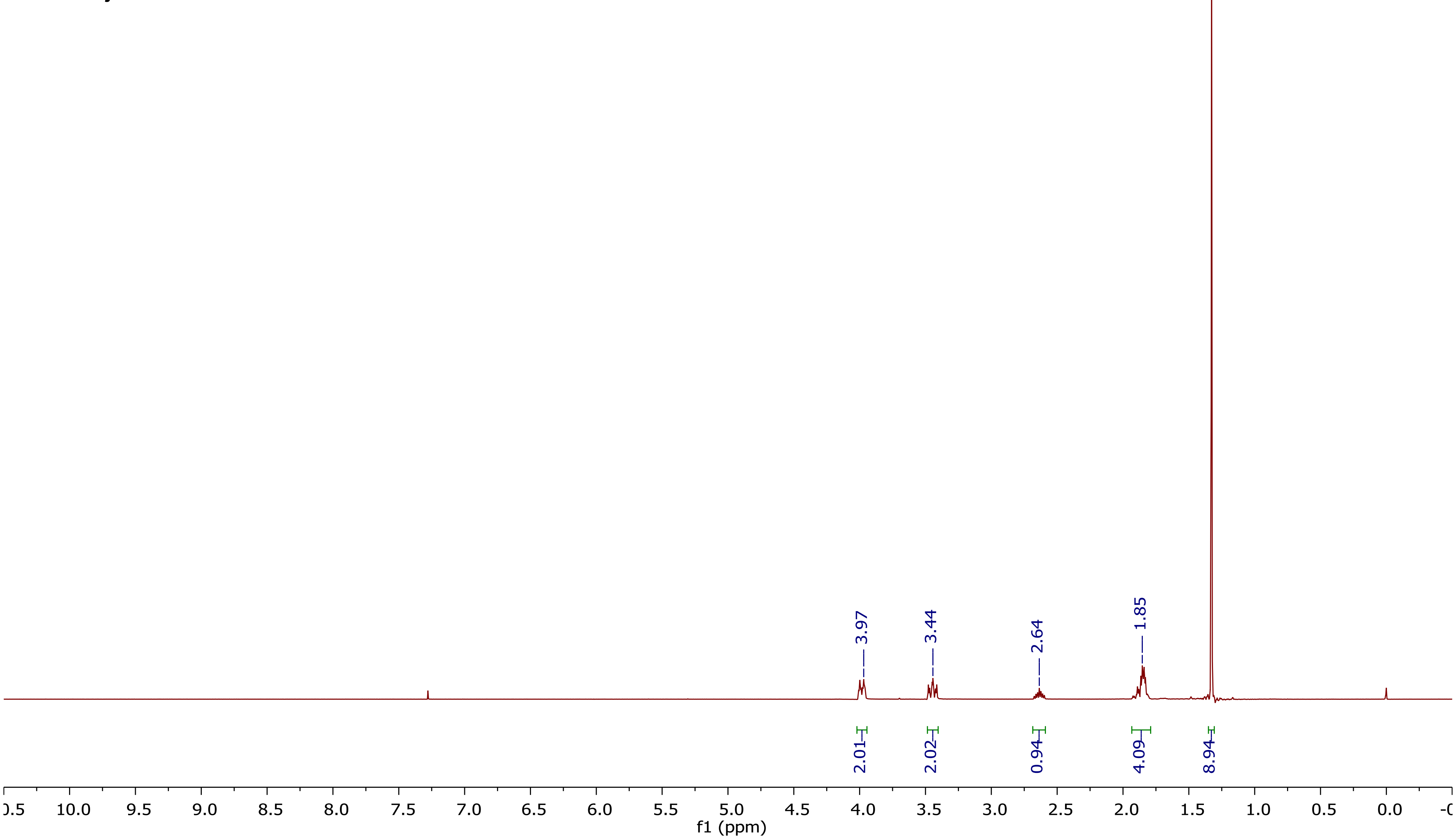

$^{13}C\{^{1}H\}$ NMR (100.6 MHz, $CDCl_3$)

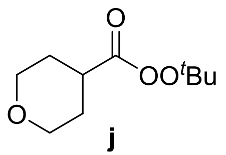

j

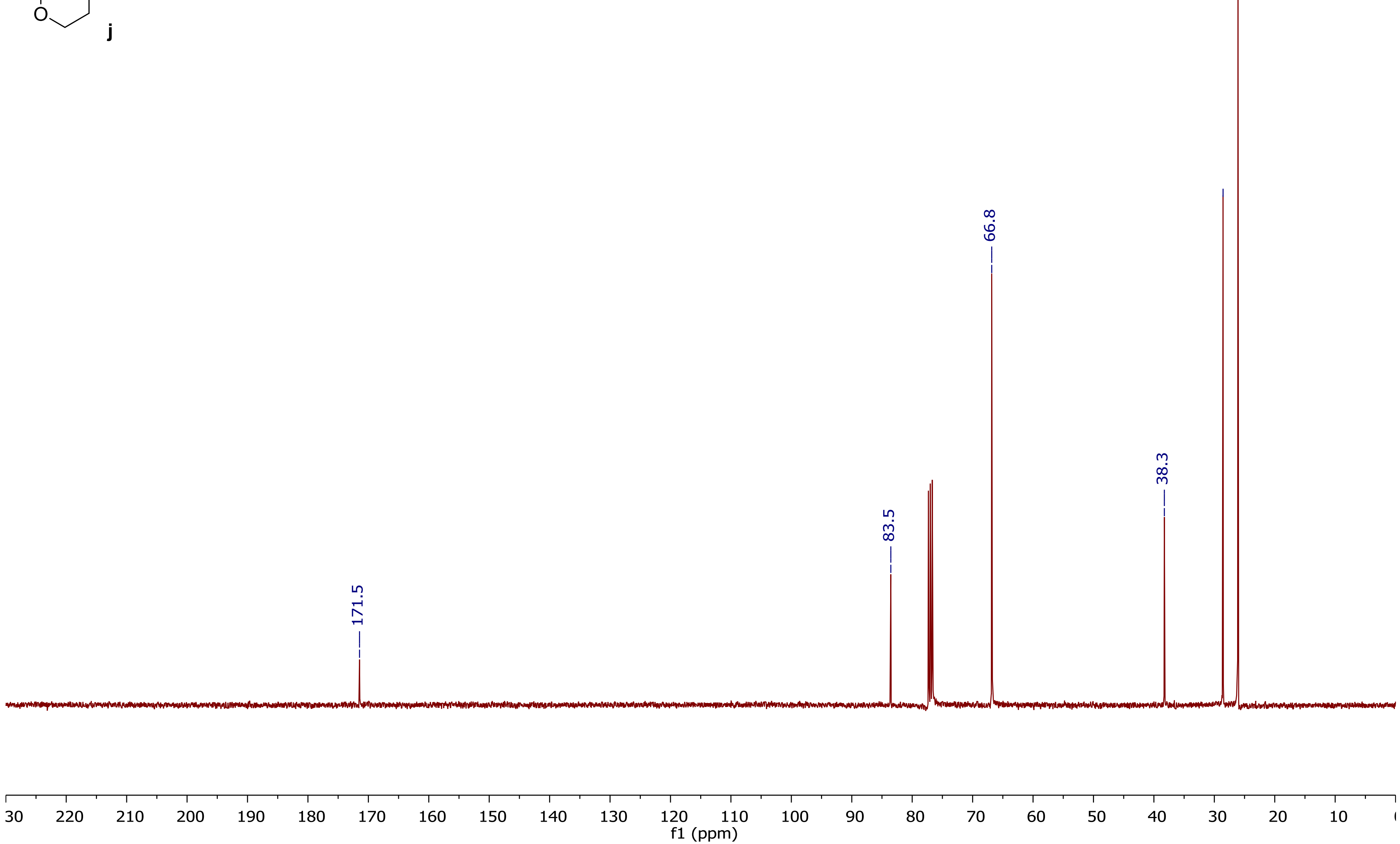

^{1}H NMR (400 MHz, $CDCl_3$)

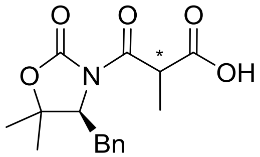

SI2

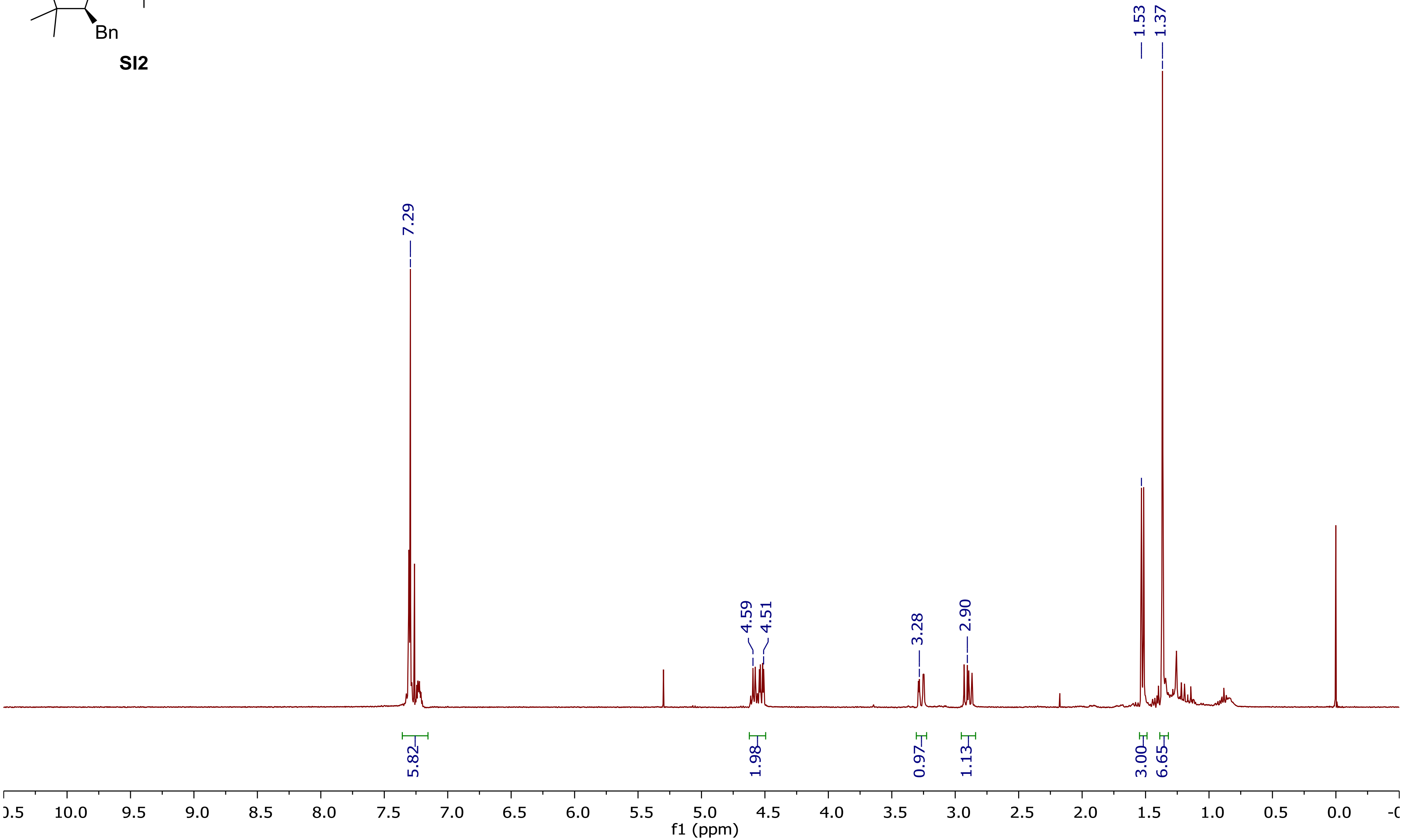

$^{13}C\{^{1}H\}$ NMR (100.6 MHz, $CDCl_3$)

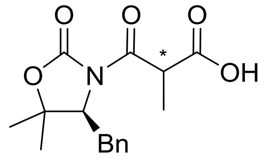

SI2

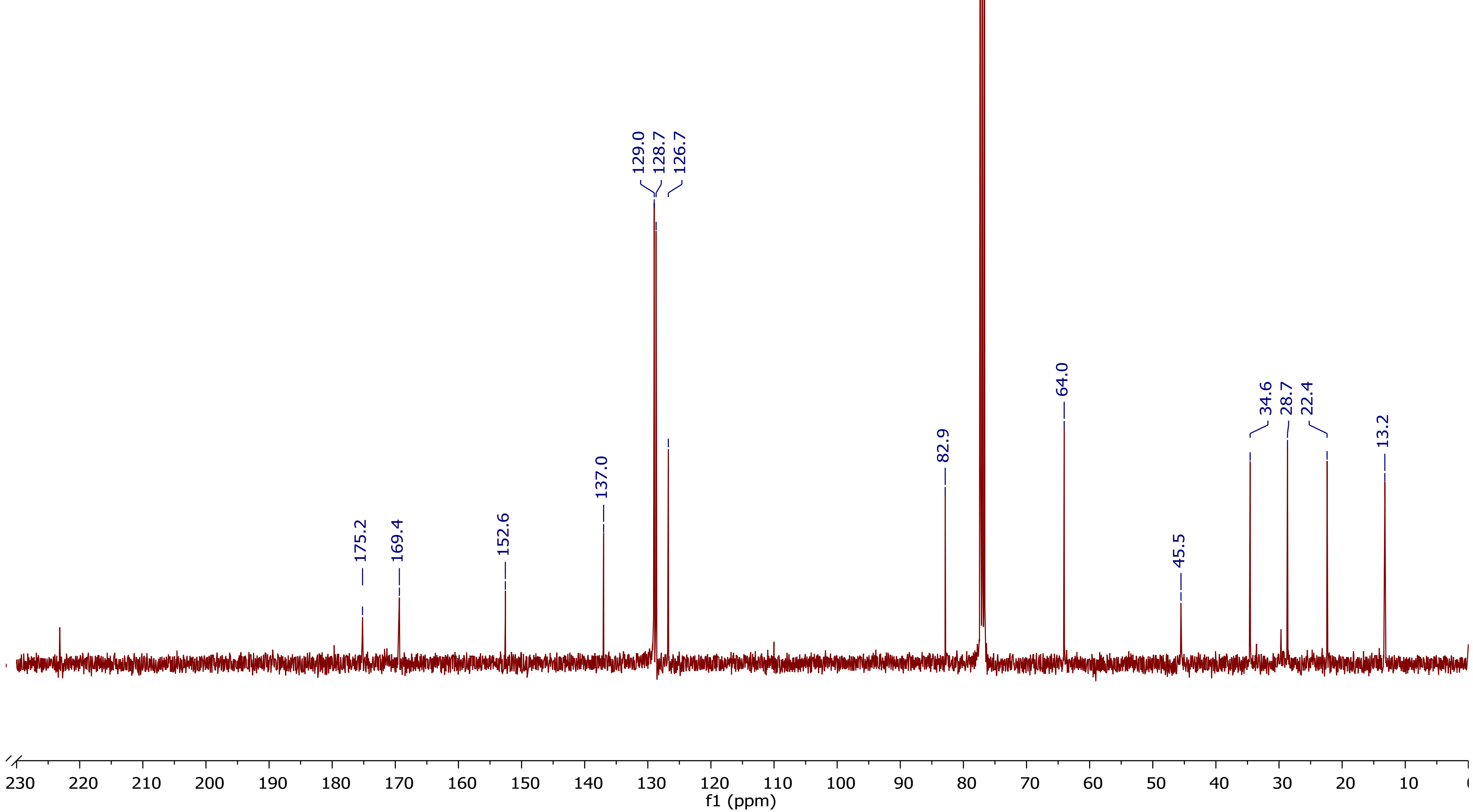

^{1}H NMR (400 MHz, $CDCl_3$)

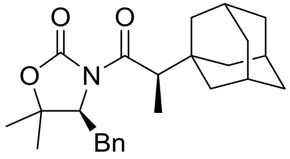

1a

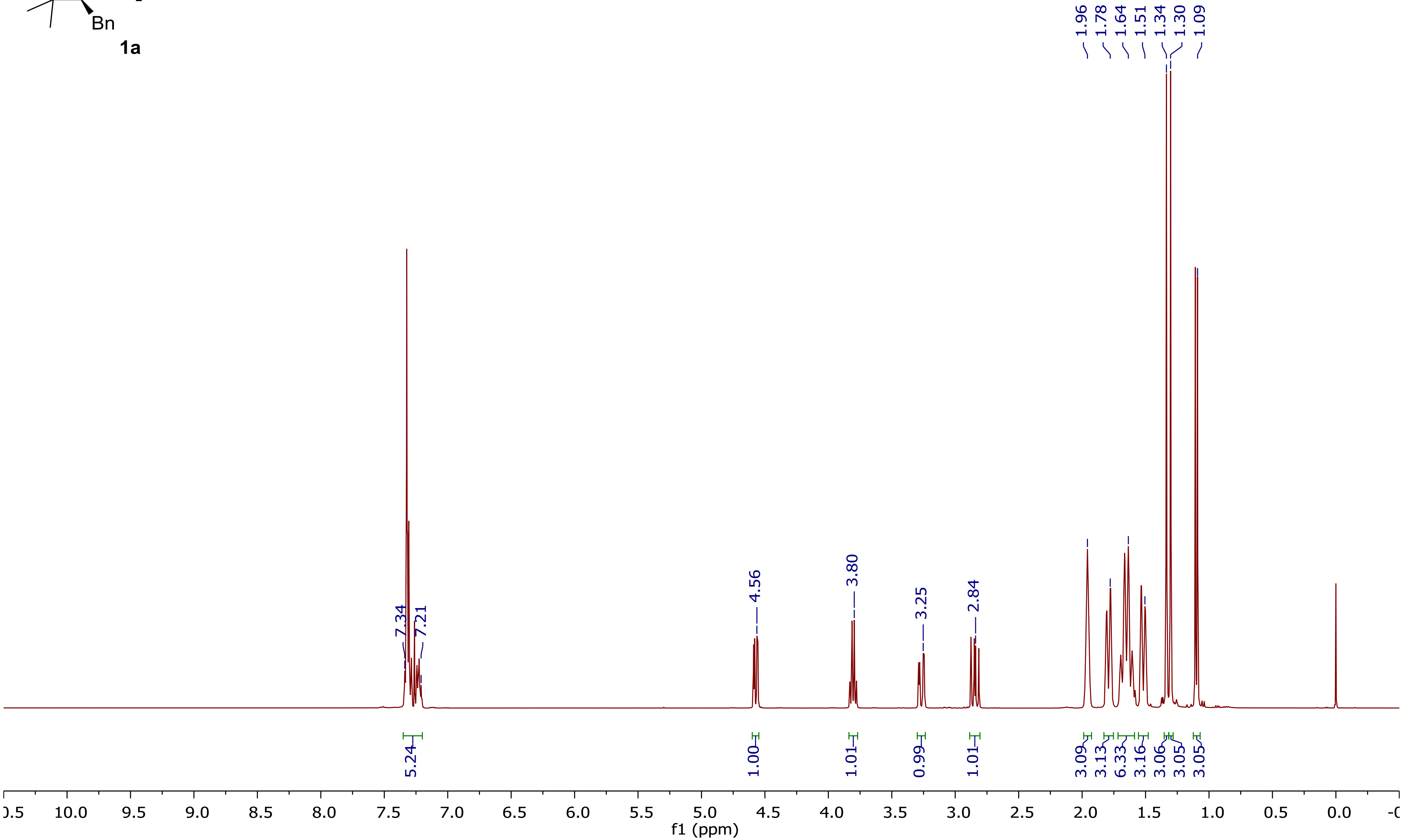

$^{13}C\{^{1}H\}$ NMR (100.6 MHz, $CDCl_3$)

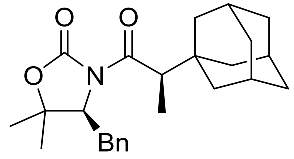

1a

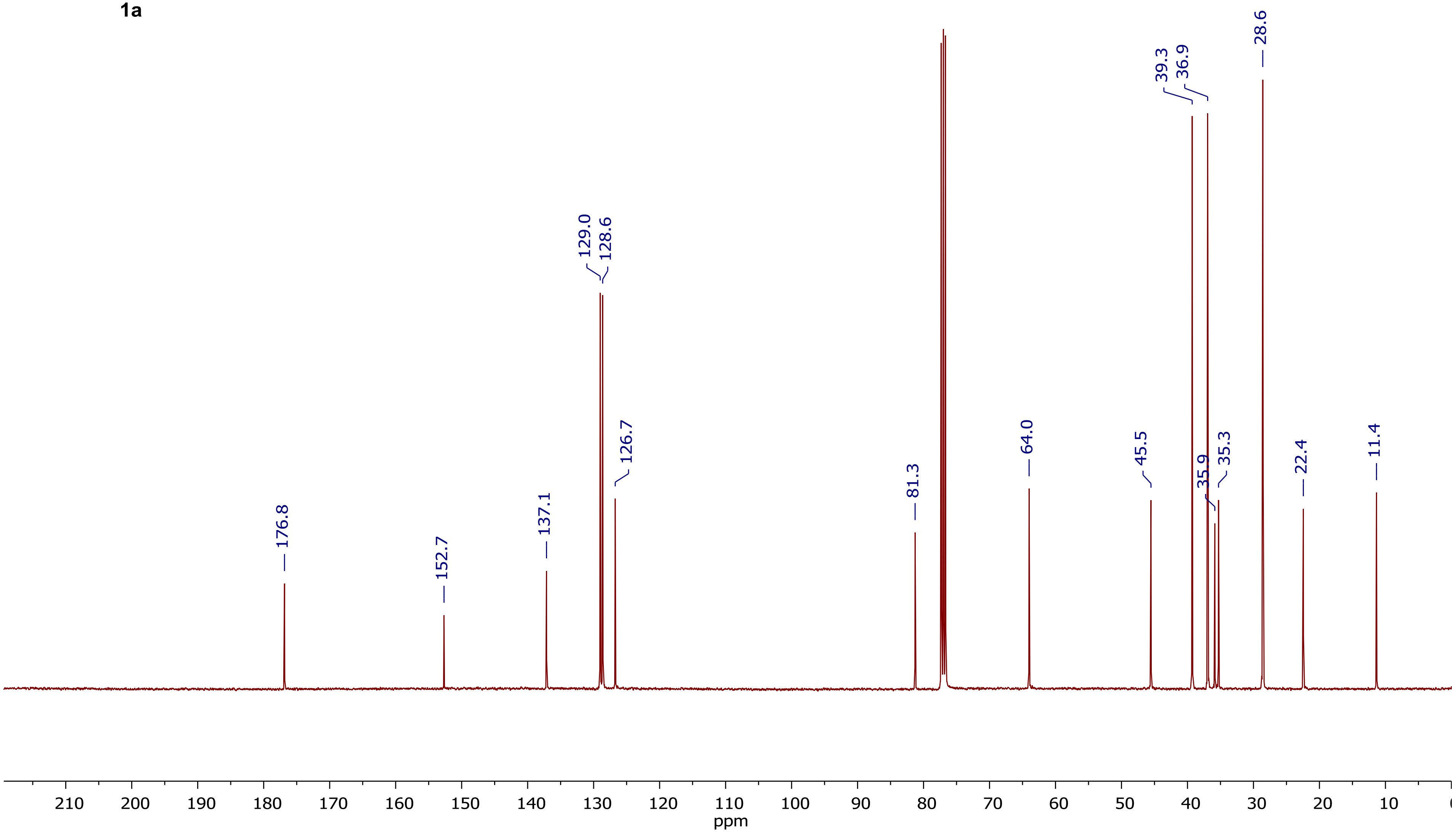

^1H NMR (400 MHz, $CDCl_3$)

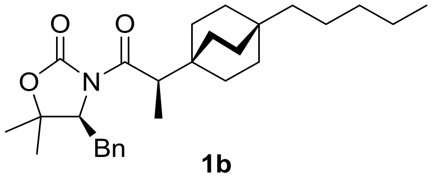

1b

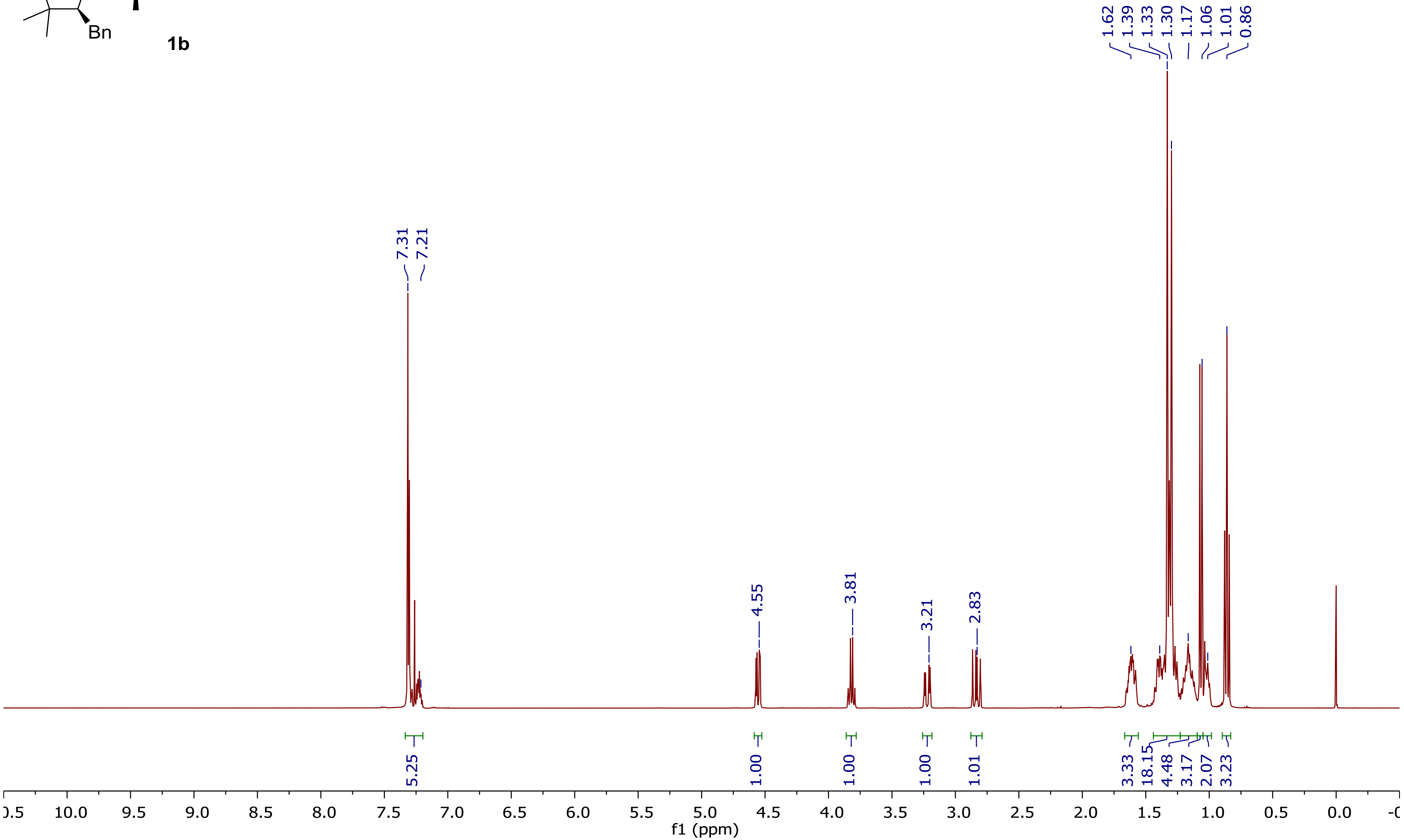

$^{13}C\{^{1}H\}$ NMR (100.6 MHz, $CDCl_3$)

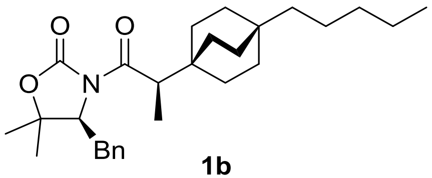

1b

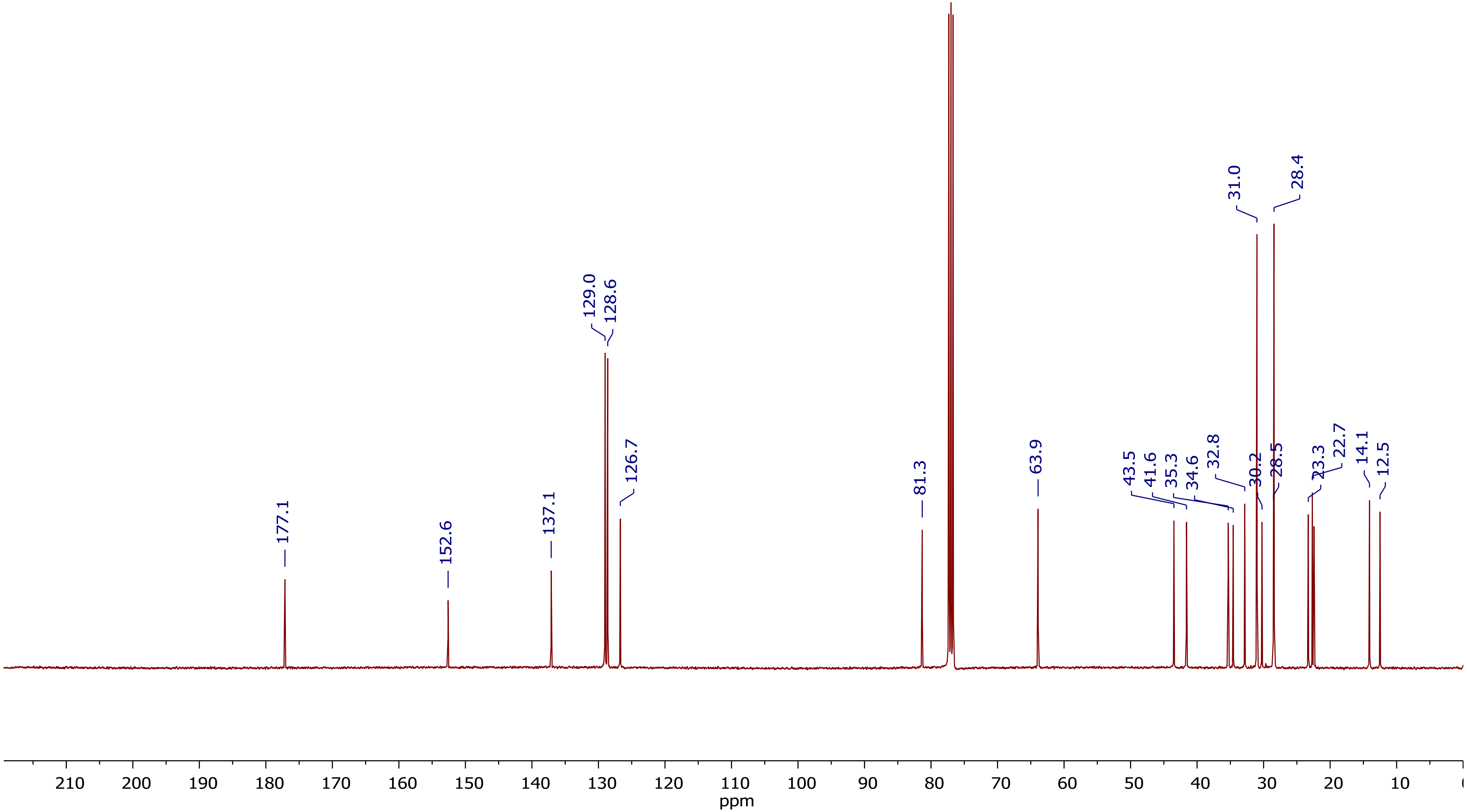

^{1}H NMR (400 MHz, $CDCl_3$)

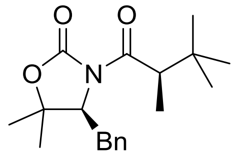

1c

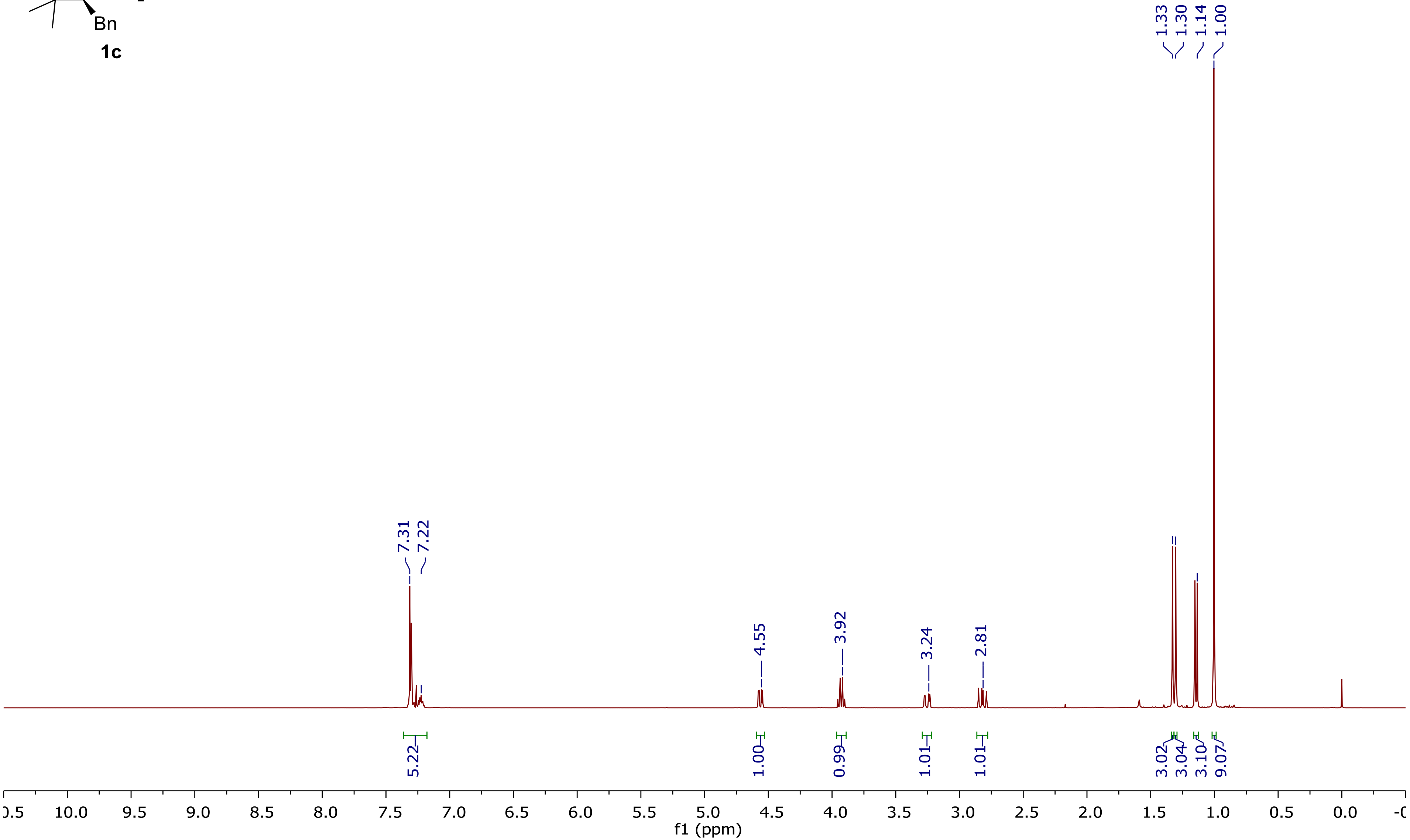

$^{13}C\{^1H\}$ NMR (100.6 MHz, $CDCl_3$)

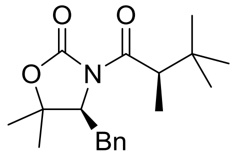

1c

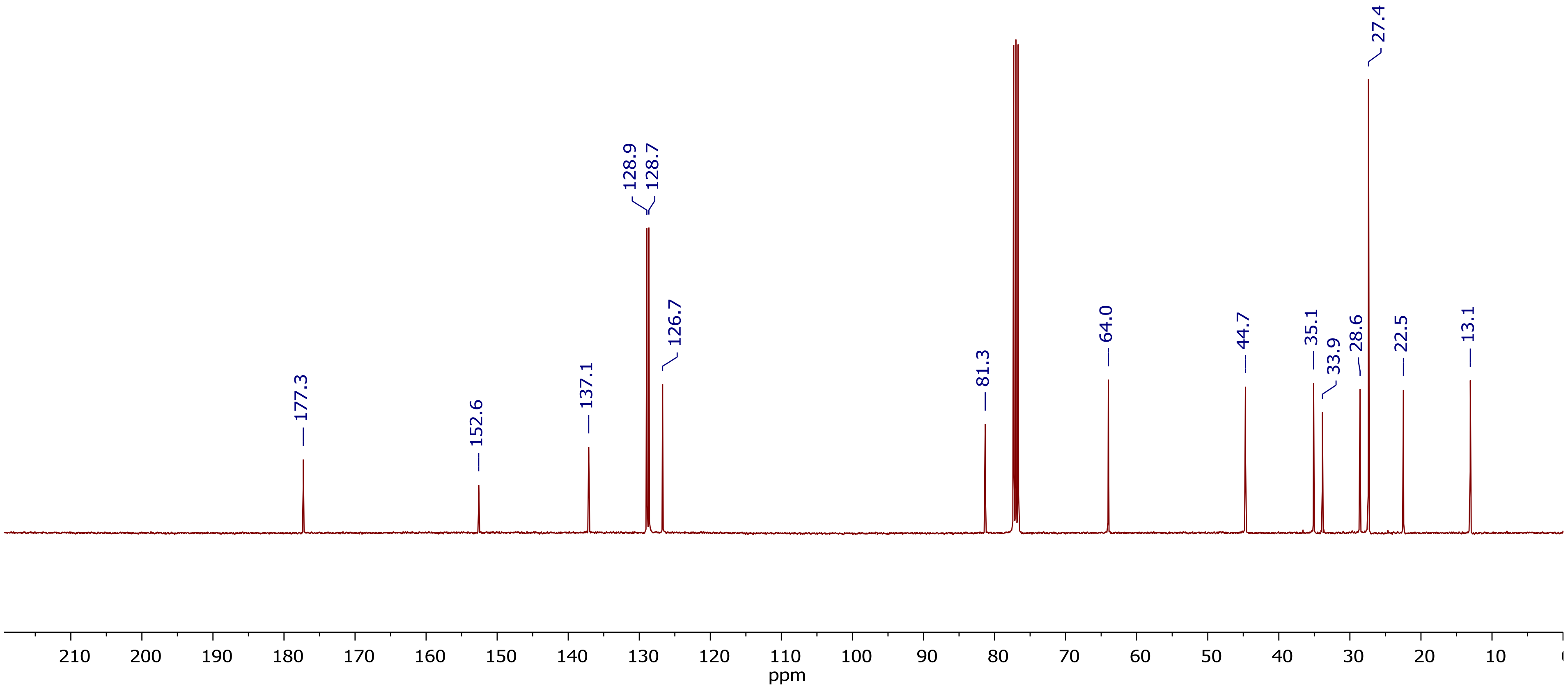

^1H NMR (400 MHz, $CDCl_3$)

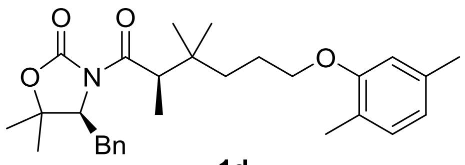

1d

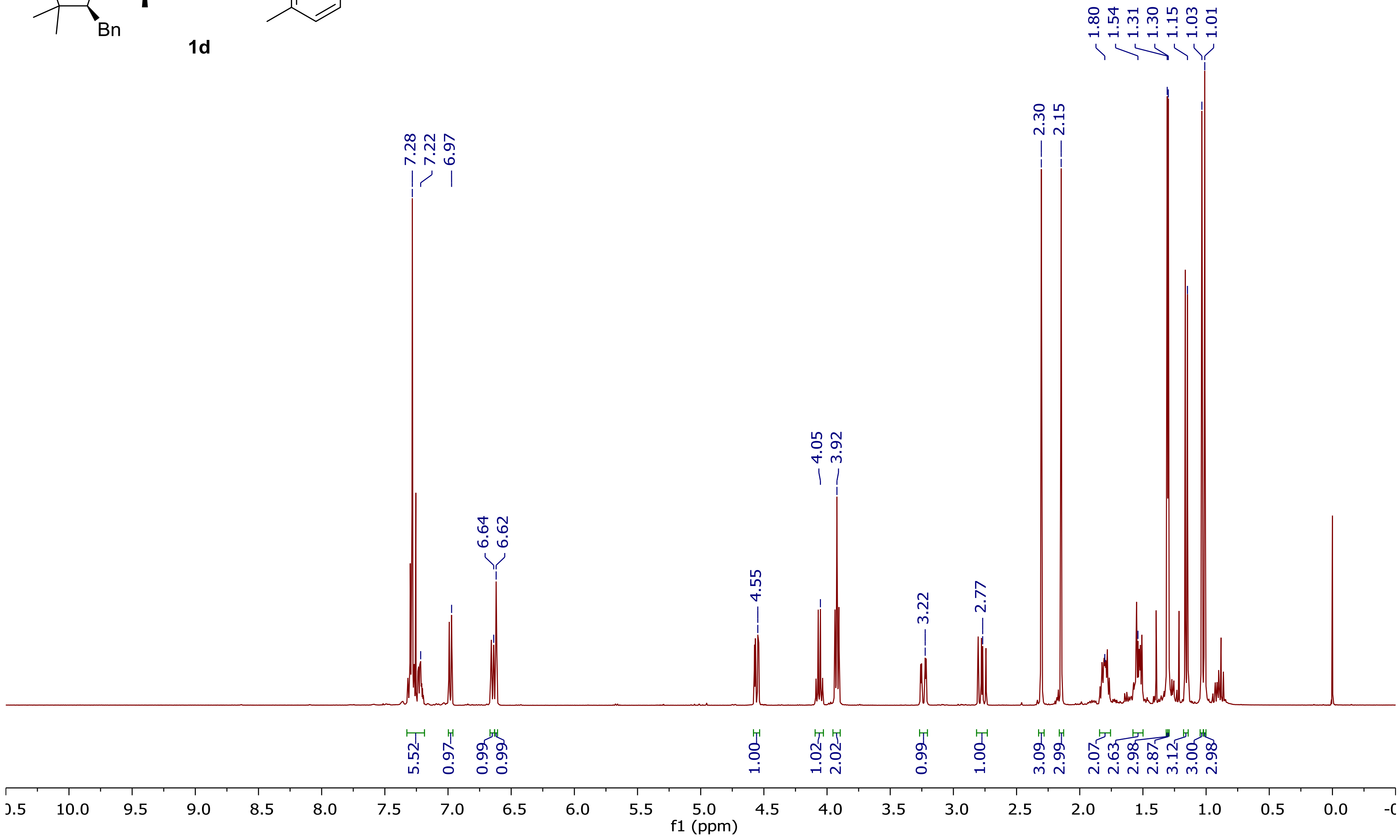

$^{13}C\{^{1}H\}$ NMR (100.6 MHz, $CDCl_3$)

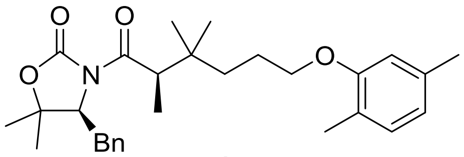

1d

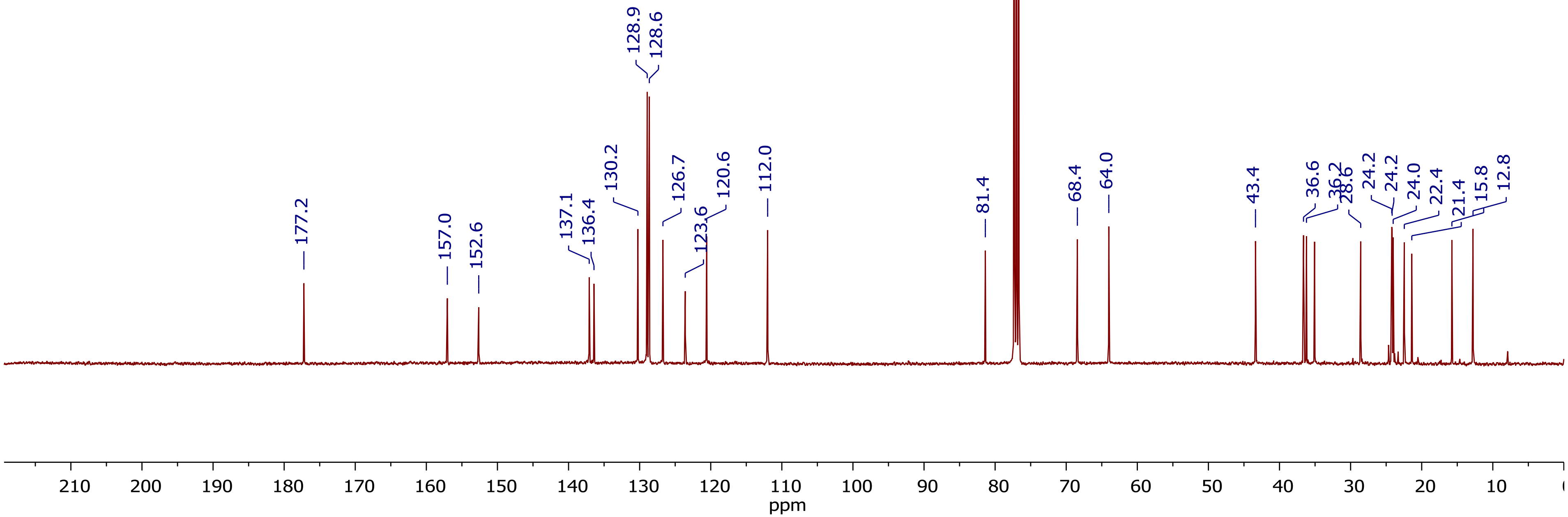

^{1}H NMR (400 MHz, $CDCl_3$)

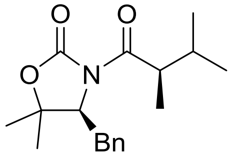

1e

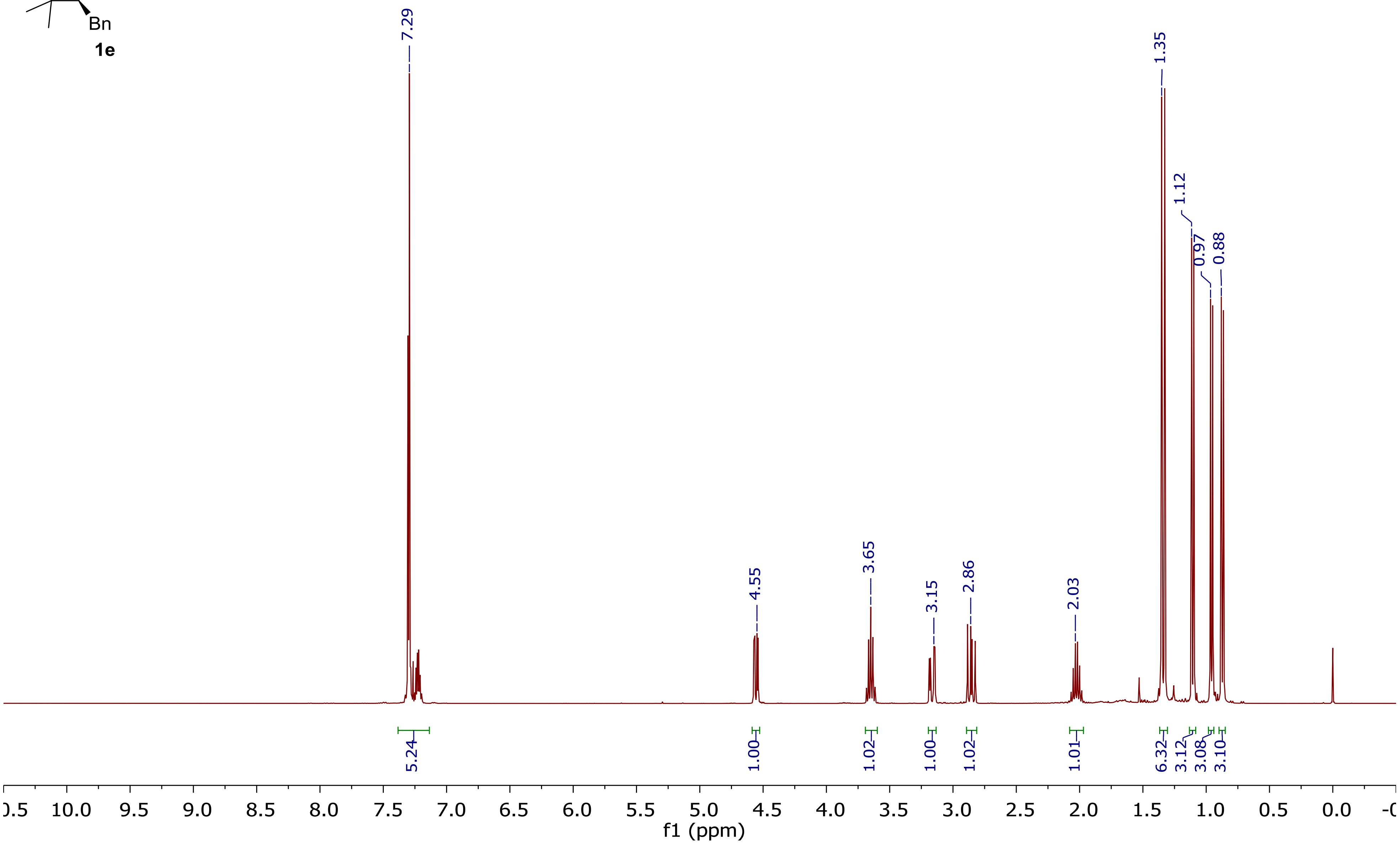

$^{13}C\{^{1}H\}$ NMR (100.6 MHz, $CDCl_3$)

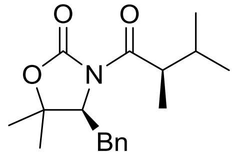

1e

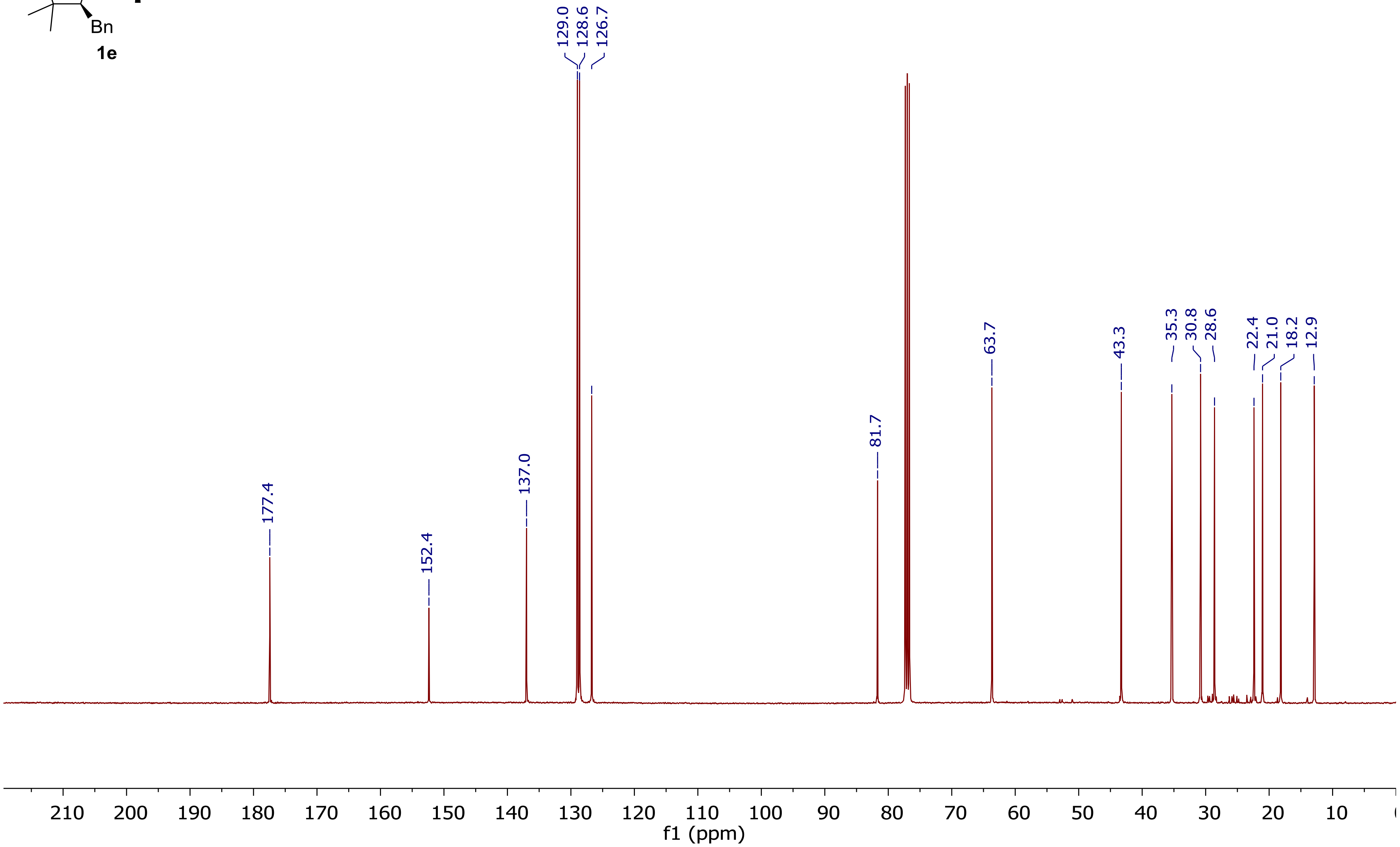

^{1}H NMR (400 MHz, $CDCl_3$)

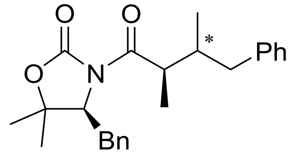

1f
(major)

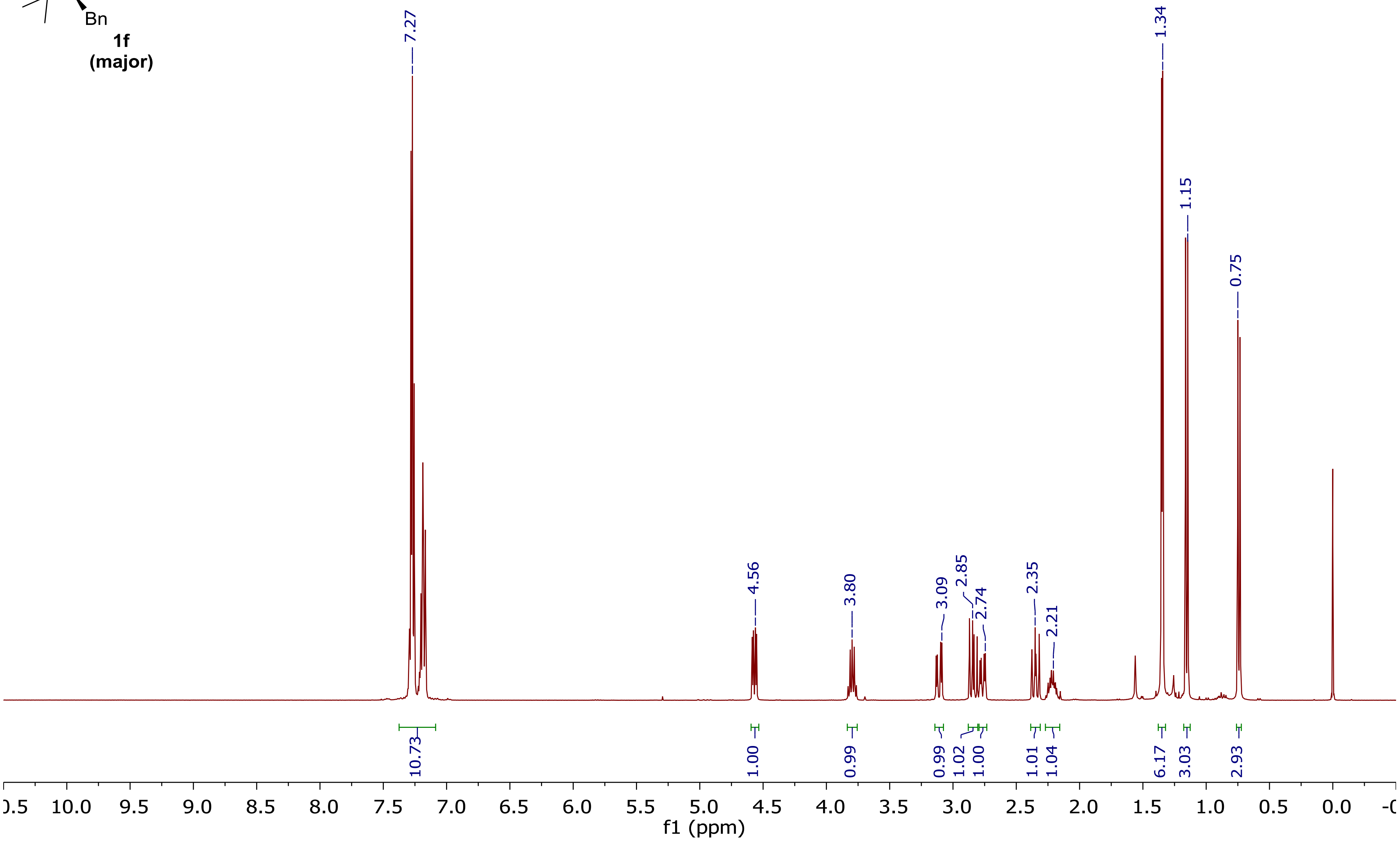

$^{13}C\{^{1}H\}$ NMR (100.6 MHz, $CDCl_3$)

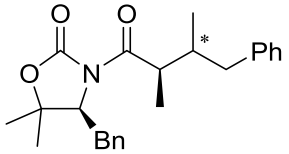

1f
(major)

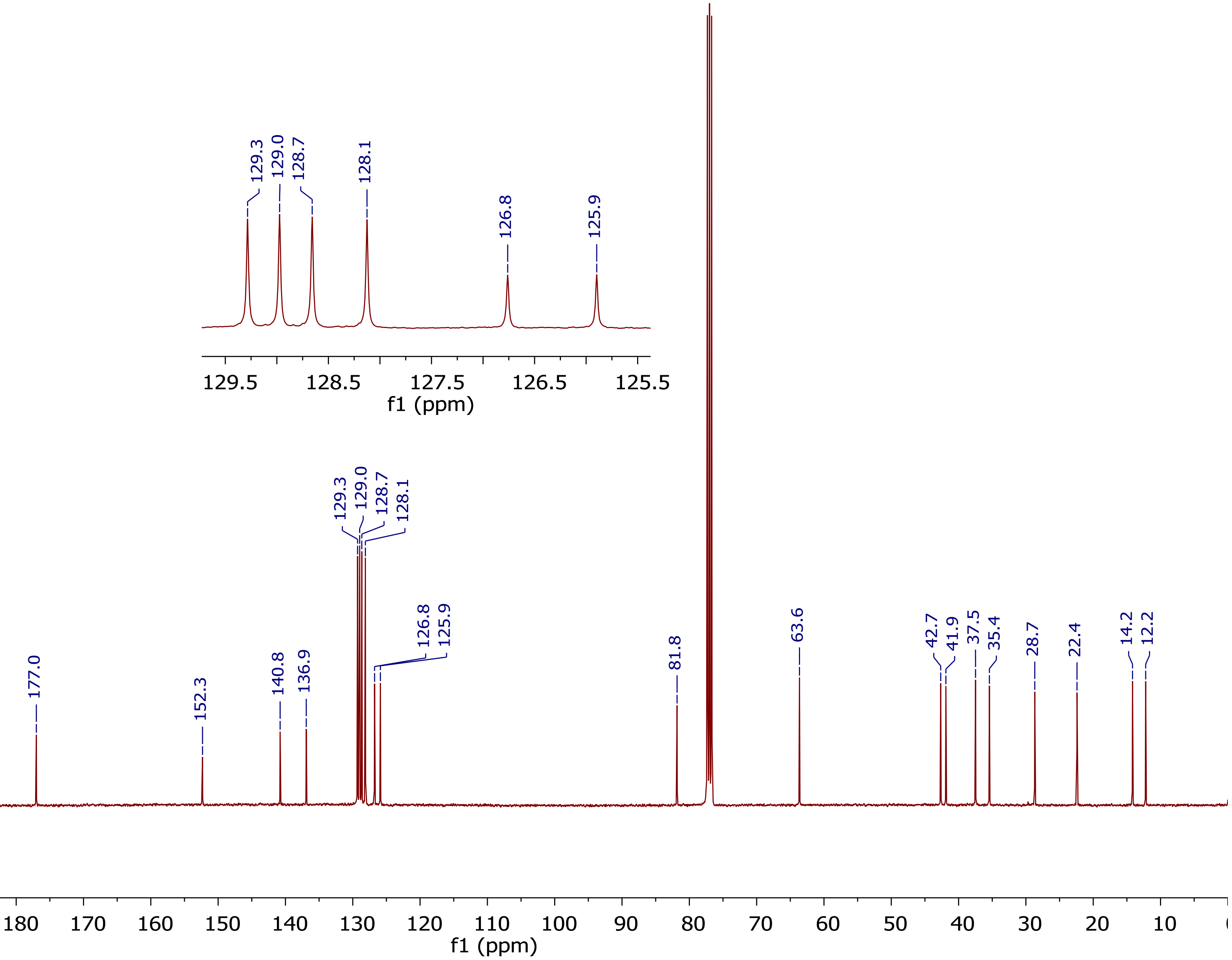

^{1}H NMR (400 MHz, CDCl$_3$)

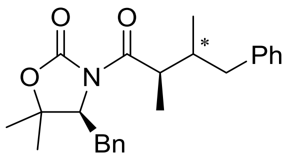

1f
(minor)

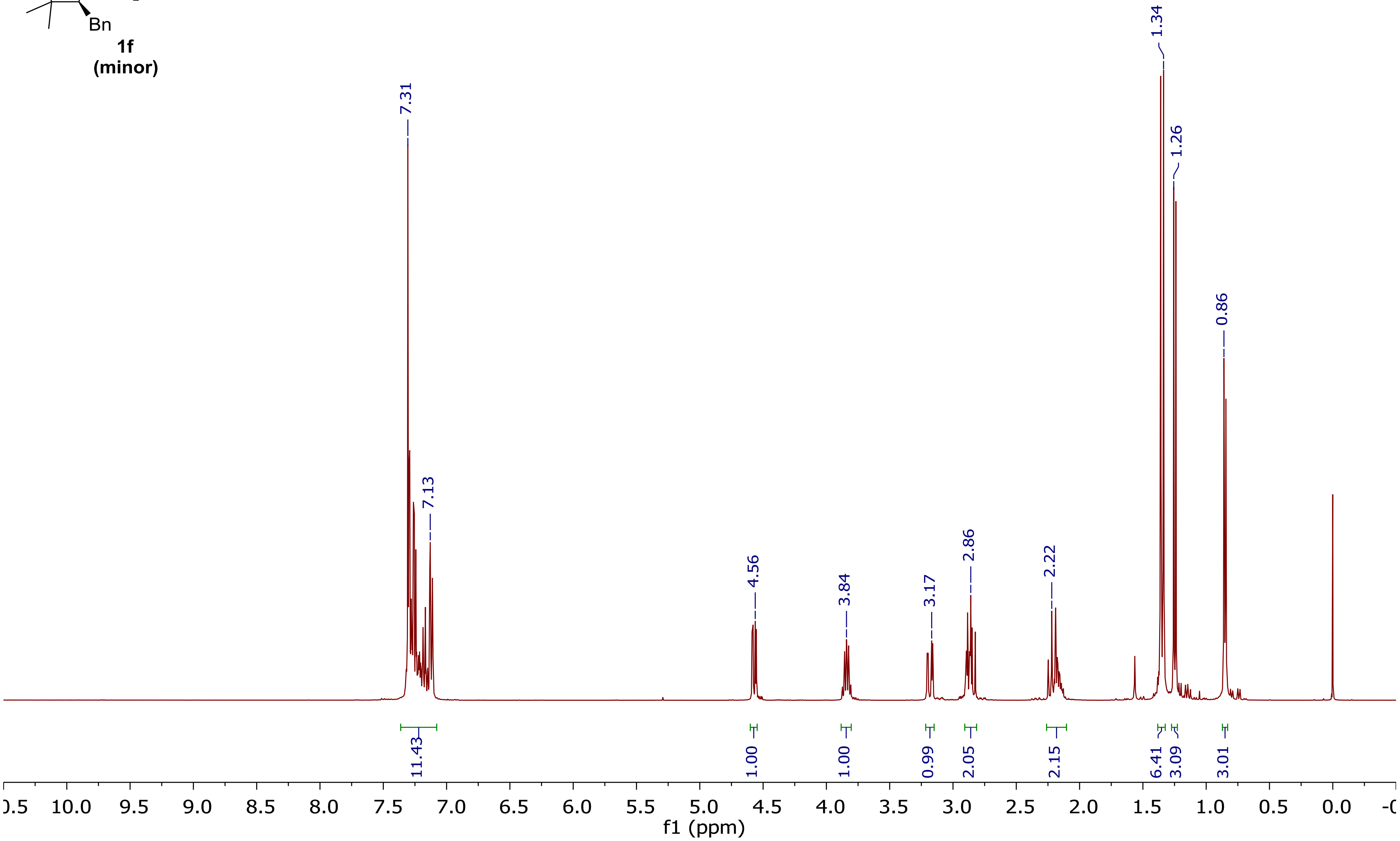

$^{13}C\{^{1}H\}$ NMR (100.6 MHz, $CDCl_3$)

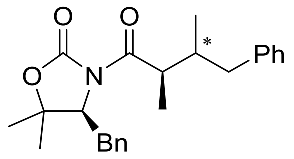

1f
(minor)

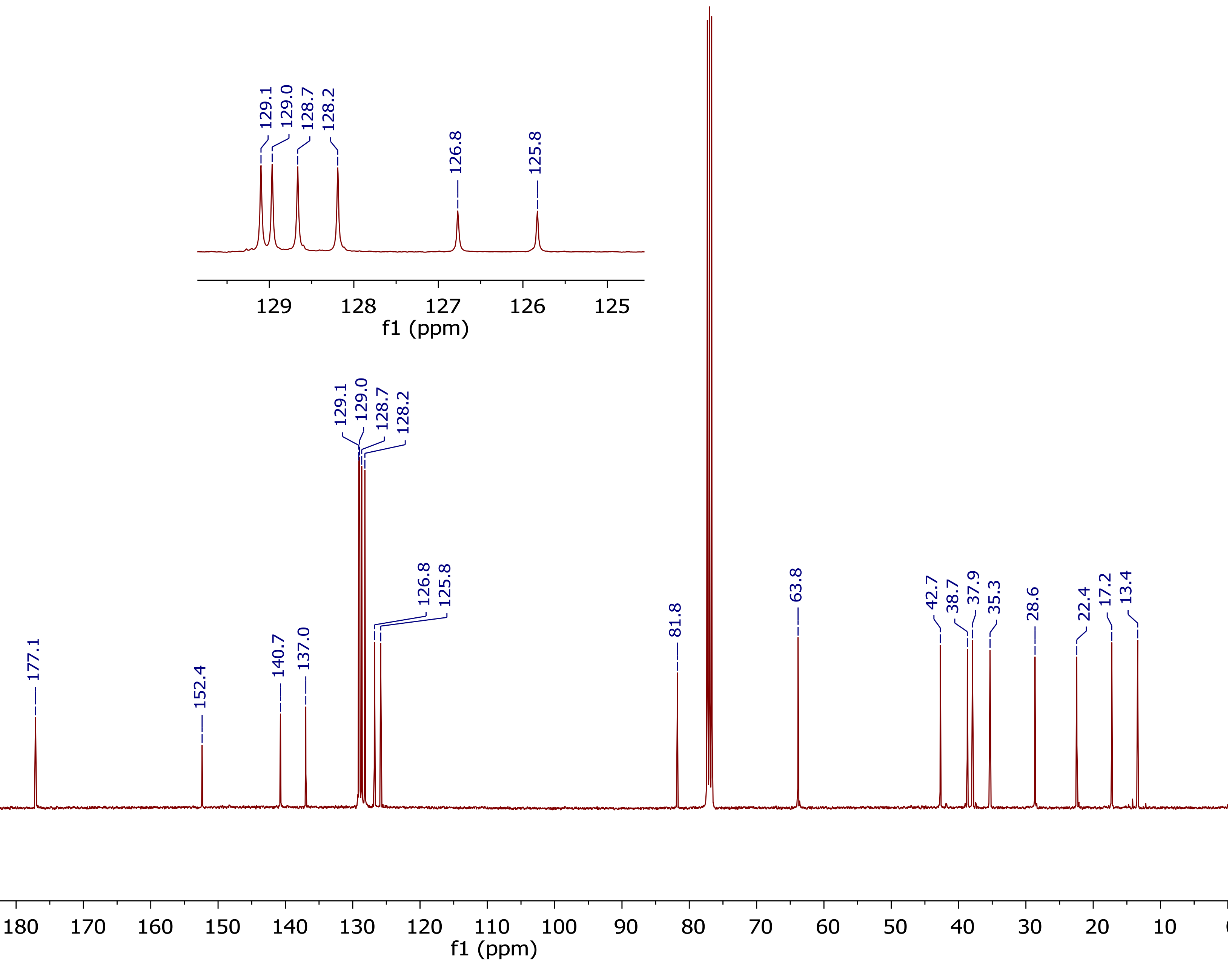

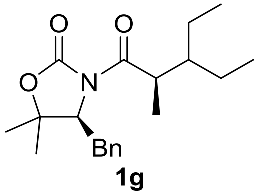

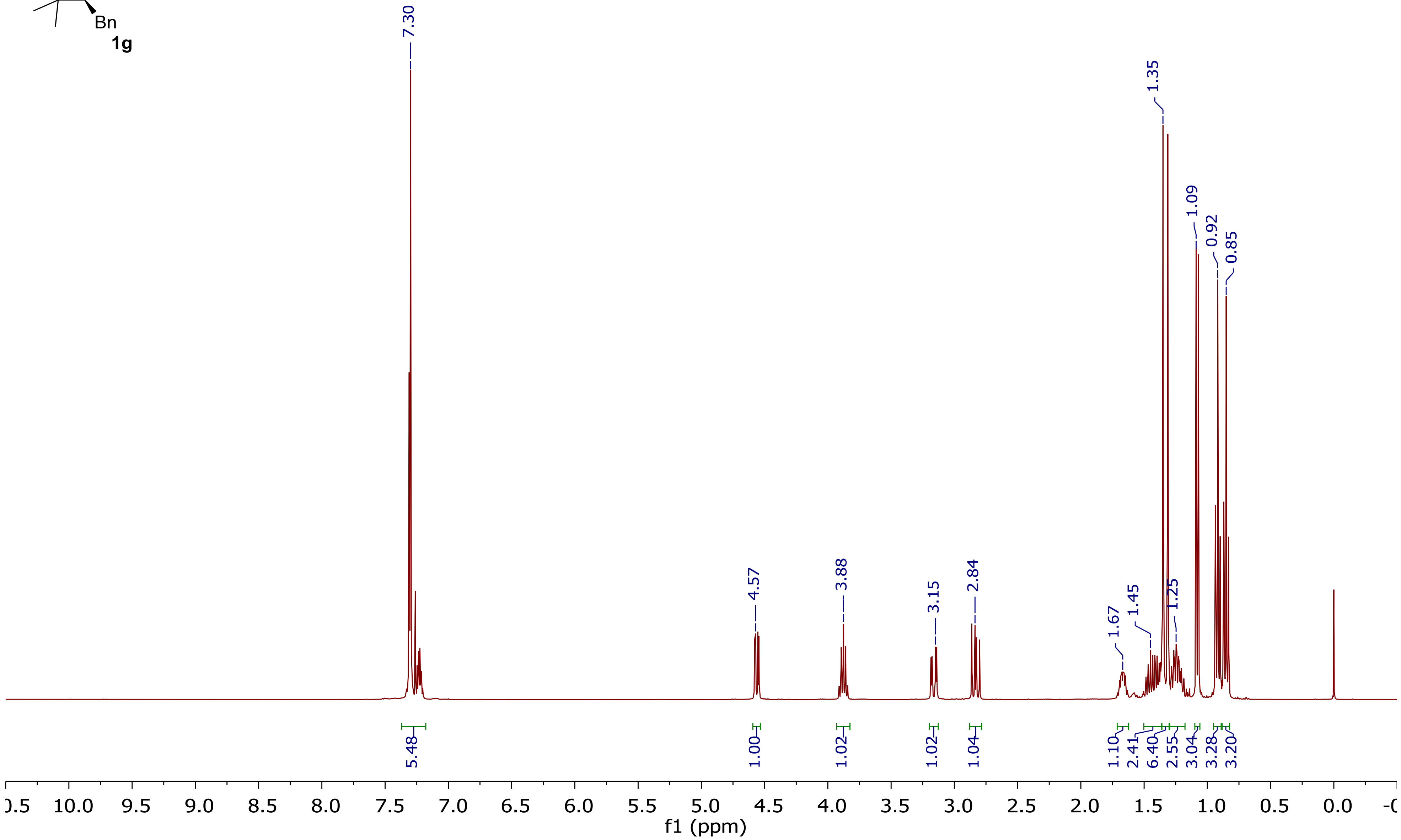

^{1}H NMR (400 MHz, $CDCl_3$)
7.30
1.35
1.09
0.92
0.85
4.57
3.88
3.15
2.84
1.67
1.45
1.25
5.48
1.00
1.02
1.02
1.04
1.10
2.41
6.40
2.55
3.04
3.28
3.20
f1 (ppm)

$^{13}C\{^{1}H\}$ NMR (100.6 MHz, $CDCl_3$)

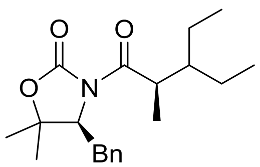

1g

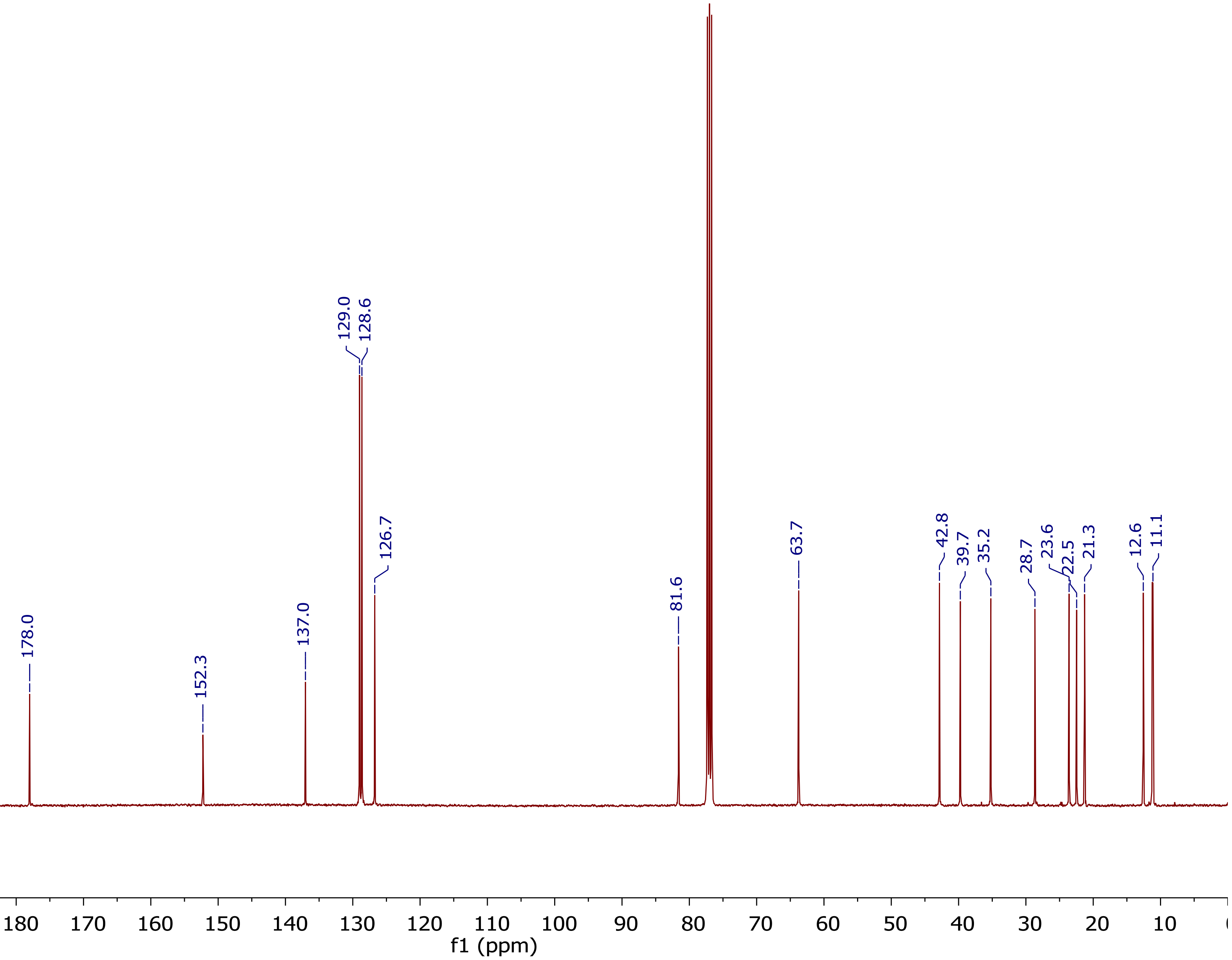

^{1}H NMR (400 MHz, $CDCl_3$)

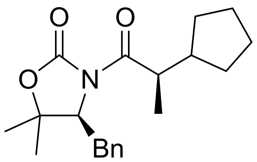

1h

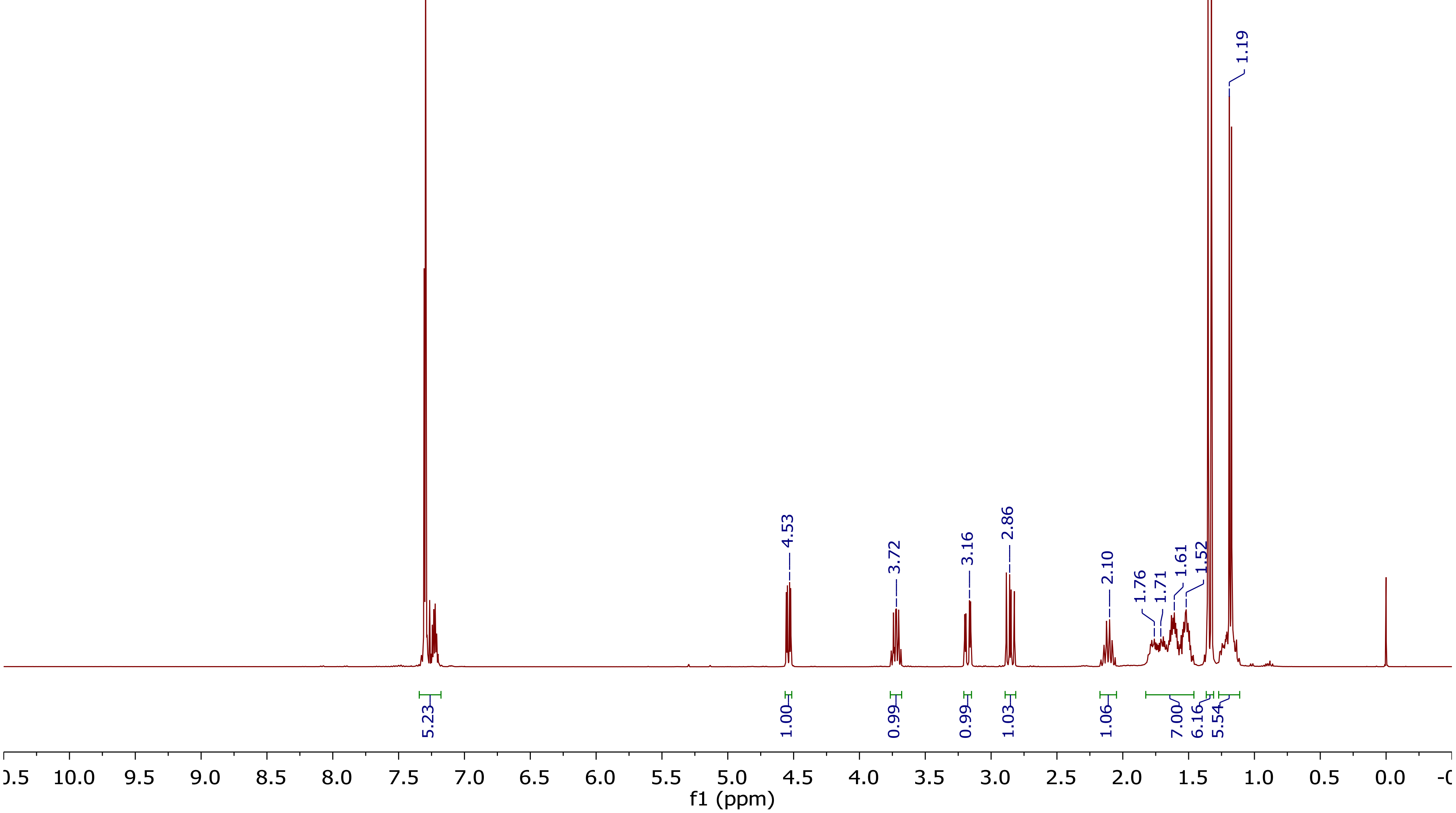

$^{13}C\{^{1}H\}$ NMR (100.6 MHz, CDCl$_3$)

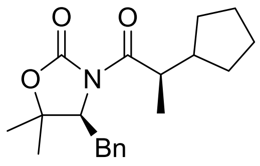

1h

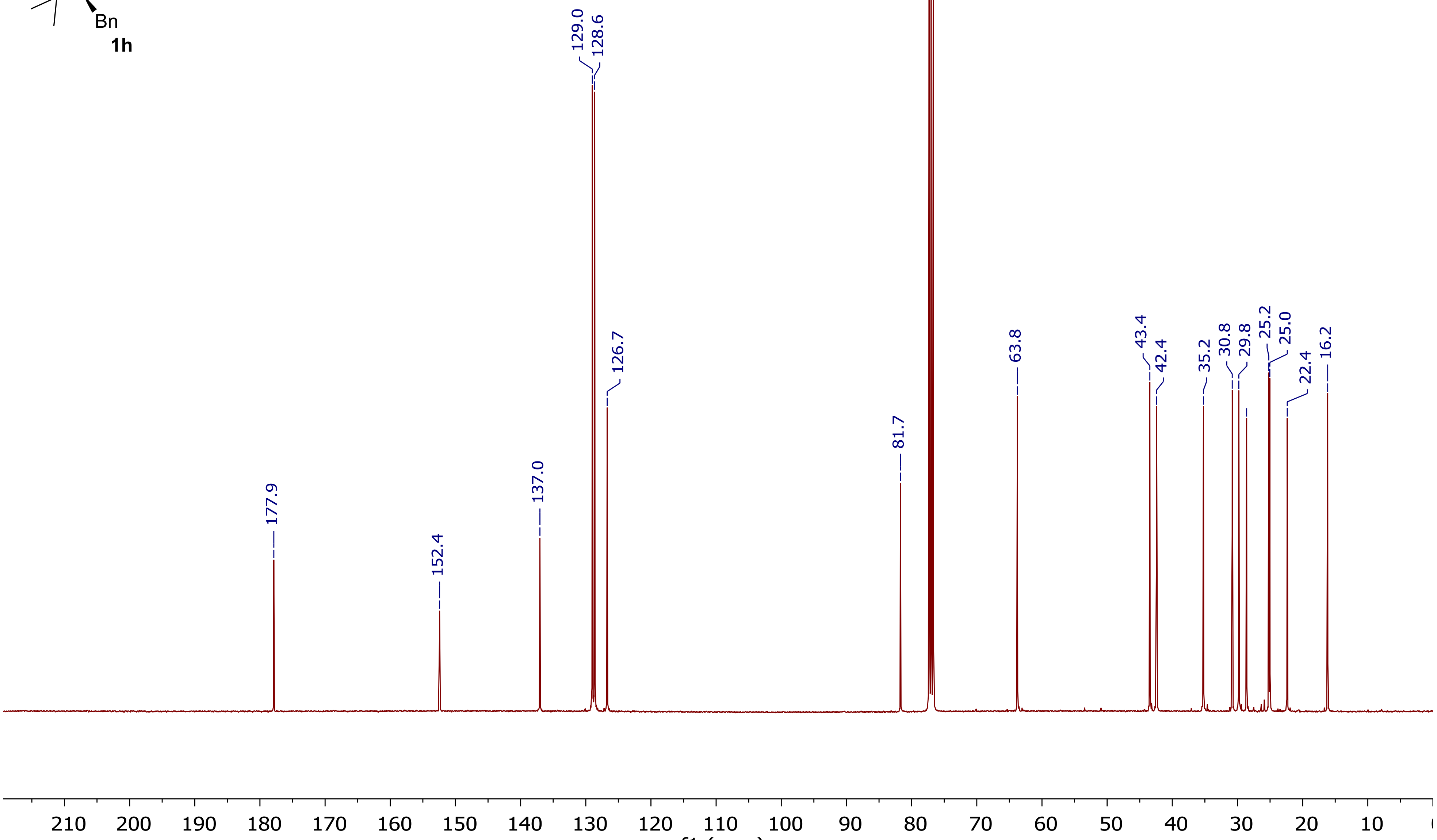

^{1}H NMR (400 MHz, $CDCl_3$)

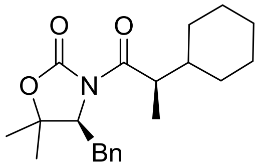

1i

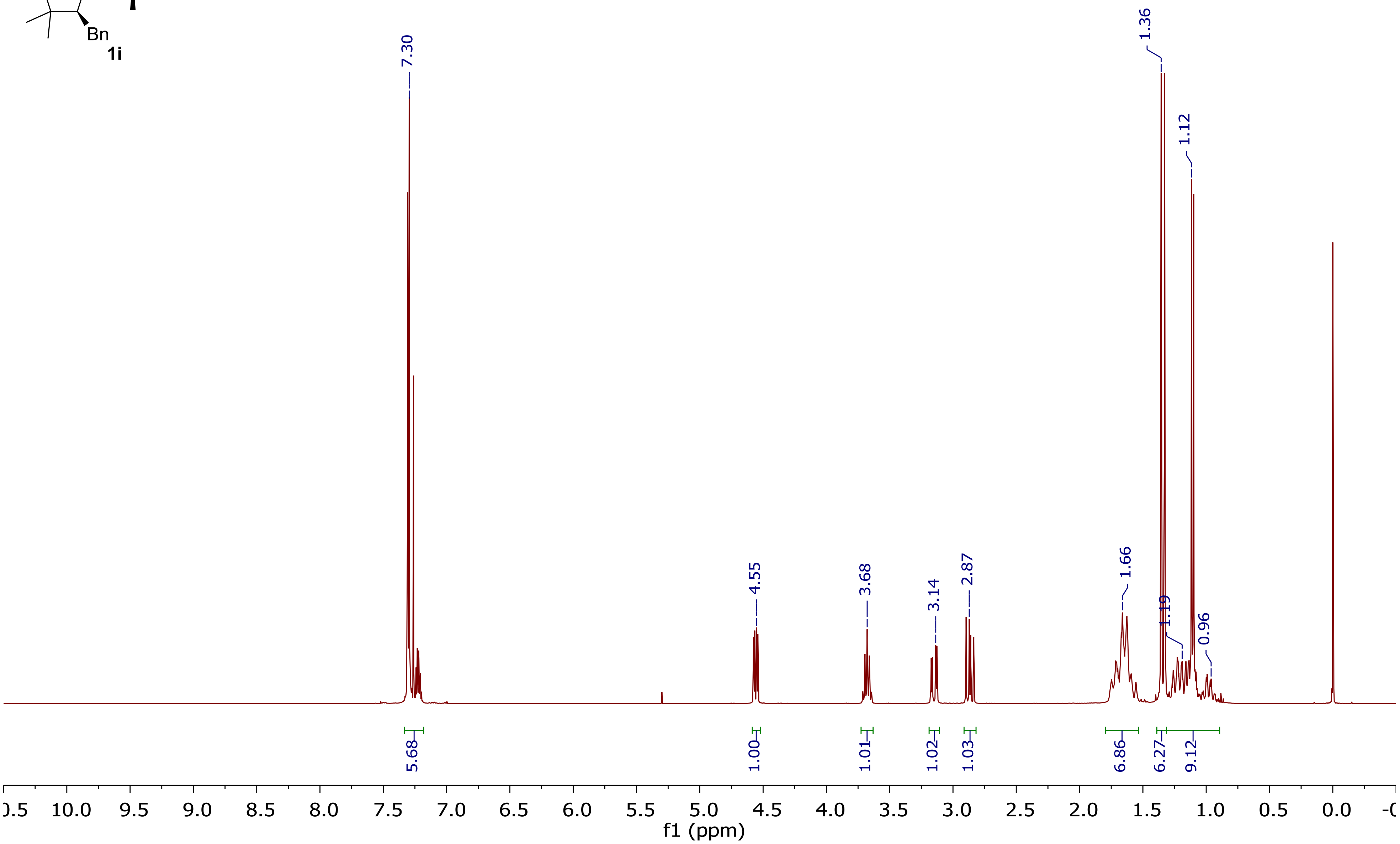

$^{13}C\{^{1}H\}$ NMR (100.6 MHz, $CDCl_3$)

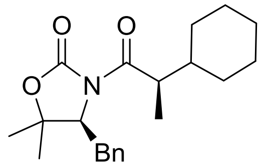

1i

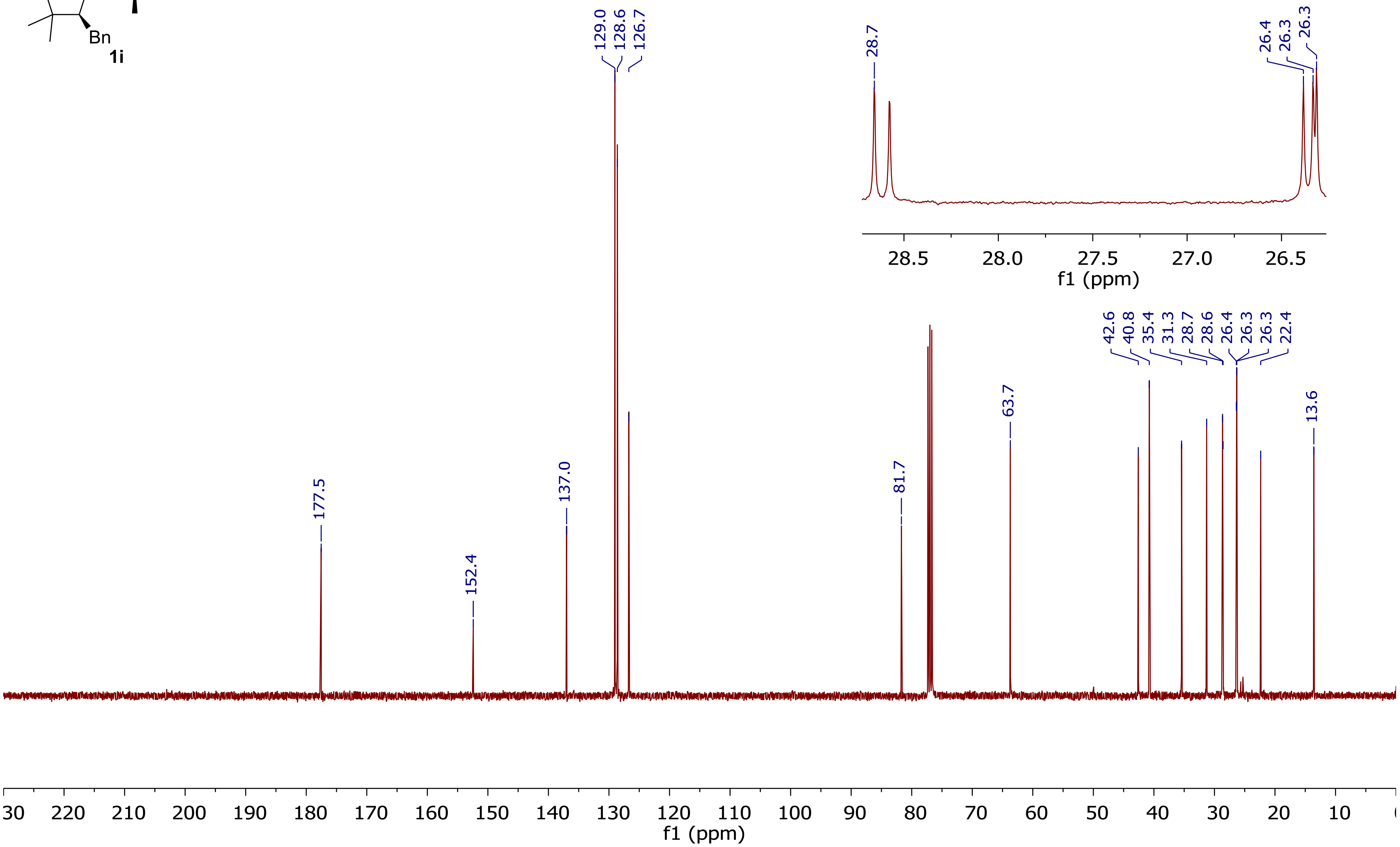

^{1}H NMR (400 MHz, $CDCl_3$)

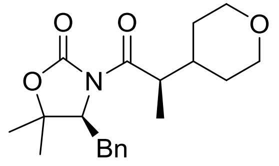

1j

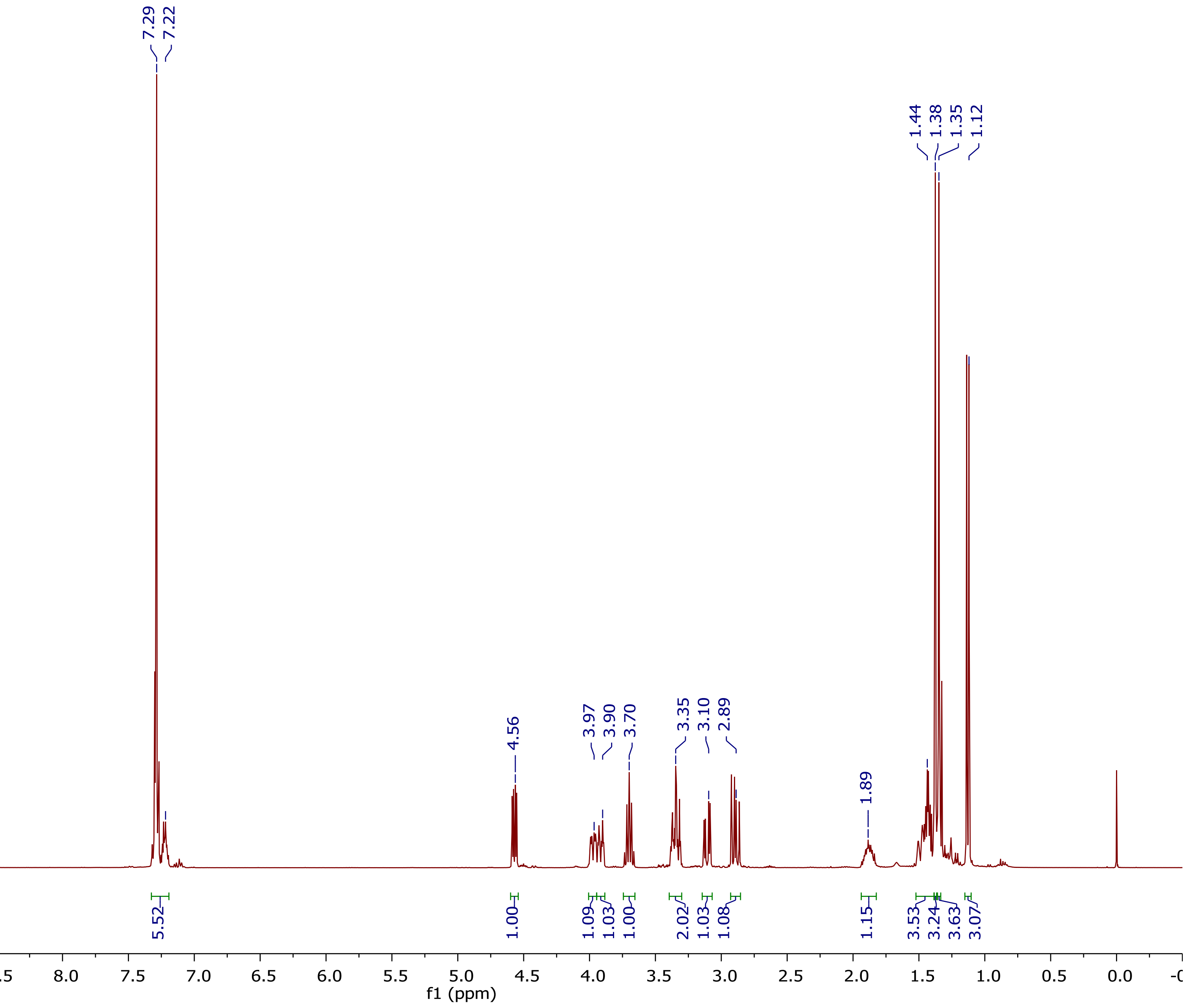

$^{13}C\{^{1}H\}$ NMR (100.6 MHz, $CDCl_3$)

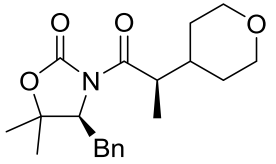

1j

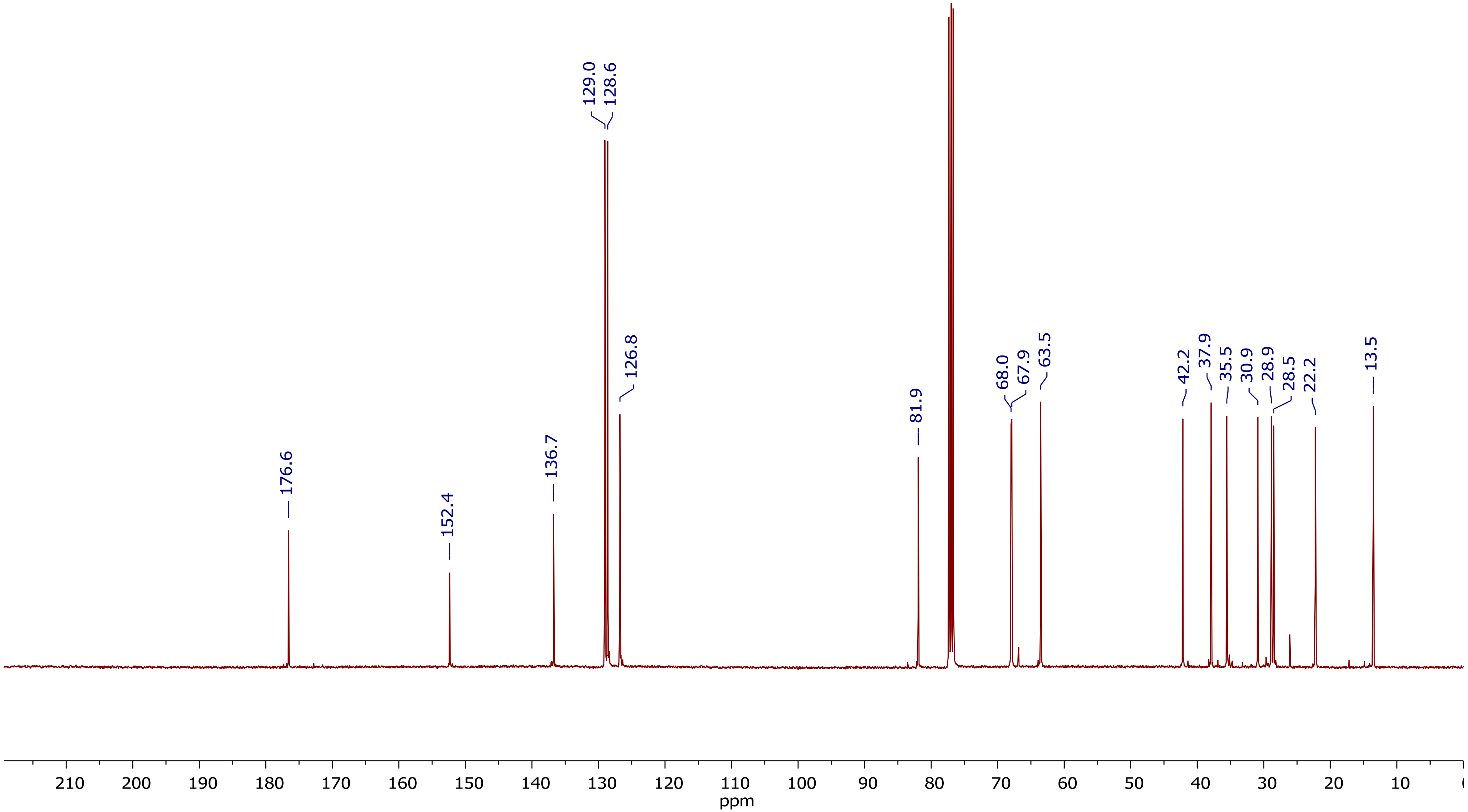

^{1}H NMR (400 MHz, $CDCl_3$)

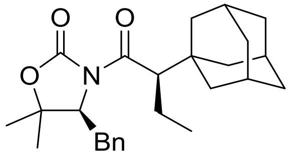

2a

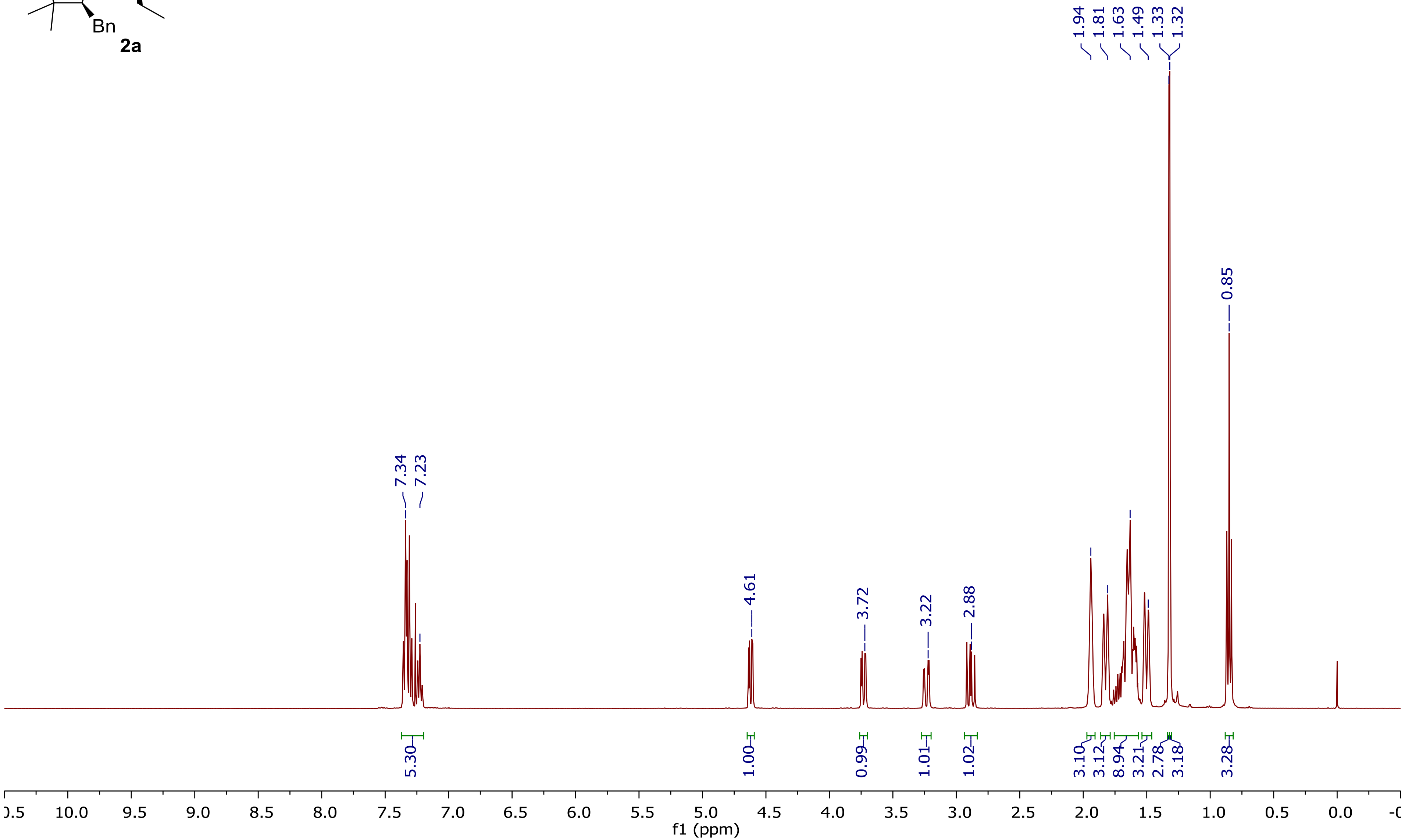

$^{13}C\{^{1}H\}$ NMR (100.6 MHz, $CDCl_3$)

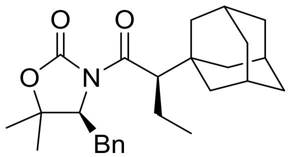

2a

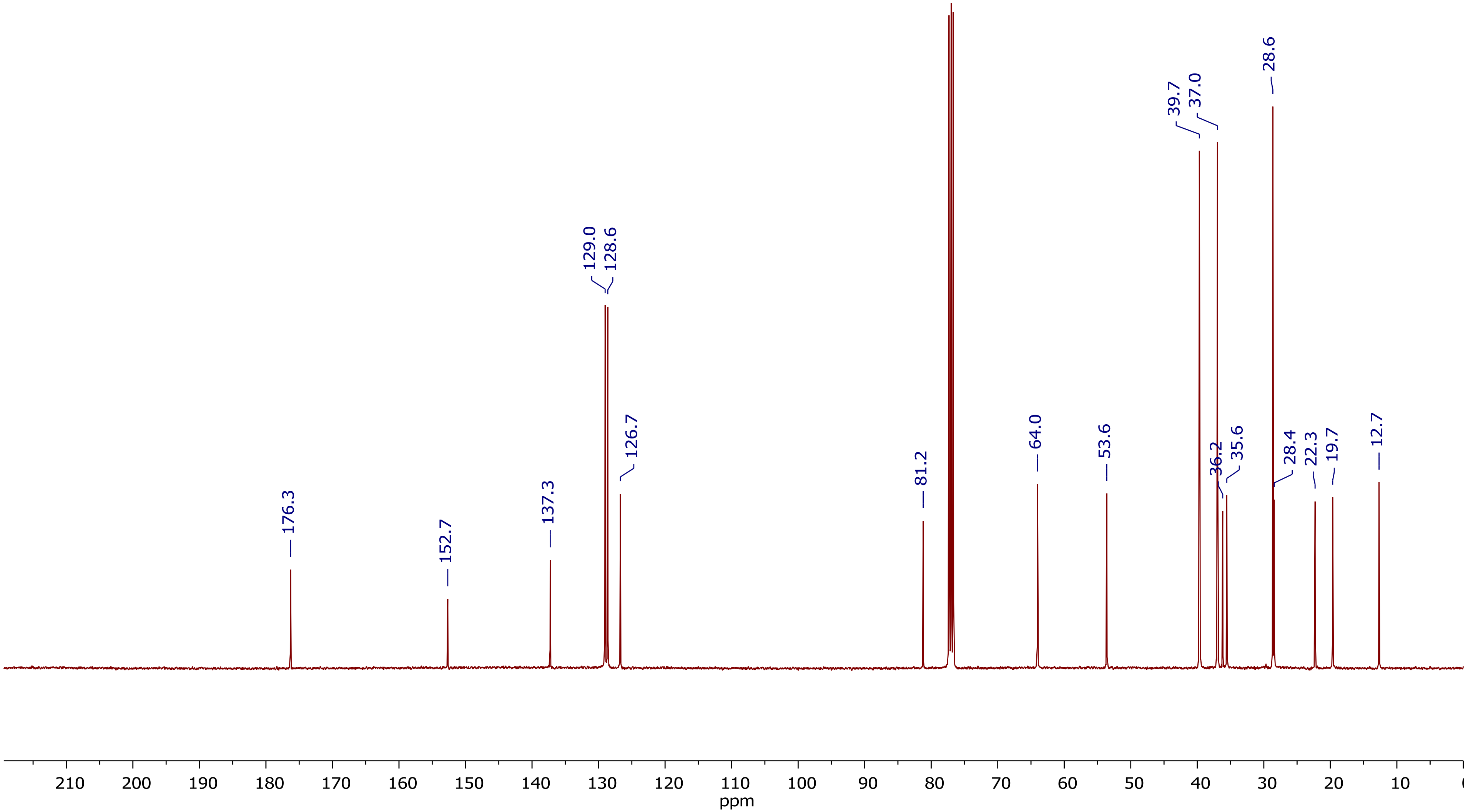

^{1}H NMR (400 MHz, $CDCl_3$)

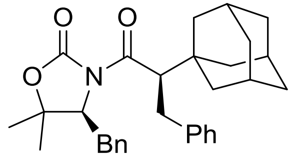

3a

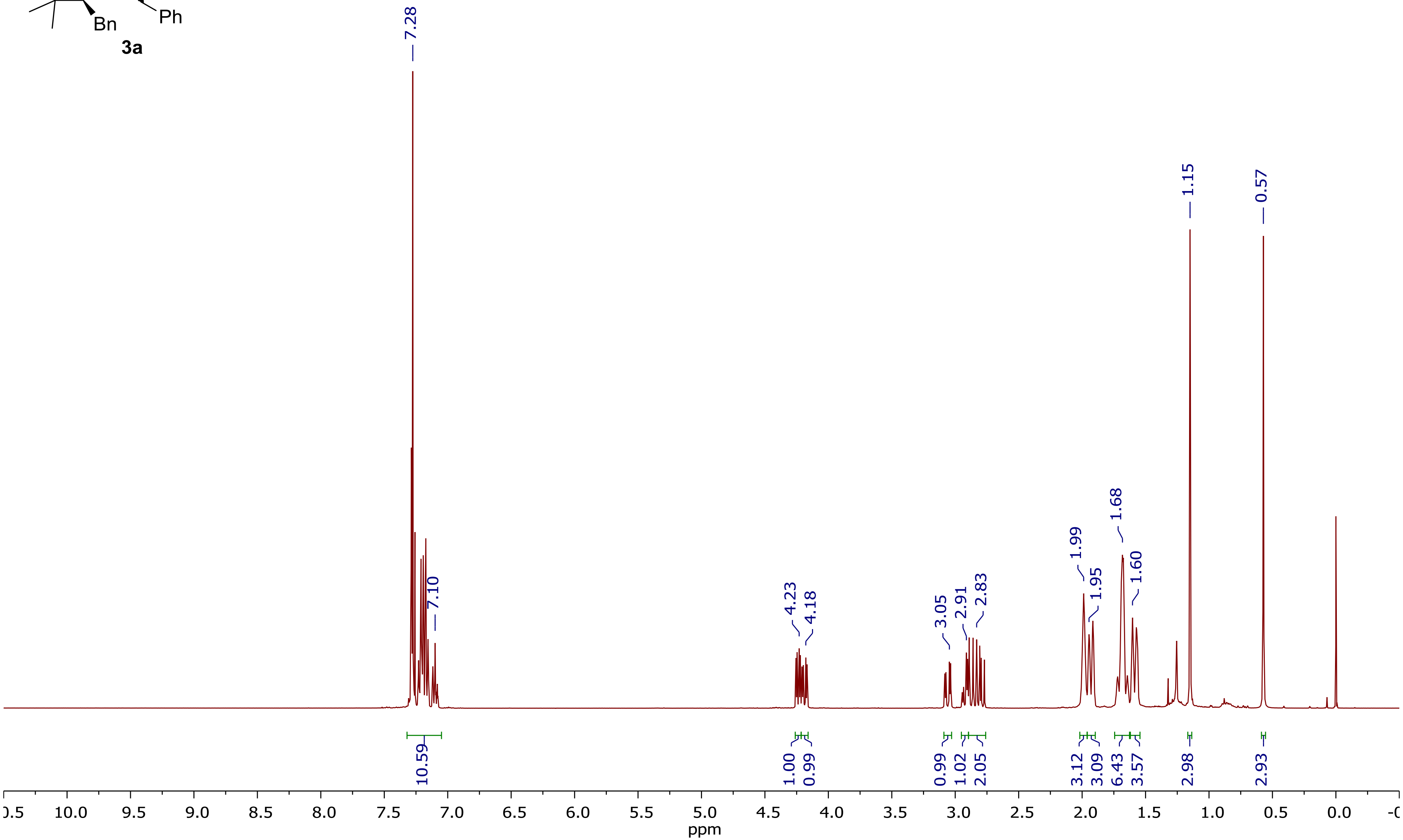

$^{13}C\{^{1}H\}$ NMR (100.6 MHz, $CDCl_3$)

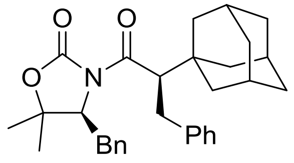

3a

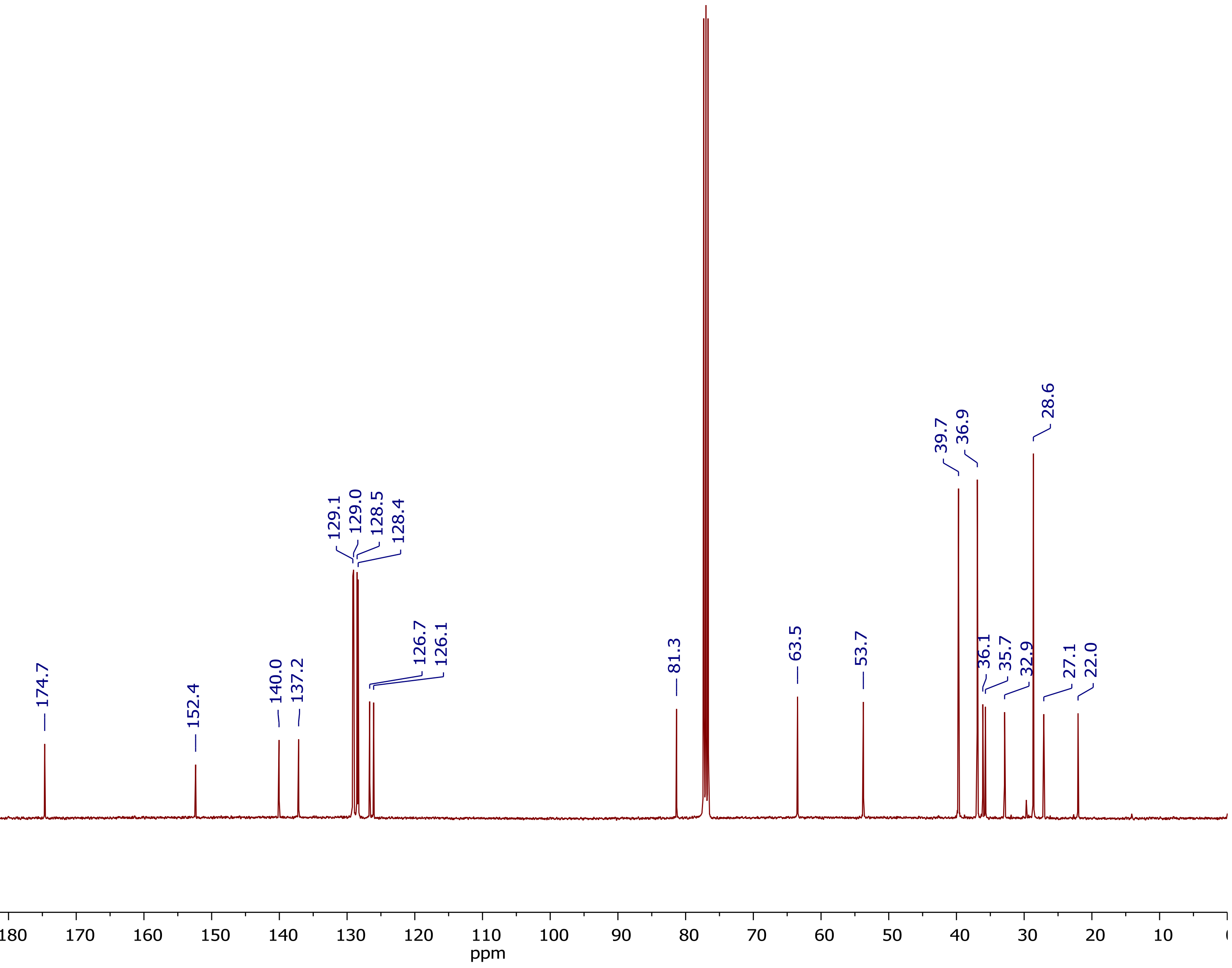

^1H NMR (400 MHz, $CDCl_3$)

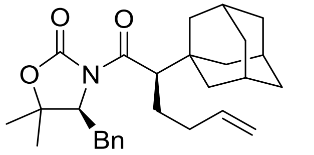

4a

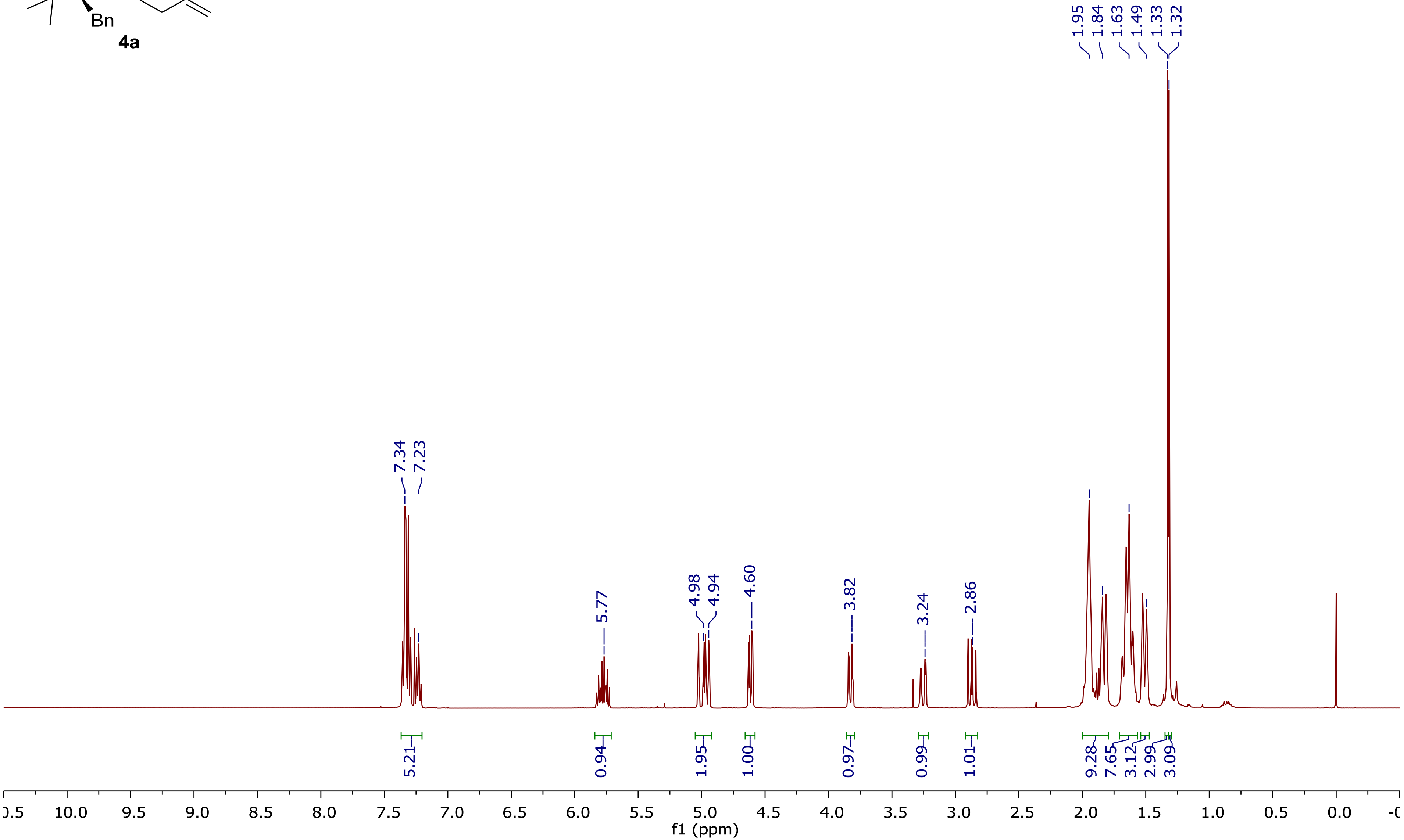

$^{13}C\{^{1}H\}$ NMR (100.6 MHz, $CDCl_3$)

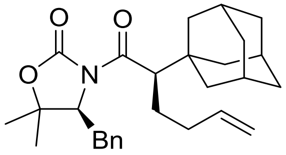

4a

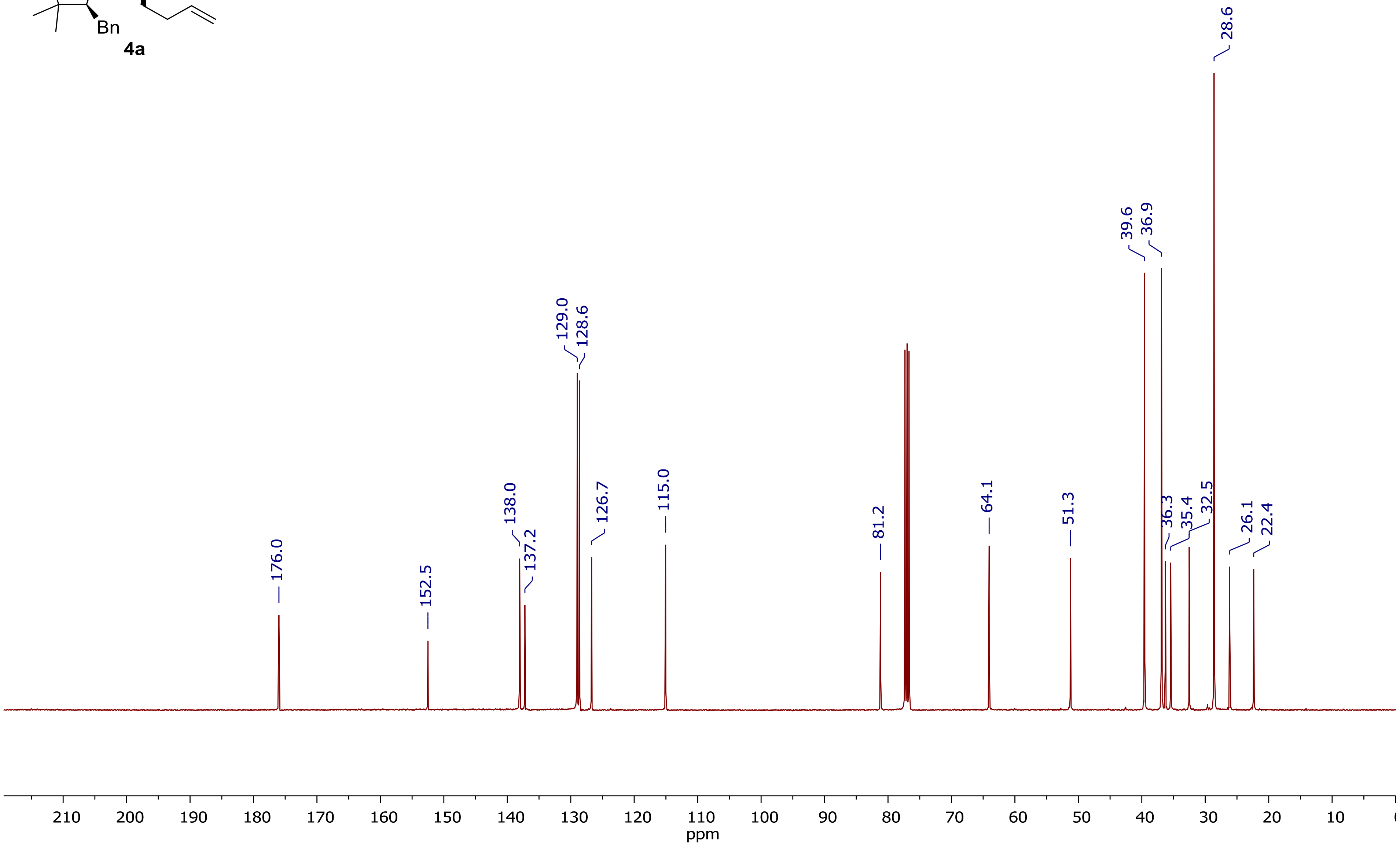

^1H NMR (400 MHz, $CDCl_3$)

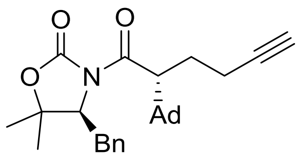

5a

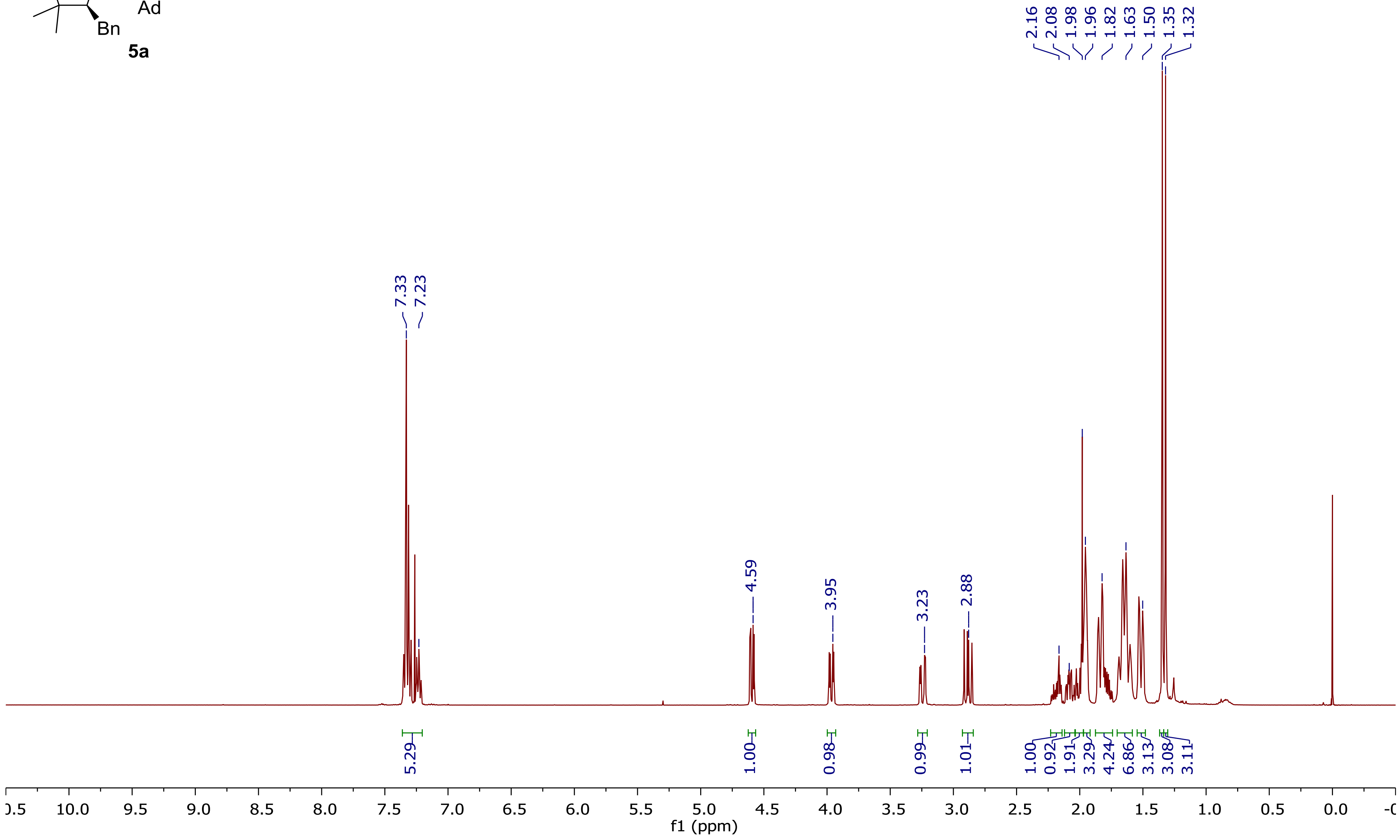

$^{13}C\{^{1}H\}$ NMR (100.6 MHz, $CDCl_3$)

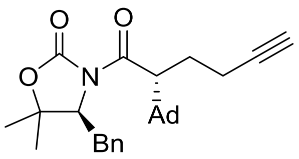

5a

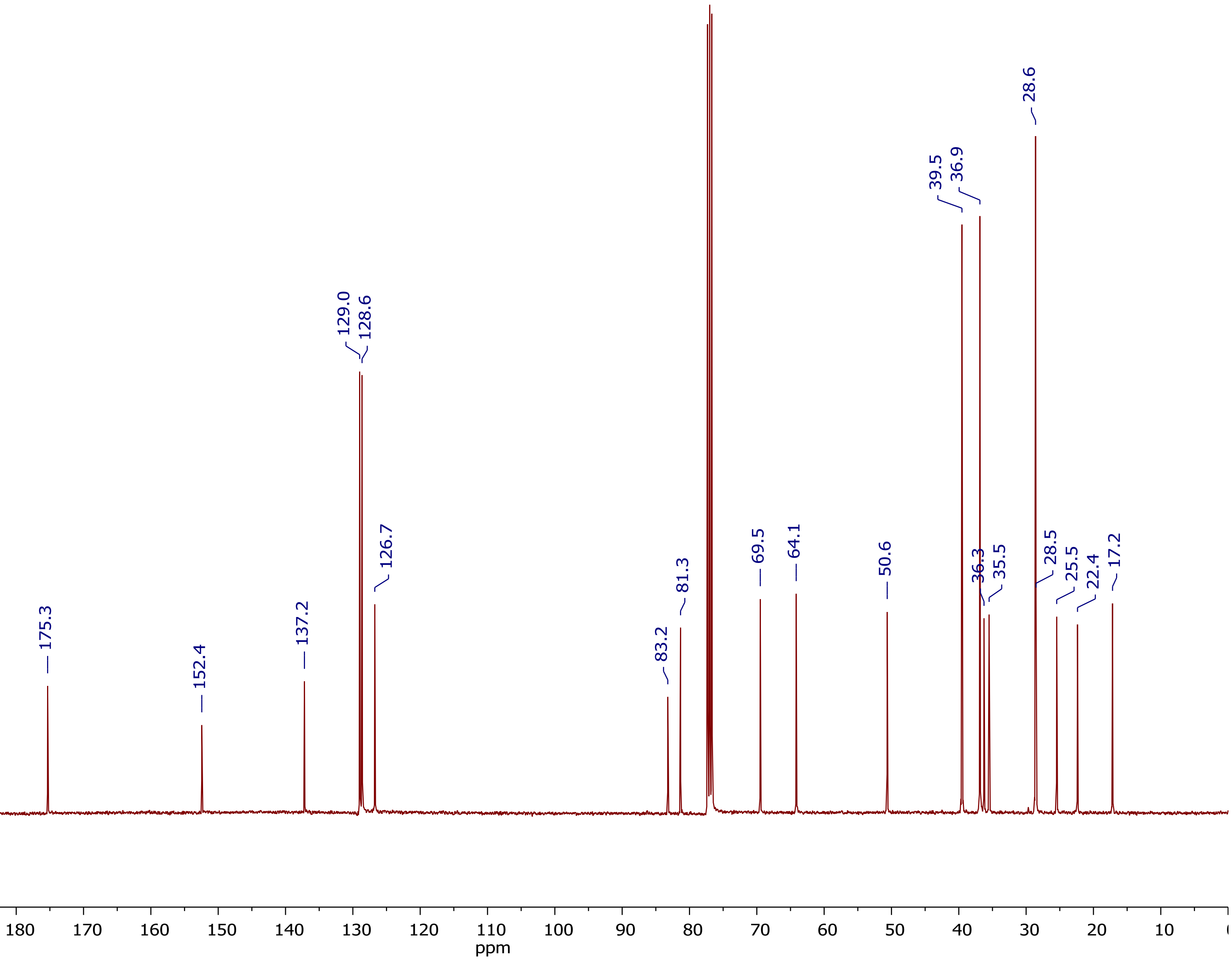

^{1}H NMR (400 MHz, $CDCl_3$)

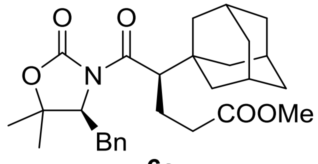

6a

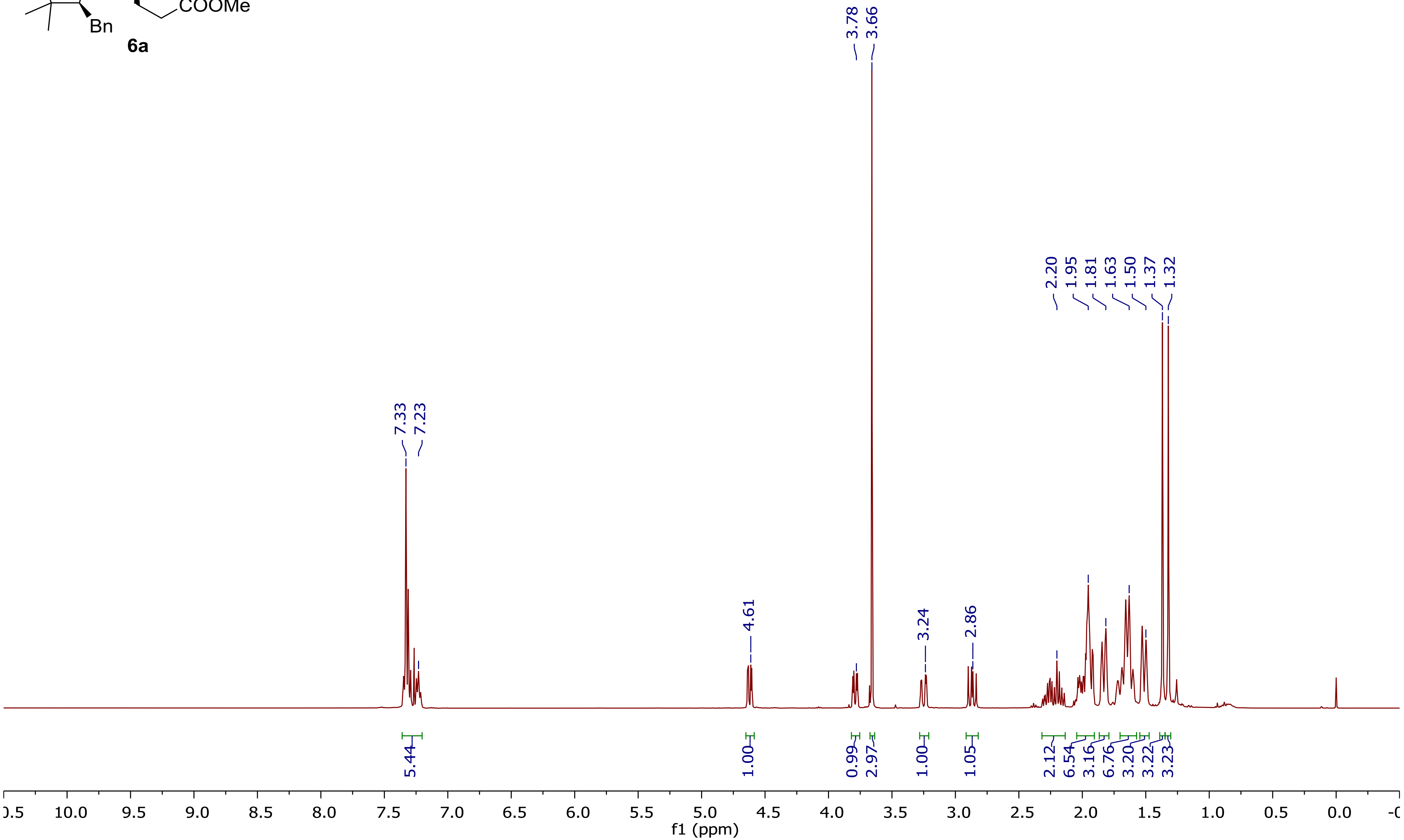

$^{13}C\{^{1}H\}$ NMR (100.6 MHz, $CDCl_3$)

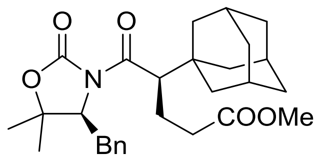

6a

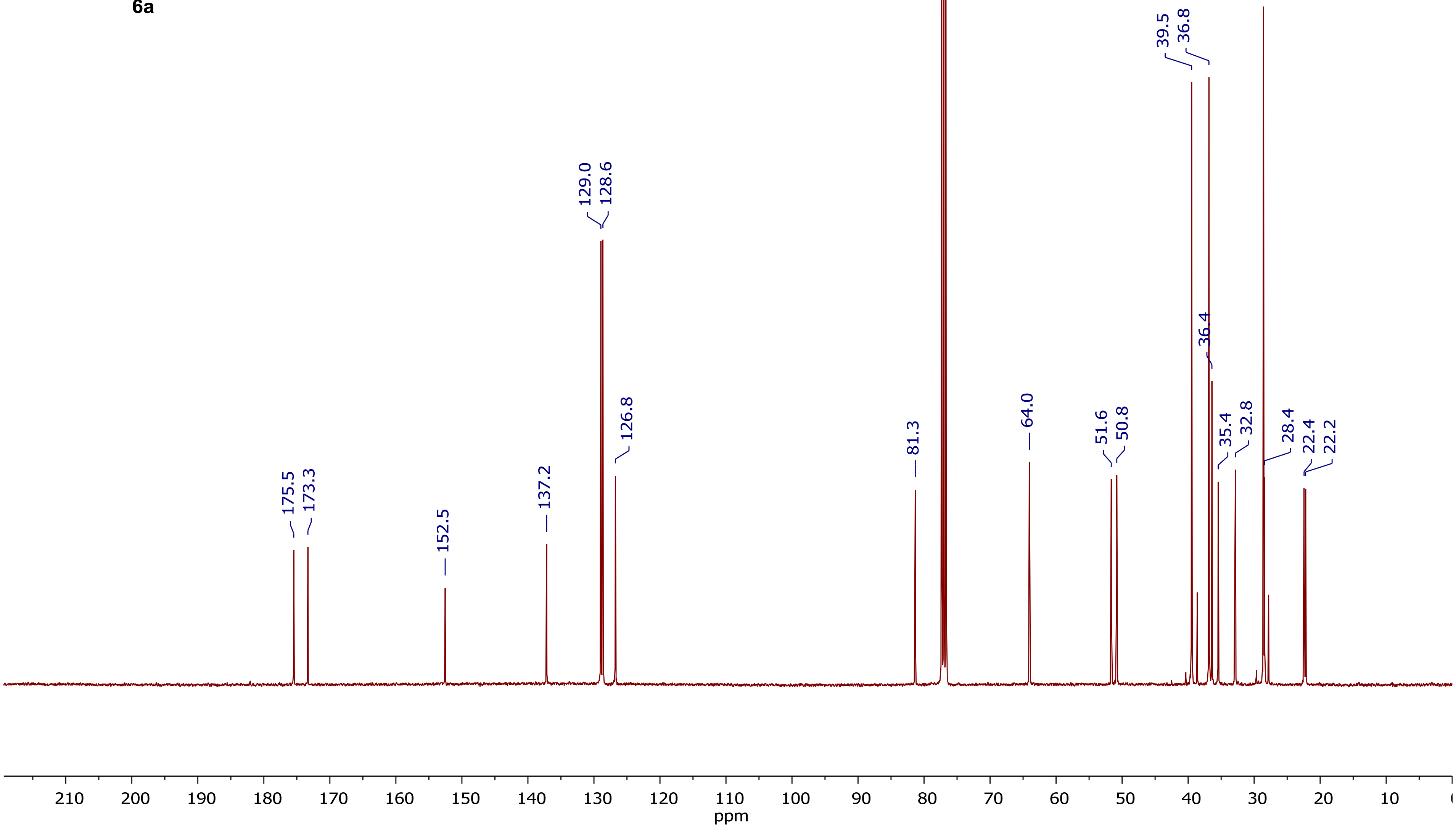

^{1}H NMR (400 MHz, $CDCl_3$)

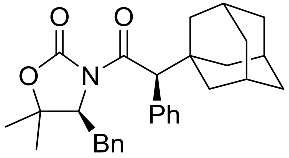

7a

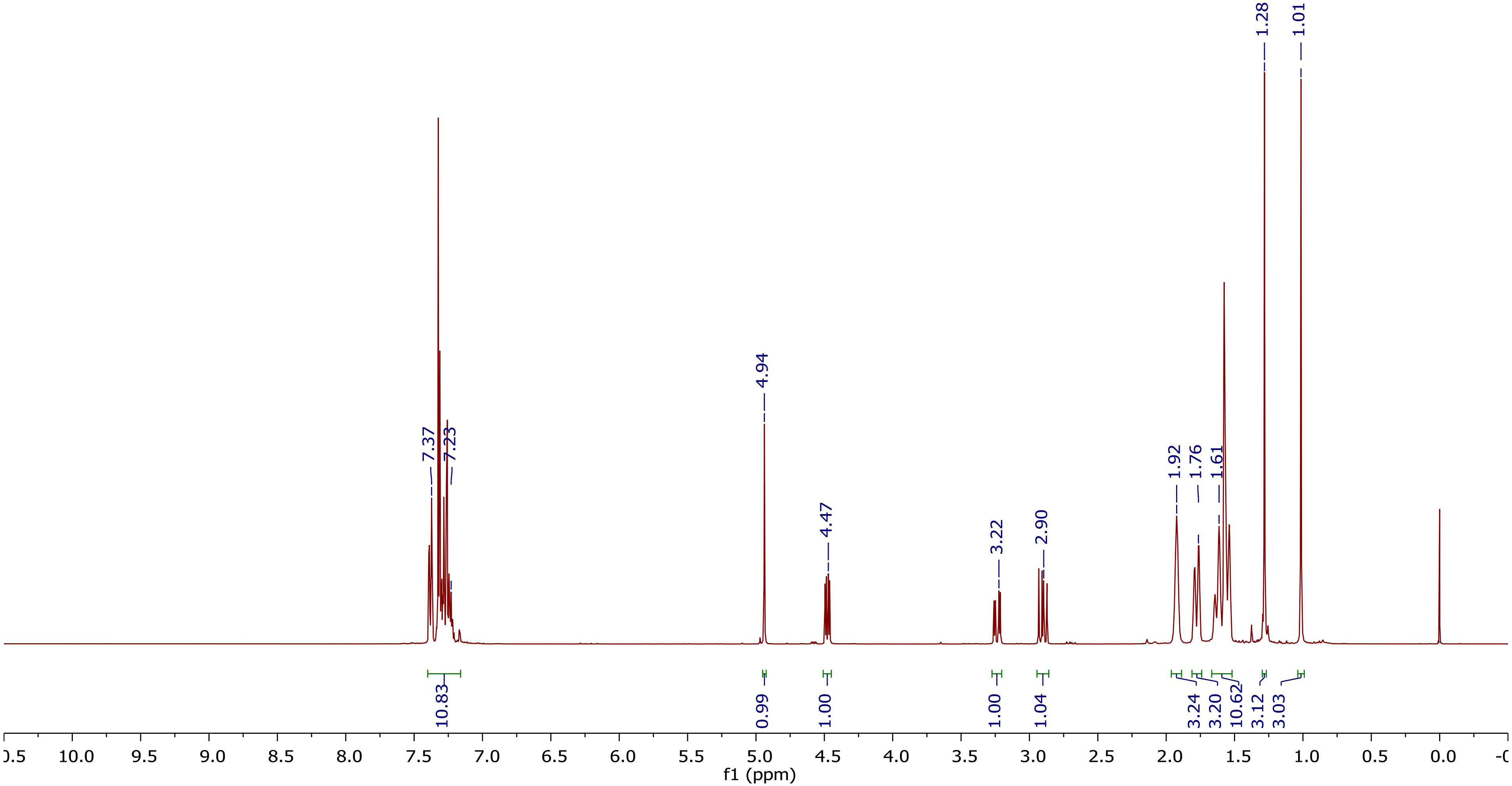

$^{13}C\{^{1}H\}$ NMR (100.6 MHz, $CDCl_3$)

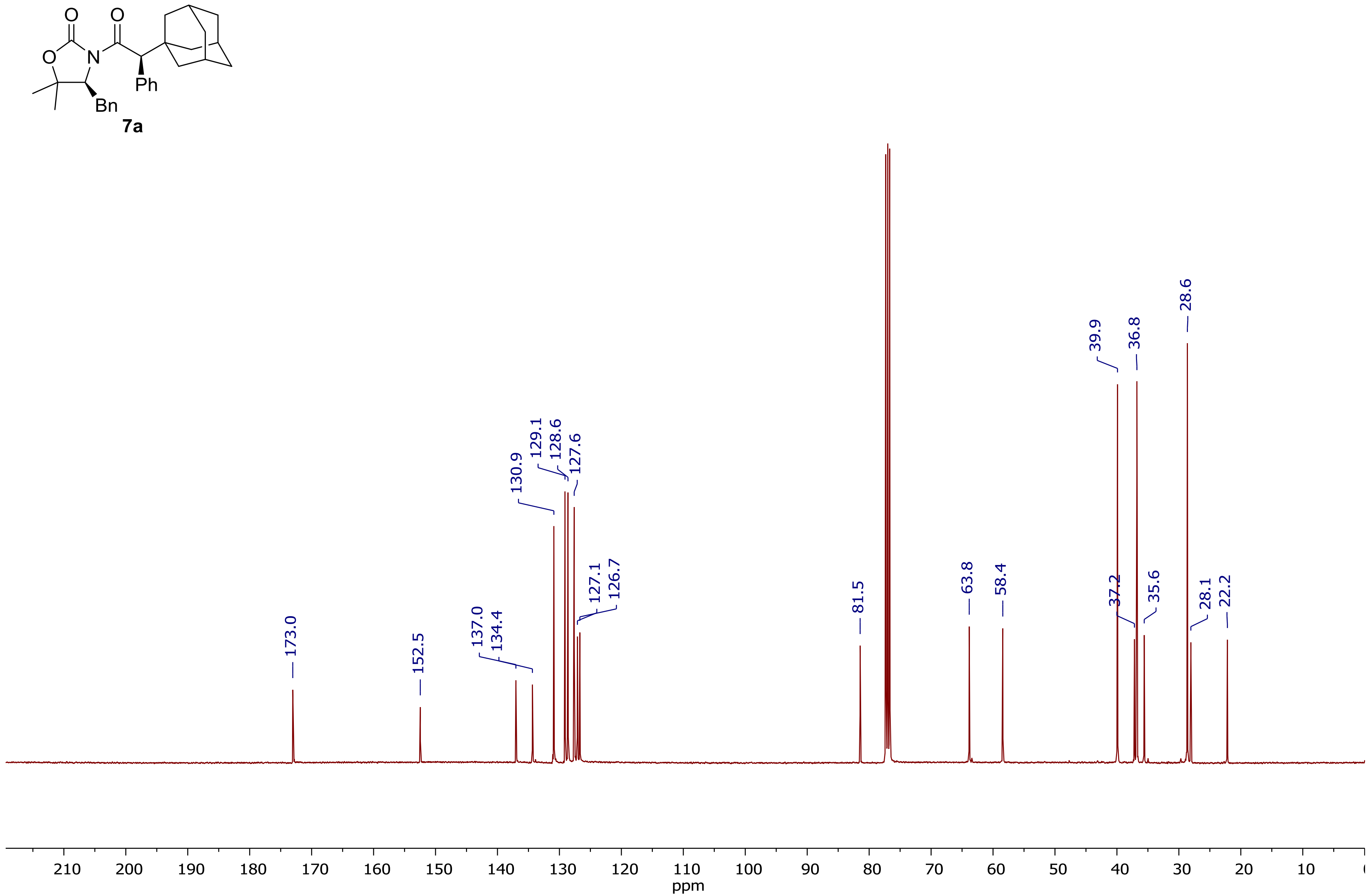

^{1}H NMR (400 MHz, $CDCl_3$)

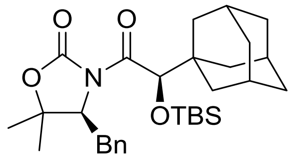

8a

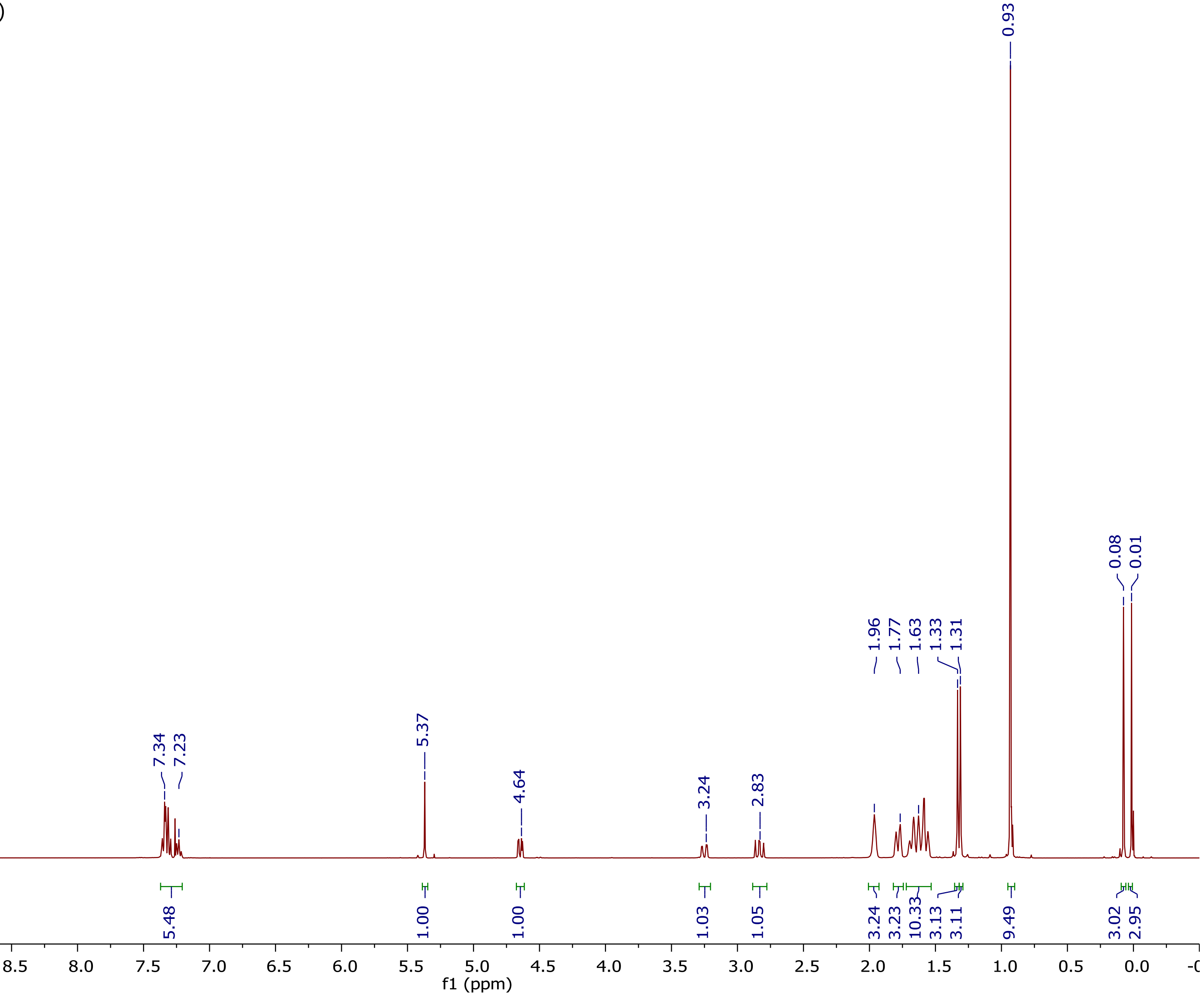

$^{13}C\{^{1}H\}$ NMR (100.6 MHz, $CDCl_3$)

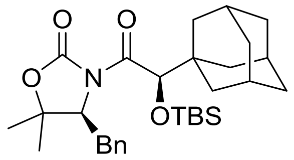

8a

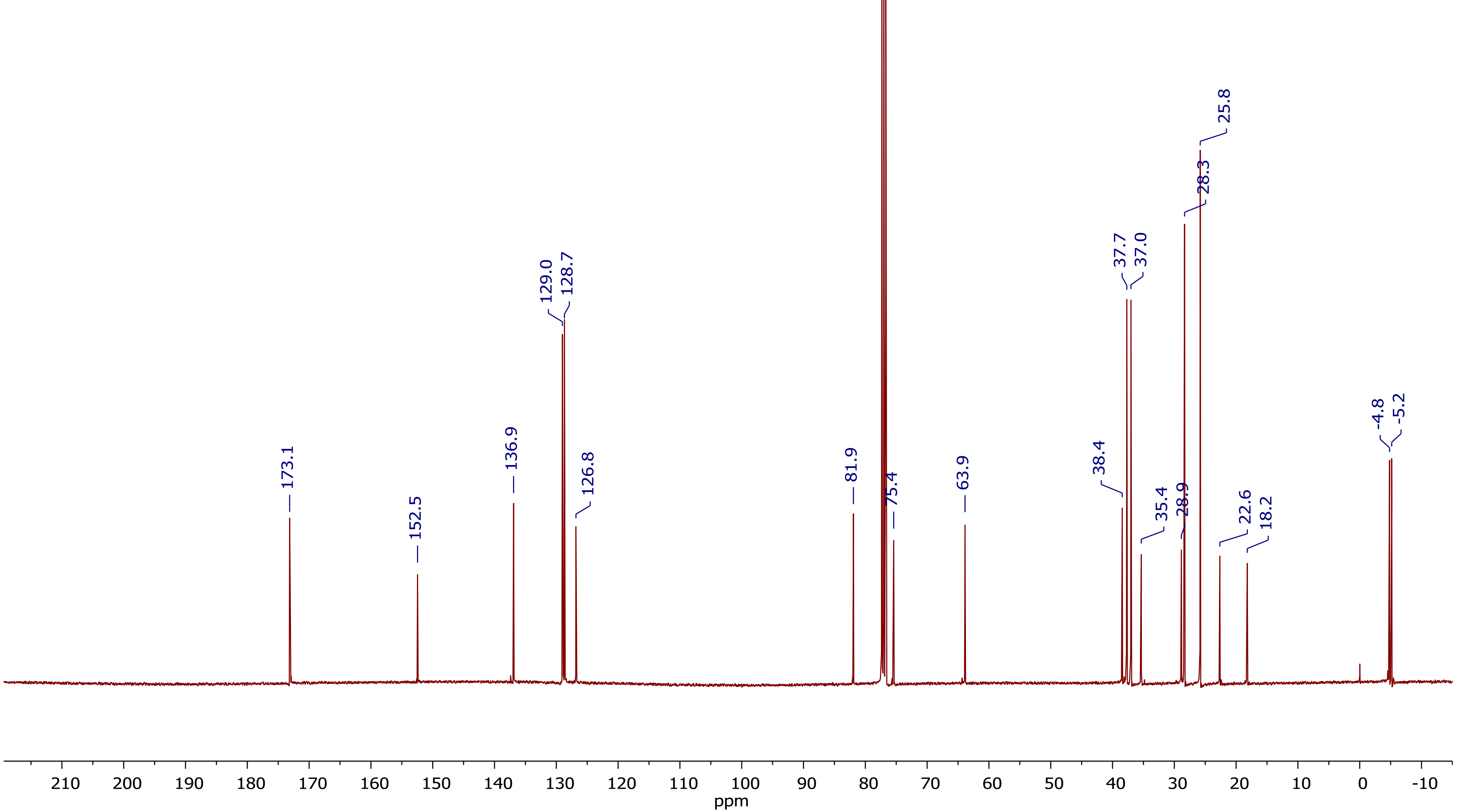

^{1}H NMR (400 MHz, $CDCl_3$)

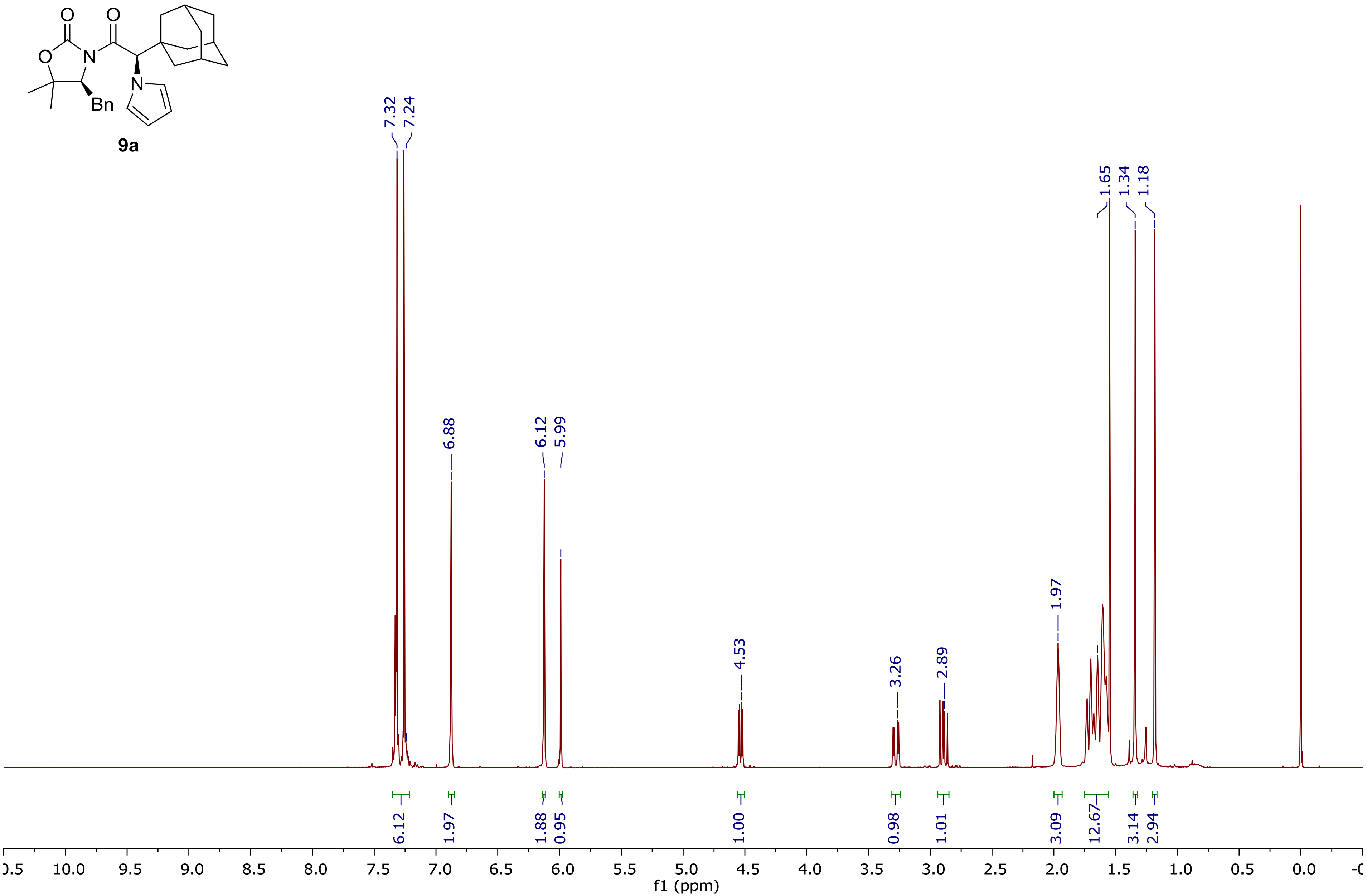

$^{13}C\{^{1}H\}$ NMR (100.6 MHz, $CDCl_3$)

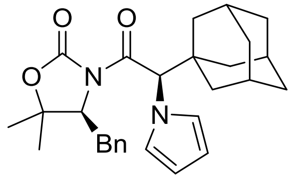

9a

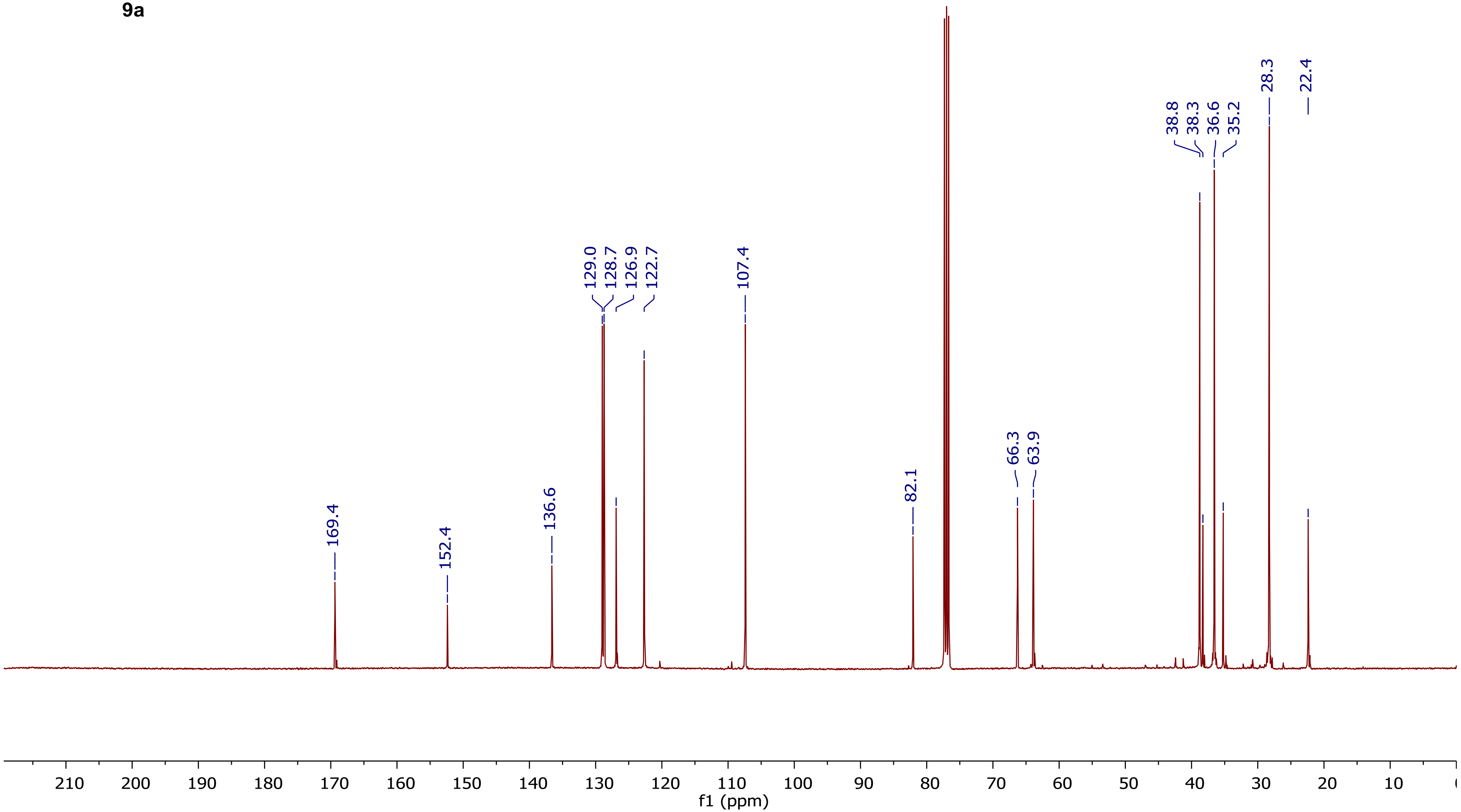

^{1}H NMR (400 MHz, $CDCl_3$)

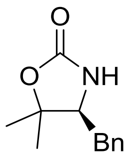

SI1

Recovered after acylation, alkylation and removal of the chiral auxiliary

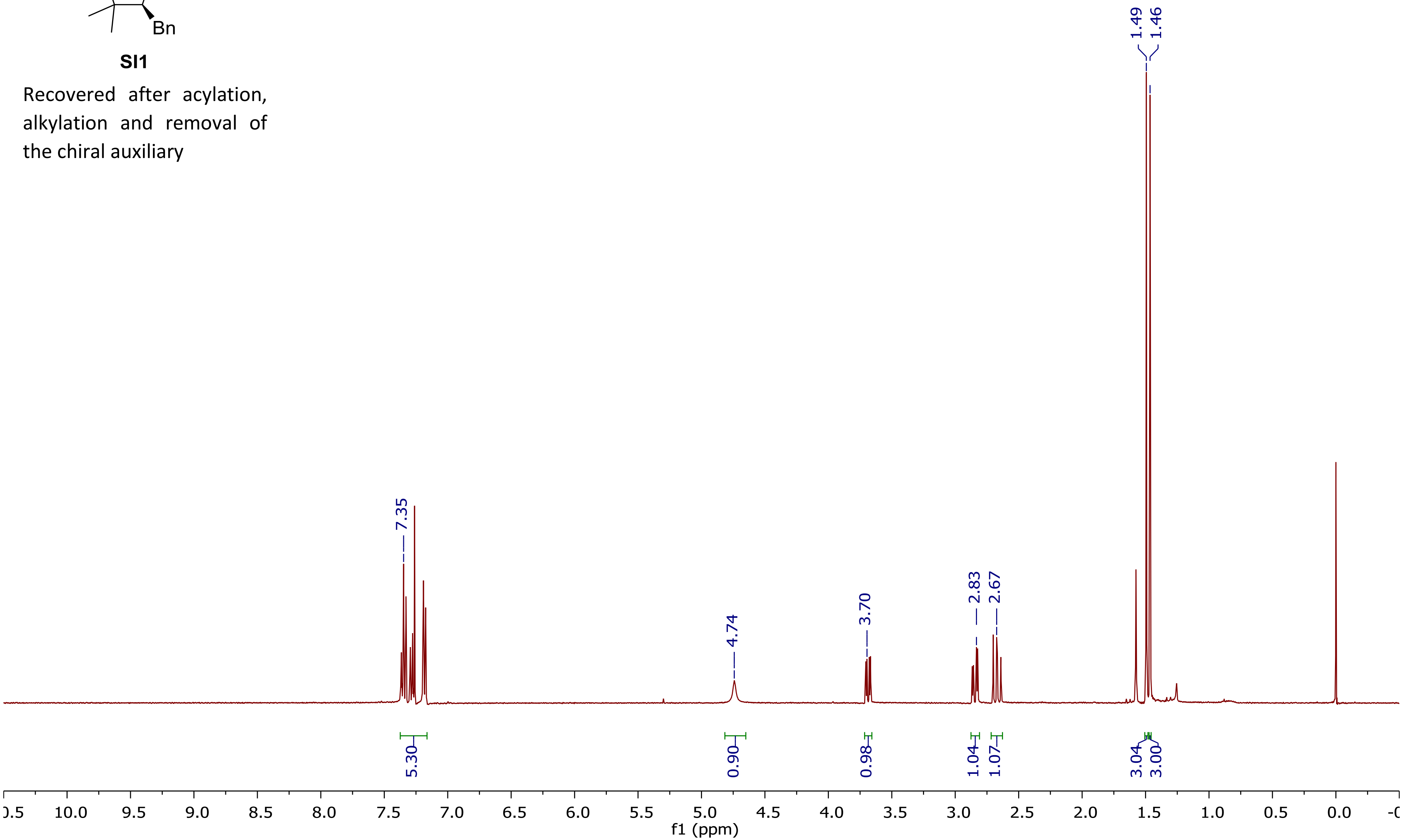

^{1}H NMR (400 MHz, $CDCl_3$)

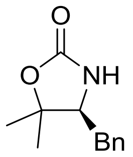

SI1

Prepared according to literature[1]

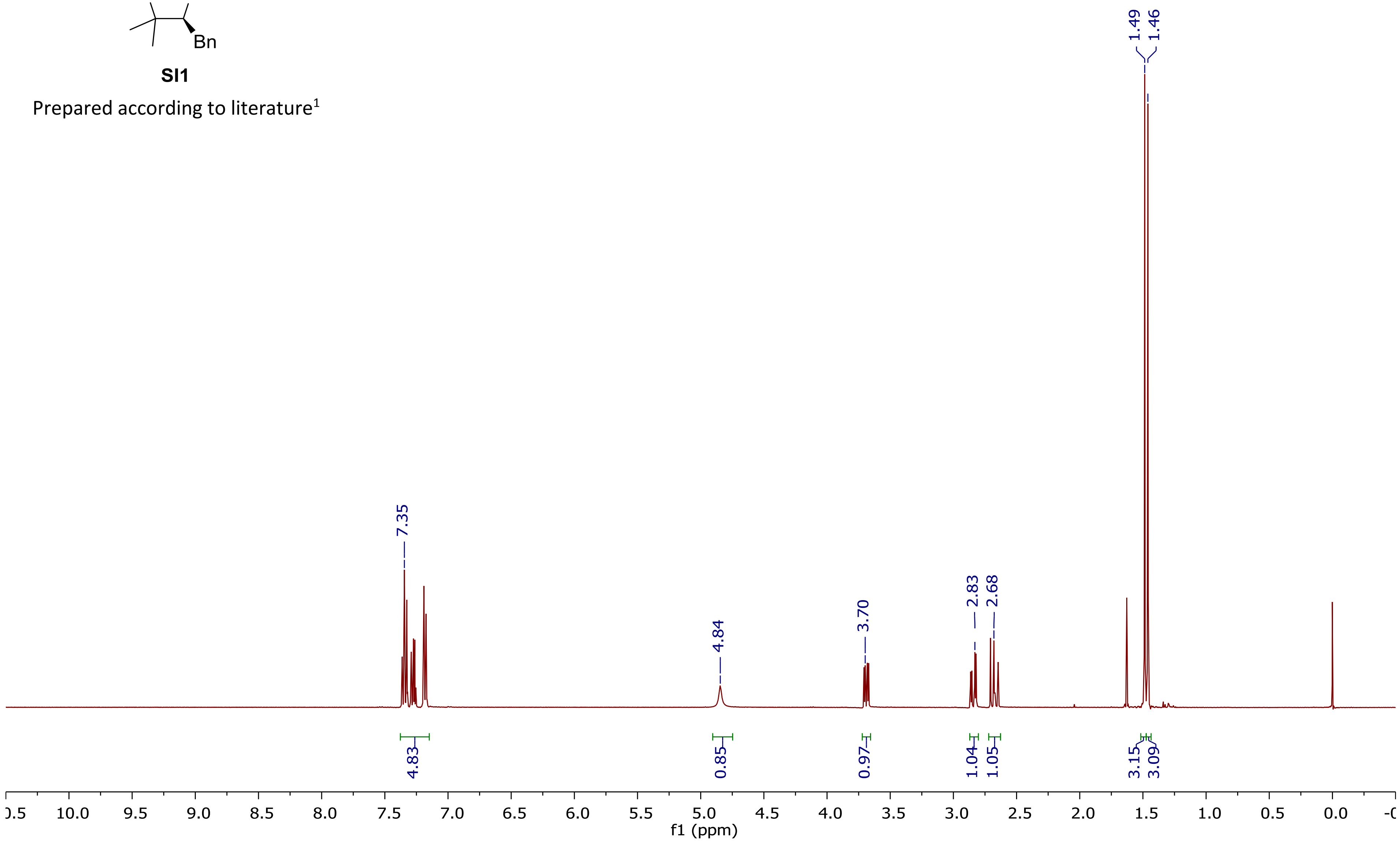

^{1}H NMR (400 MHz, $CDCl_3$)

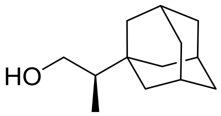

10

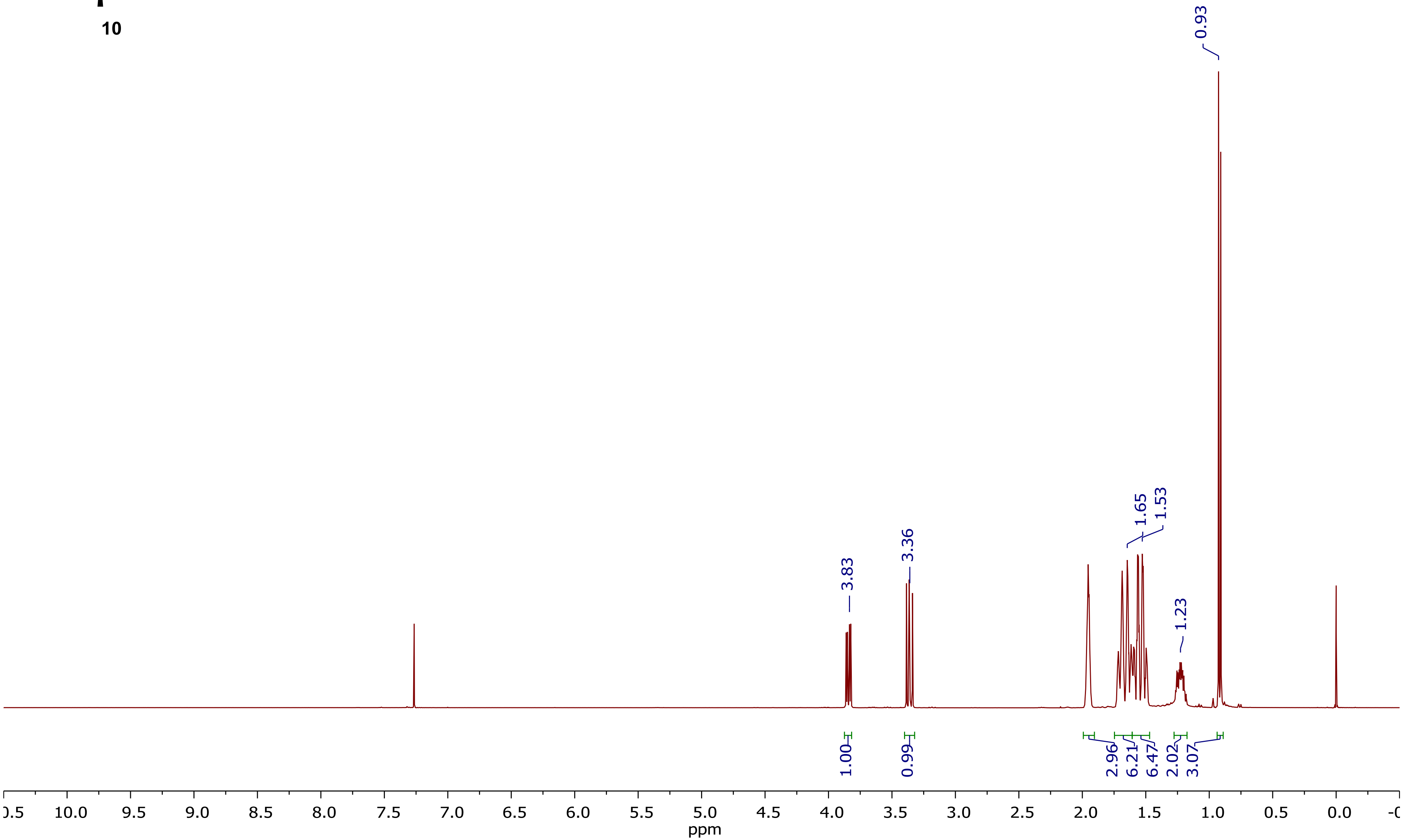

$^{13}C\{^{1}H\}$ NMR (100.6 MHz, $CDCl_3$)

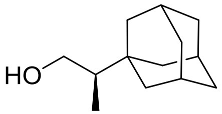

10

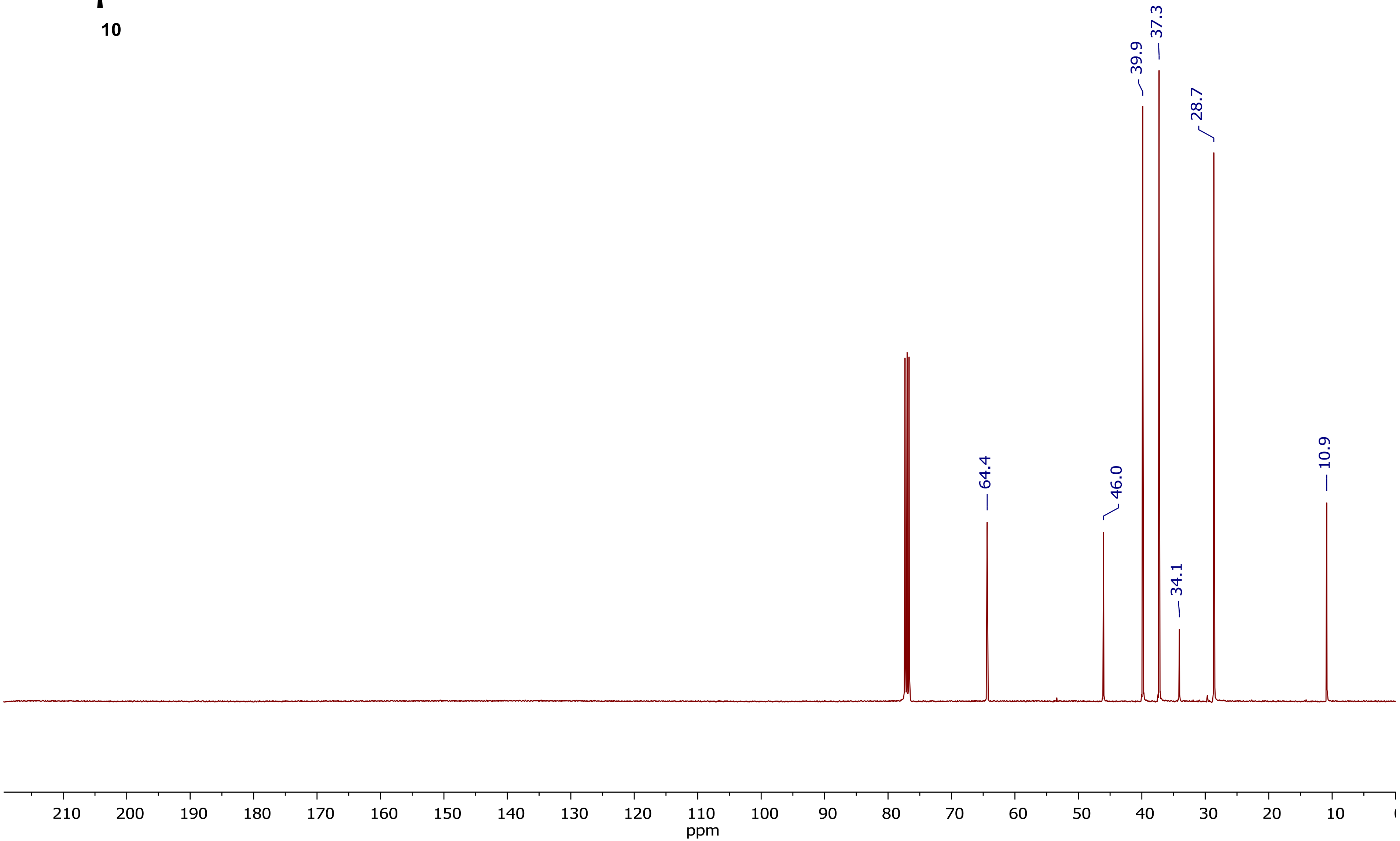

^{1}H NMR (400 MHz, $CDCl_3$)

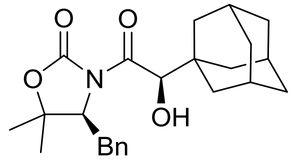

SI3

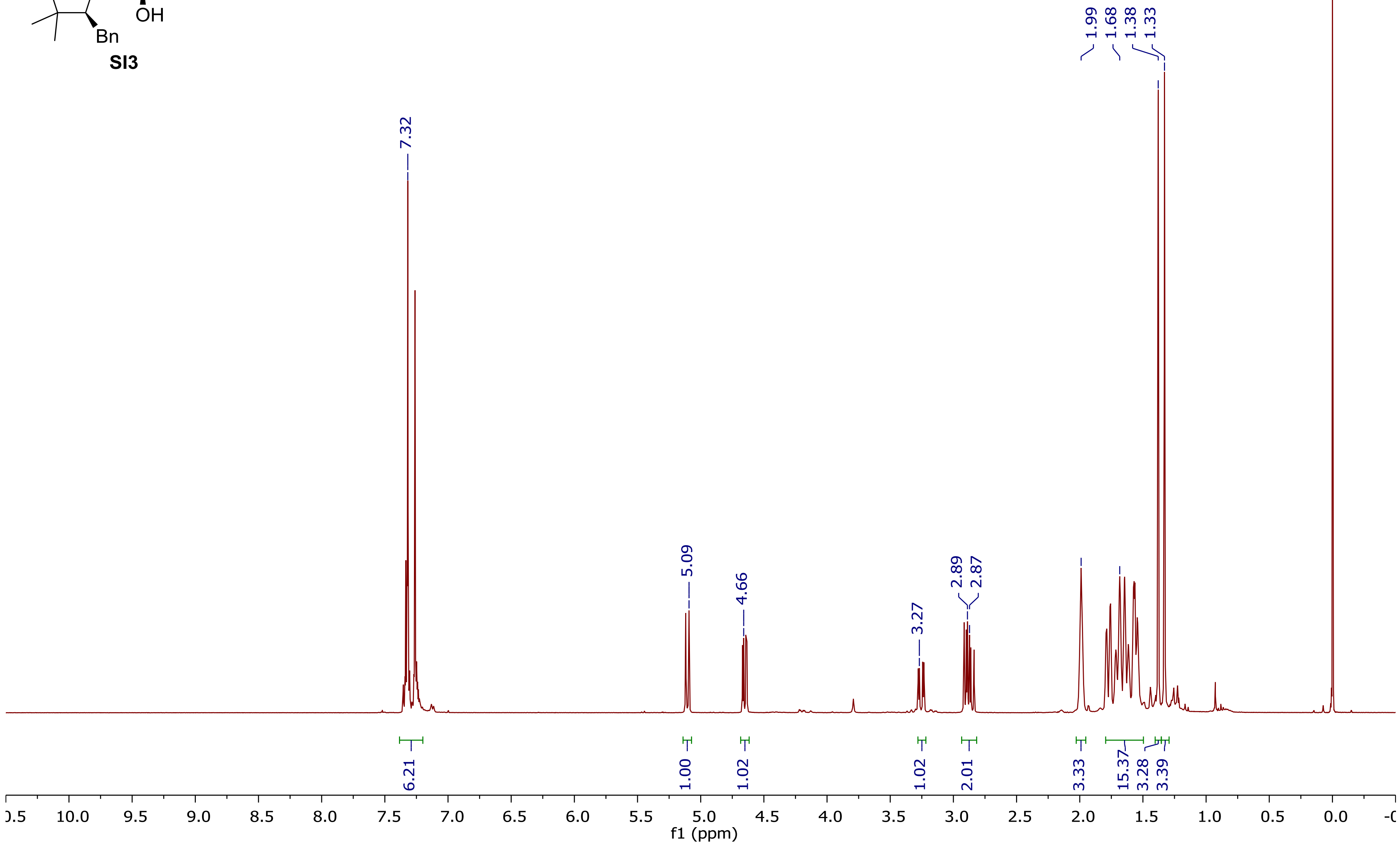

$^{13}C\{^1H\}$ NMR (100.6 MHz, $CDCl_3$)

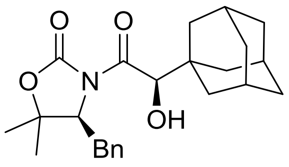

SI3

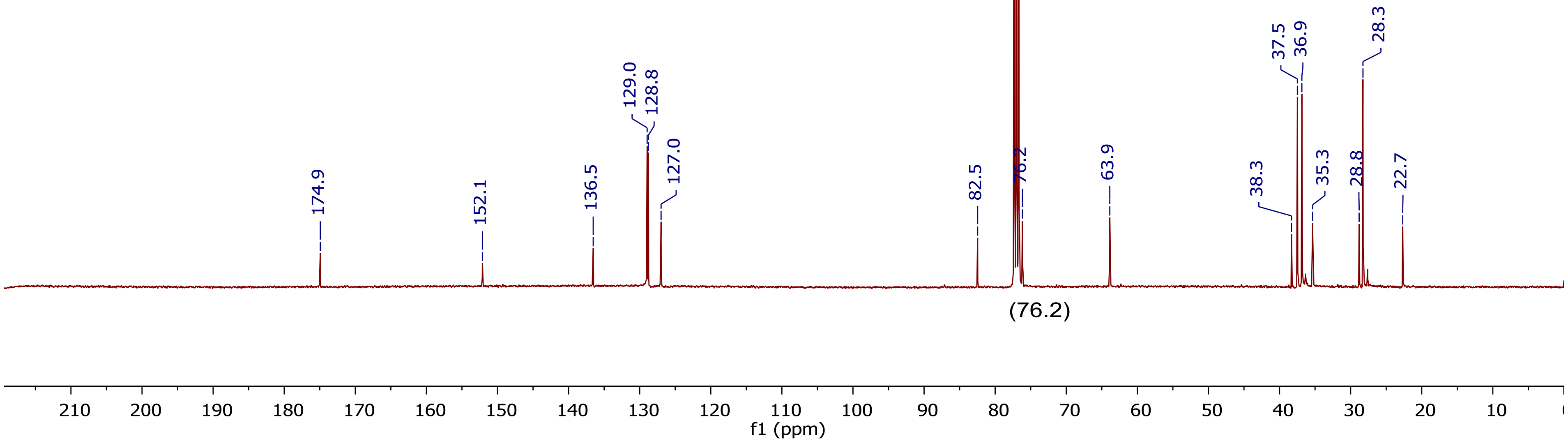

^1H NMR (400 MHz, $CDCl_3$)

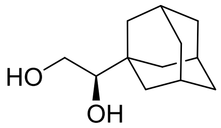

11

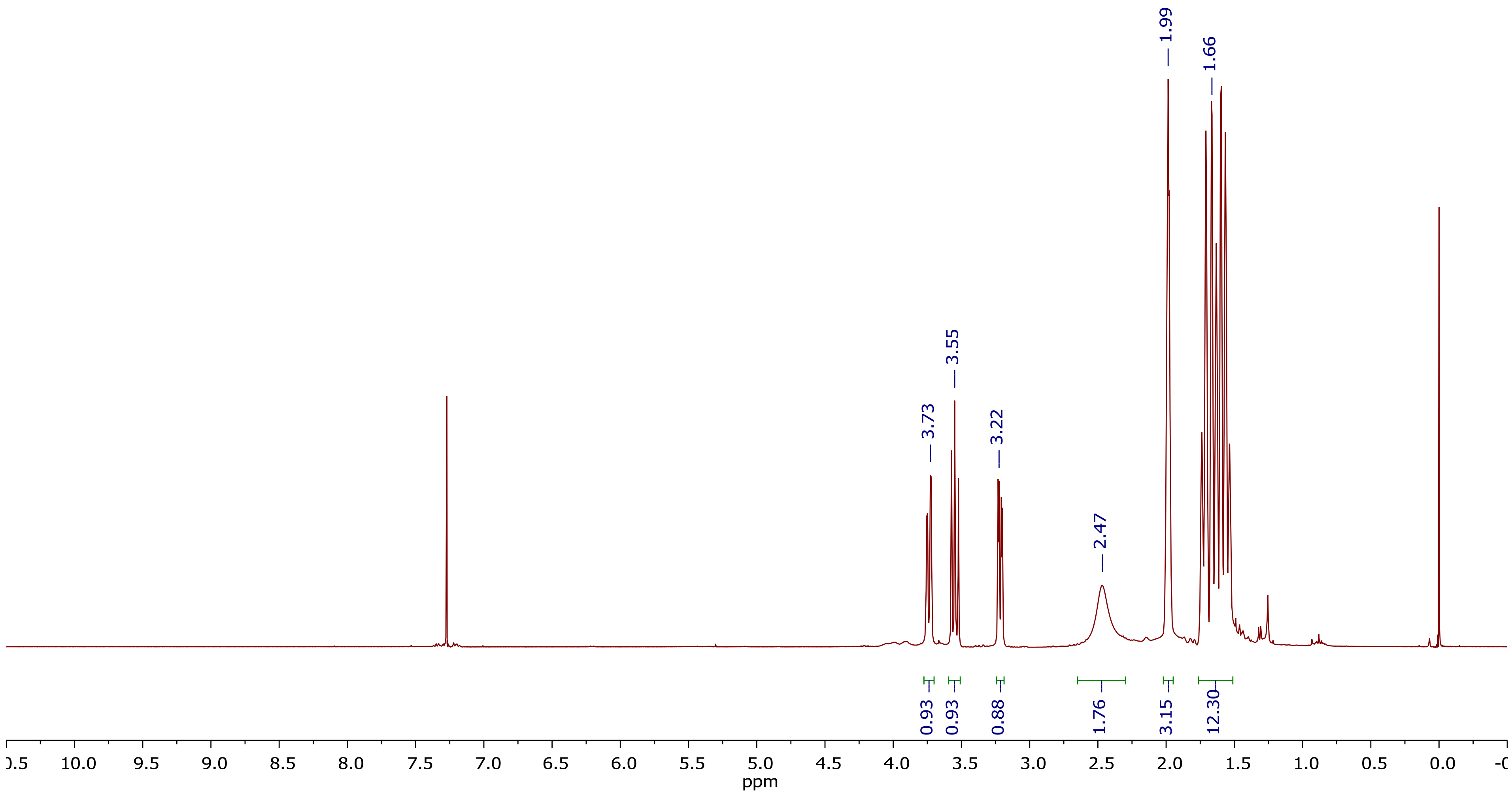

$^{13}C\{^{1}H\}$ NMR (100.6 MHz, $CDCl_3$)

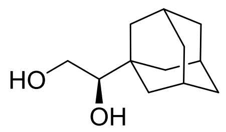

11

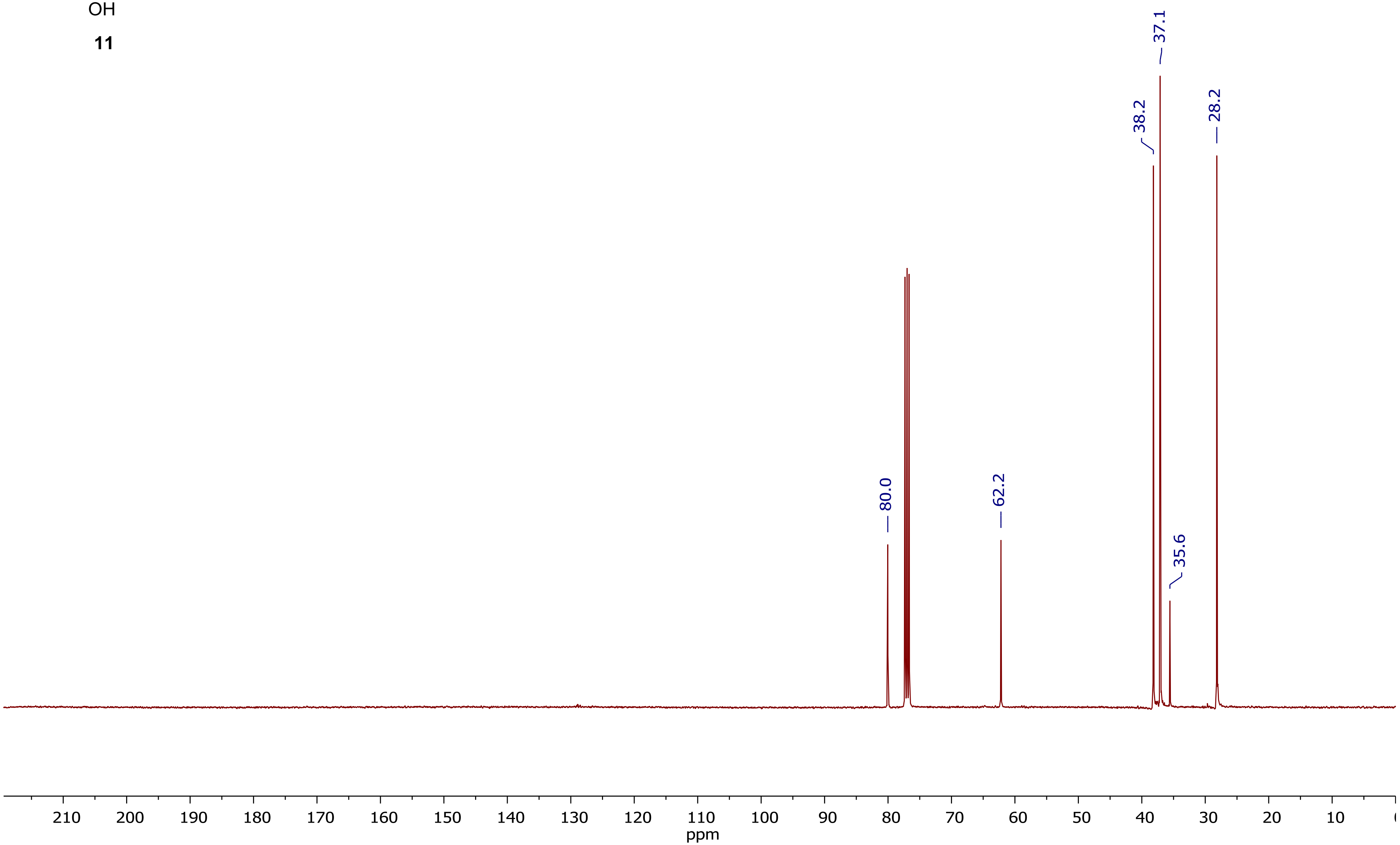

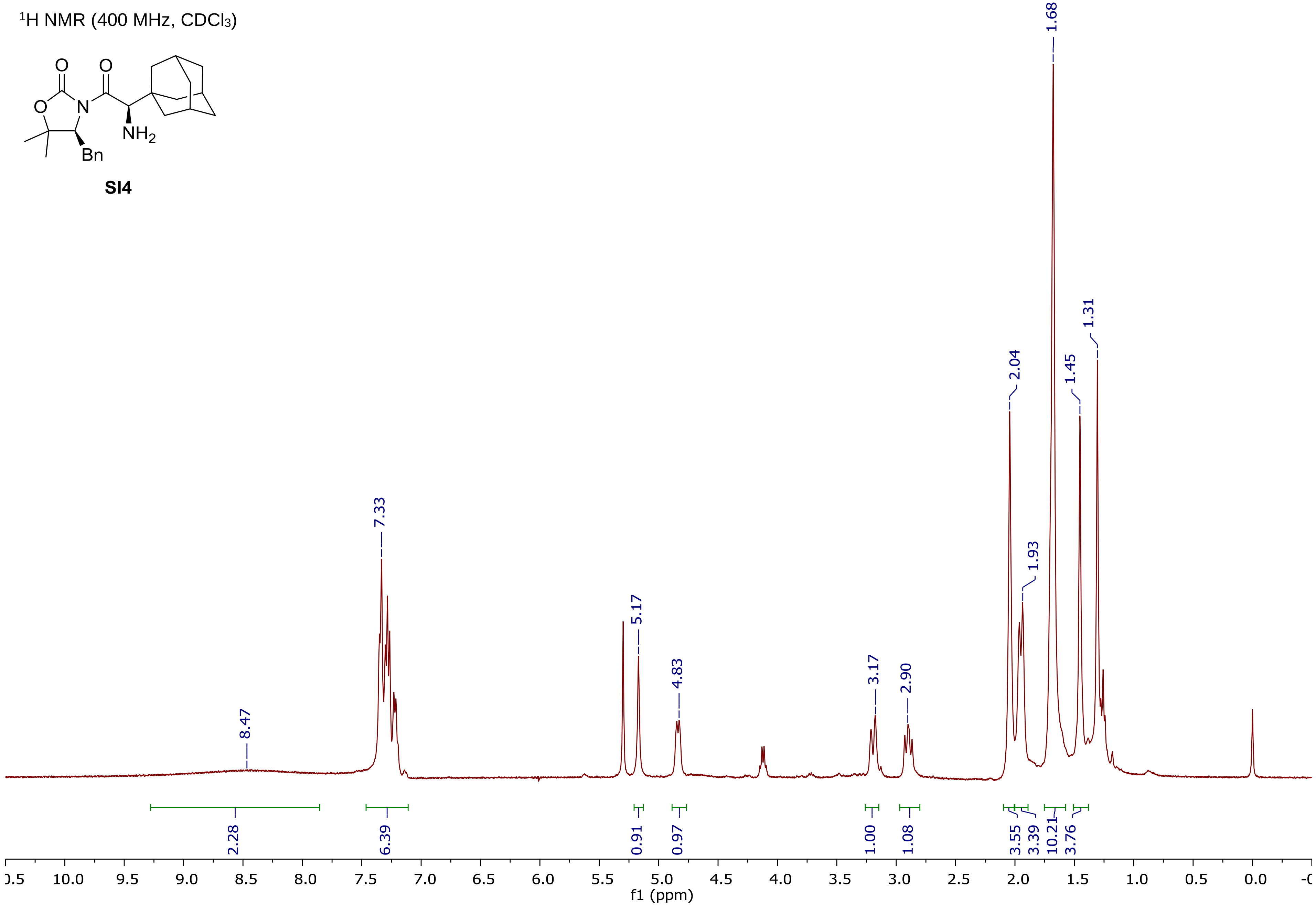
1H NMR (400 MHz, CDCl3)
O
O
O
N
NH2
Bn
SI4
8.47
7.33
5.17
4.83
3.17
2.90
2.04
1.93
1.68
1.45
1.31
2.28
6.39
0.91
0.97
1.00
1.08
3.55
3.39
10.21
3.76
f1 (ppm)

^{1}H NMR (400 MHz, $CDCl_3$)

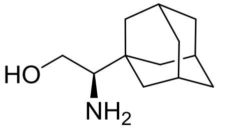

12

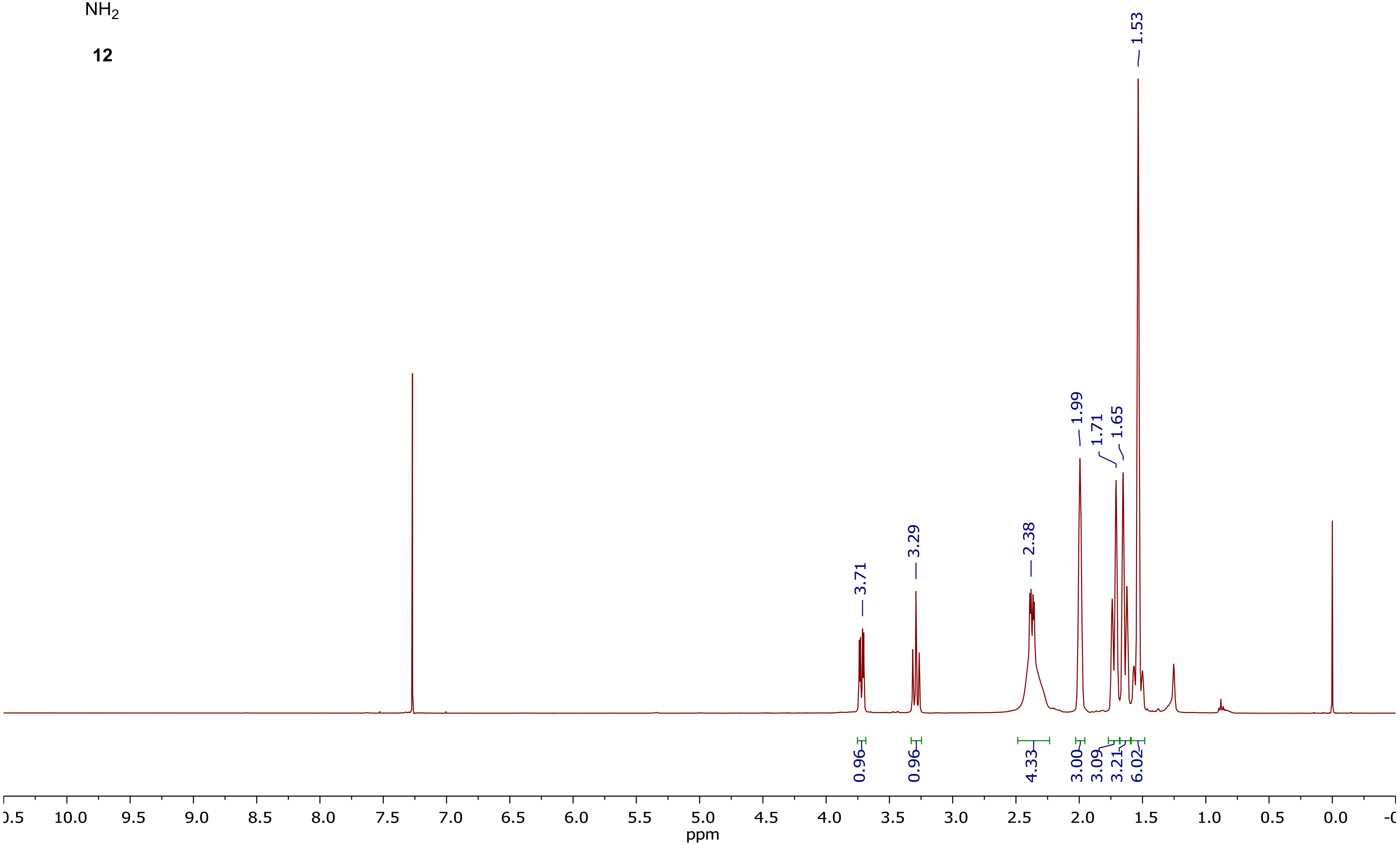

$^{13}C\{^{1}H\}$ NMR (100.6 MHz, $CDCl_3$)

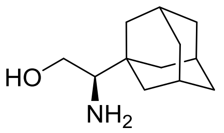

12

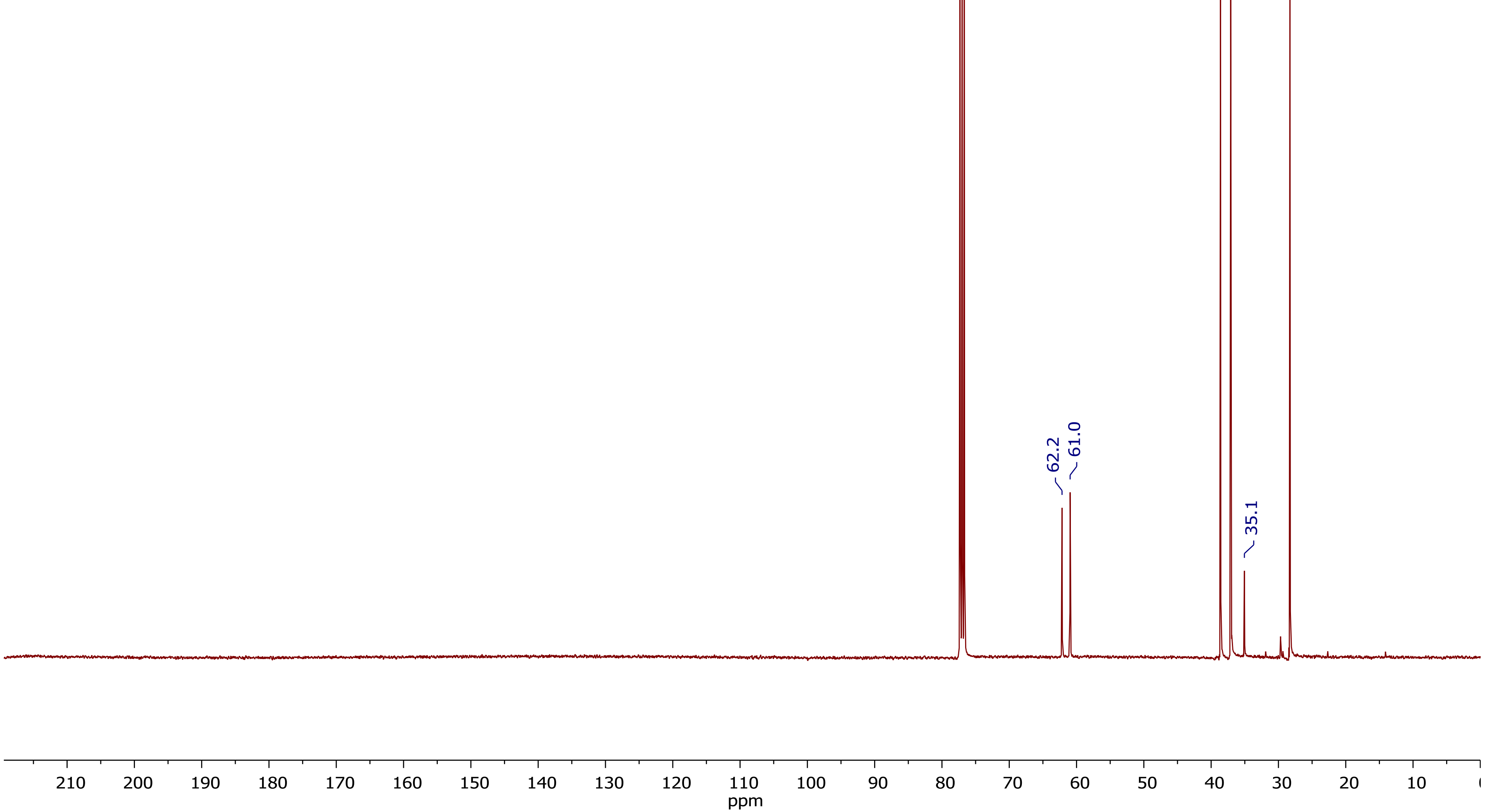

8 References

(1) Bull, S. D.; Davies, S. G.; Jones, S.; Polywka, M. E. C.; Shyam Prasad, R.; Sanganee, H. J. *Synlett* **1998**, *1998*, 519–521.

(2) Gómez-Palomino, A.; Pérez-Palau, M.; Romea, P.; Urpí, F.; Del Olmo, M.; Hesse, T.; Fleckenstein, S.; Gómez-Bengoa, E.; Sotorríos, L.; Font-Bardia, M. *Org. Lett.* **2020**, *22*, 199–203.

(3) Babu, K. R.; Zhu, N.; Bao, H. *Org. Lett.* **2017**, *19*, 46–49.

(4) Shim, E.; Zakarian, A. *Synlett* **2020**, *31*, 683–686.

(5) Pronin, S. V.; Martinez, A.; Kuznedelov, K.; Severinov, K.; Shuman, H. A.; Kozmin, S. A. *J. Am. Chem. Soc.* **2011**, *133*, 12172–12184.

(6) Poh, J. S.; Makai, S.; von Keutz, T.; Tran, D. N.; Battilocchio, C.; Pasau, P.; Ley, S. V. *Angew. Chemie - Int. Ed.* **2017**, *56*, 1864–1868.

(7) Clariana, J.; García-Granda, S.; Gotor, V.; Gutiérrez-Fernández, A.; Luna, A.; Moreno-Maas, M.; Vallribera, A. *Tetrahedron Asymmetry* **2000**, *11*, 4549–4557.
