# Supplementary material for: Stereoselective Alkylation of Chiral Titanium(IV) Enolates with tert-Butyl Peresters
Source: Org Lett. 2021 Oct 26;23(22):8852–6. doi: 10.1021/acs.orglett.1c03366 (PMC8609576; doi:10.1021/acs.orglett.1c03366)
Supplement: Supplementary file 2 — ol1c03366_si_002.pdf [file ol1c03366_si_002.pdf]

Supporting Information

-

Computational Part

Stereoselective Alkylation of Chiral Titanium(IV) Enolates with *tert*-Butyl Peresters

Marina Pérez-Palau,[†] Nil Sanosa,[†,¶] Pedro Romea,[*,†] Fèlix Urpí,[*,†] Rosa López,[&] Enrique Gómez-Bengoa,[*,&] and Mercè Font-Bardia[#]

† Secció de Química Orgànica, Departament de Química Inorgànica i Orgànica and Institut de Biomedicina de la Universitat de Barcelona (IBUB), Universitat de Barcelona, Carrer Martí i Franqués 1-11, 08028 Barcelona, Catalonia, Spain

& Departamento de Química Orgánica I, Universidad del Pais Vasco, UPV/EHU, Apdo. 1072, 20080 San Sebastián, Spain

# Unitat de Difracció de RX. CCiTUB. Universitat de Barcelona. Carrer Solé i Sabarís 1-3, 08028 Barcelona, Catalonia, Spain

Computational details

All structures were optimized using density functional theory (DFT) as implemented in Gaussian 16,[1] with B3LYP[2] as functional, 6-31G(d,p) as basis set for non-metallic atoms, and SDD[3] as basis set for titanium. Final energies were obtained performing single-point calculations on the previously optimized structures at M06-2X[4]/def2tzvpp[5] level of theory, introducing solvation factors with the IEF-PCM[6] method, and dichloroethane as solvent. The stationary points were characterized by frequency calculations in order to verify that they have the right number of imaginary frequencies.

Diastereoselectivity

As mentioned in the manuscript, we have identified a SET mechanism as responsible for the reactivity. The diastereoselectivity would arise from the approach of the perester **a** to the less hindered diastereotopic face of the titanium enolate **I**. Indeed, the two faces present a minimum energy difference of at least 5.0 kcal/mol by positioning both reagents at different C----O distances, ranging from 1.8 to 4.0 Å. The bulkiness of the benzyl directing group in **I** and, specially, the adamantyl group in **a**, are responsible for the good diastereocontrol, and the high energy difference can explain the complete formation of a single diastereomer. For this study, we have not taken into consideration any theoretical aspects regarding the Marcus Inverted Region of the electron transfer process.[7] Thus, we have only considered the regime in which the electron transfer rate decreases with increasing distances. The reason for this is that transfer distances longer than 6.0 Å have a clear negative effect on the computational selectivities and clearly disagree with the experimental results.

---

[1] Gaussian 16, Revision A.03, Frisch, M. J.; Trucks, G. W.; Schlegel, H. B.; Scuseria, G. E.; Robb, M. A.; Cheeseman, J. R.; Scalmani, G.; Barone, V.; Petersson, G. A.; Nakatsuji, H.; Li, X.; Caricato, M.; Marenich, A. V.; Bloino, J.; Janesko, B. G.; Gomperts, R.; Mennucci, B.; Hratchian, H. P.; Ortiz, J. V.; Izmaylov, A. F.; Sonnenberg, J. L.; Williams-Young, D.; Ding, F.; Lipparini, F.; Egidi, F.; Goings, J.; Peng, B.; Petrone, A.; Henderson, T.; Ranasinghe, D.; Zakrzewski, V. G.; Gao, J.; Rega, N.; Zheng, G.; Liang, W.; Hada, M.; Ehara, M.; Toyota, K.; Fukuda, R.; Hasegawa, J.; Ishida, M.; Nakajima, T.; Honda, Y.; Kitao, O.; Nakai, H.; Vreven, T.; Throssell, K.; Montgomery, J. A., Jr.; Peralta, J. E.; Ogliaro, F.; Bearpark, M. J.; Heyd, J. J.; Brothers, E. N.; Kudin, K. N.; Staroverov, V. N.; Keith, T. A.; Kobayashi, R.; Normand, J.; Raghavachari, K.; Rendell, A. P.; Burant, J. C.; Iyengar, S. S.; Tomasi, J.; Cossi, M.; Millam, J. M.; Klene, M.; Adamo, C.; Cammi, R.; Ochterski, J. W.; Martin, R. L.; Morokuma, K.; Farkas, O.; Foresman, J. B.; Fox, D. J. Gaussian, Inc., Wallingford CT, **2016**.

[2] (a) C. Lee, W. Yang and R. G. Parr, *Phys. Rev. B*, **1988**, *37*, 785-789; (b) A. D. Becke, *J. Chem. Phys.*, **1993**, *98*, 5648-5652; (c) W. Kohn, a. D. Becke and R. G. Parr, *J. Phys. Chem*., **1996**, *0*, 12974-12980.

[3] (a) M. Dolg, U. Wedig, H. Stoll, and H. Preuss, *J. Chem. Phys*. 1987, **86**, 866. (b) D. Andrae, U. Haussermann, M. Dolg, H. Stoll and H. Preuss, *Theor. Chim. Acta* 1990, **77**, 123.

[4] Y. Zhao and D. G. Truhlar, *Theor. Chem. Acc.*, **2008**, *120*, 215-241.

[5] (a) F. Furche and R. Ahlrichs, *J. Chem. Phys.* **2003**, *119*, 12753-12762; (b) F. Weigend and R. Ahlrichs, *Phys. Chem. Chem. Phys.* **2005**, *7*, 3297-3305.

[6] (a) E. Cancés, B. Mennucci and J. Tomasi, *J. Chem. Phys.*, **1997**, *107*, 3032-3041; (b) M. Cossi, V. Barone, B. Mennucci and J. Tomasi, *Chem. Phys. Lett.*, **1998**, *286*, 253-260; (c) J. Tomasi, B. Mennucci and E. Cancès, *J. Mol.Struc.: THEOCHEM*, **1999**, *464*, 211-226.

[7] Kuss-Petermann, M.; Wenger, O. S. *Phys. Chem. Chem. Phys.* **2016**, *18*, 18657-18664.

In Figure S1, the 3-D representations of the **pro-S** and **pro-R** approaches is shown, maintaining an electron transfer distance of 1.8 Å. The energy difference between both structures is 7.6 kcal/mol, accounting for a diastereoselectivity of > 99:1.

Figure S1. 3D- representation of the approach of the perester to both diastereotopic faces of the Ti-enolate

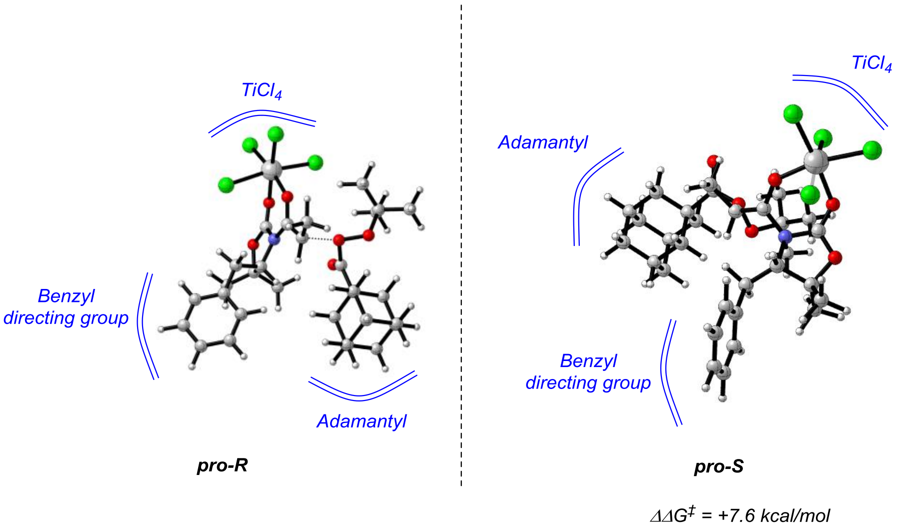

Cartesian coordinates of the optimized structures are shown below, as well as their single point energy and correction to Gibbs free energy (in Hartrees).

Radical **IV**

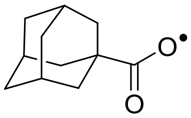

E = -578.595910

G_{corr} = 0.210204

Standard orientation:

Center Number	Atomic Number	Atomic Type	Coordinates (Angstroms) X	Y	Z
1	6	0	-0.765229	0.025418	-0.000190
2	6	0	-0.248757	-0.720597	-1.264110
3	6	0	-0.229559	1.474797	-0.000885
4	6	0	-0.249038	-0.719253	1.264653
5	1	0	-0.621807	-0.219179	-2.165994
6	1	0	-0.644567	-1.742320	-1.275819
7	6	0	1.293073	-0.733192	-1.259687
8	1	0	-0.603959	2.011457	0.878845
9	1	0	-0.603752	2.010530	-0.881268
10	6	0	1.313927	1.451835	-0.000699
11	1	0	-0.644865	-1.740961	1.277357
12	1	0	-0.622285	-0.216862	2.165914
13	6	0	1.292791	-0.731867	1.260581
14	1	0	1.646075	-1.255666	-2.157253
15	6	0	1.818927	0.717326	-1.260269
16	6	0	1.798242	-1.464779	0.000888
17	1	0	1.685333	2.483735	-0.001196
18	6	0	1.818649	0.718648	1.259752
19	1	0	1.645588	-1.253392	2.158779
20	1	0	1.481638	1.241254	-2.163913
21	1	0	2.916103	0.718939	-1.281672
22	1	0	1.446488	-2.504475	0.001395
23	1	0	2.895057	-1.498797	0.001028
24	1	0	2.915821	0.720286	1.281390
25	1	0	1.481164	1.243529	2.162770
26	6	0	-2.270383	0.006596	-0.000332
27	8	0	-3.009657	1.031461	0.000090
28	8	0	-2.966328	-1.049919	0.000087

Anion **III**

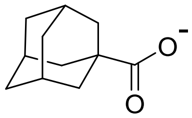

E = -578.805127

G_{corr} = 0.208763

Standard orientation:

Center Number	Atomic Number	Atomic Type	X	Y	Z
1	6	0	-0.793229	0.044025	-0.000073
2	6	0	-0.264248	-0.698064	-1.249002
3	6	0	-0.225237	1.475869	-0.000969
4	6	0	-0.264344	-0.696476	1.249835
5	1	0	-0.627872	-0.193749	-2.156303
6	1	0	-0.691956	-1.706669	-1.253577
7	6	0	1.278165	-0.737431	-1.259911
8	1	0	-0.605032	2.017598	0.873136
9	1	0	-0.604985	2.016489	-0.875783
10	6	0	1.320126	1.448606	-0.000910
11	1	0	-0.692041	-1.705072	1.255655
12	1	0	-0.628045	-0.191014	2.156465
13	6	0	1.278071	-0.735802	1.260897
14	1	0	1.638558	-1.263779	-2.157397
15	6	0	1.825310	0.706240	-1.257062
16	6	0	1.780877	-1.471723	0.000987
17	1	0	1.716683	2.475698	-0.001558
18	6	0	1.825224	0.707862	1.256230
19	1	0	1.638400	-1.260993	2.159086
20	1	0	1.490636	1.233415	-2.161403
21	1	0	2.926345	0.698334	-1.279525
22	1	0	1.411982	-2.506511	0.001640
23	1	0	2.881023	-1.521806	0.001057
24	1	0	2.926257	0.699986	1.278778
25	1	0	1.490486	1.236202	2.159867
26	6	0	-2.377564	0.005805	-0.000171
27	8	0	-2.961291	1.116787	0.000058
28	8	0	-2.859877	-1.156987	0.000037

tert-Butoxide anion

E = -233.130204

G_{corr} = 0.090961

Standard orientation:

Center Number	Atomic Number	Atomic Type	X	Y	Z
1	8	0	-0.000033	-0.001106	1.483559
2	6	0	0.000012	-0.000228	0.159548
3	6	0	-1.370604	-0.512426	-0.437271
4	1	0	-1.557346	-1.525919	-0.057669
5	1	0	-2.176453	0.129395	-0.056854
6	1	0	-1.436880	-0.536640	-1.540098
7	6	0	1.129418	-0.930149	-0.437459
8	1	0	1.183875	-0.974771	-1.540308
9	1	0	2.100434	-0.585345	-0.057545
10	1	0	0.976448	-1.949157	-0.057561
11	6	0	0.241201	1.443674	-0.436124
12	1	0	0.252589	1.513842	-1.538914
13	1	0	-0.542865	2.111933	-0.055601
14	1	0	1.200289	1.820288	-0.056089

tert-Butoxide radical

E = -232.983364

G_{corr} = 0.092930

Standard orientation:

Center Number	Atomic Number	Atomic Type	X	Y	Z
1	8	0	0.000242	0.264468	1.431253
2	6	0	-0.000022	-0.026196	0.080920
3	6	0	-1.277898	-0.792518	-0.314336
4	1	0	-1.305766	-1.762510	0.191504
5	1	0	-2.165022	-0.226680	-0.017518
6	1	0	-1.315451	-0.970186	-1.394155
7	6	0	1.276959	-0.793957	-0.314441
8	1	0	1.314251	-0.971602	-1.394273
9	1	0	2.164753	-0.229154	-0.017650
10	1	0	1.303736	-1.764003	0.191354
11	6	0	0.000753	1.388087	-0.580710
12	1	0	0.000637	1.259722	-1.667389
13	1	0	-0.888787	1.948590	-0.285200
14	1	0	0.890962	1.947583	-0.285289

TS-decarb

E = -578.597118

G_{corr} = 0.206383

Imaginary frequency = -1076.7 cm^{-1}

Standard orientation:

Center Number	Atomic Number	Atomic Type	X	Y	Z
1	6	0	-0.771926	0.059811	-0.000016
2	6	0	-0.198773	1.496732	-0.000200
3	6	0	-0.283303	-0.691064	1.266476
4	6	0	-0.283315	-0.691424	-1.266309
5	1	0	-0.551985	2.044851	0.881036
6	1	0	-0.551956	2.044641	-0.881585
7	6	0	1.344339	1.432064	-0.000191
8	1	0	-0.712906	-1.697353	1.277614
9	1	0	-0.636577	-0.173115	2.166937
10	6	0	1.261389	-0.747625	1.259424
11	1	0	-0.636561	-0.173724	-2.166932
12	1	0	-0.712941	-1.697703	-1.277162
13	6	0	1.261379	-0.747982	-1.259225
14	1	0	1.741593	2.454328	-0.000353
15	6	0	1.829811	0.685864	1.259480
16	6	0	1.829811	0.685503	-1.259651
17	1	0	1.594054	-1.280385	2.159011
18	6	0	1.741156	-1.496321	0.000200
19	1	0	1.594057	-1.280959	-2.158680
20	1	0	1.508236	1.219891	2.162965
21	1	0	2.926590	0.655706	1.280444
22	1	0	1.508261	1.219297	-2.163283
23	1	0	2.926589	0.655323	-1.280594

24	1	0	2.836583	-1.565956	0.000200
25	1	0	1.355312	-2.523538	0.000351
26	6	0	-2.323859	-0.035701	-0.000007
27	8	0	-2.941996	-1.088576	0.000050
28	8	0	-2.886578	1.138519	-0.000033

Adamantyl radical **V**

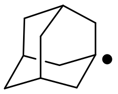

E = -390.020357

G_{corr} = 0.198840

Standard orientation:

Center Number	Atomic Number	Atomic Type	Coordinates (Angstroms) X	Y	Z
1	6	0	0.000036	0.001764	1.488093
2	6	0	1.435012	-0.212422	1.081377
3	6	0	-0.902604	-1.134575	1.082386
4	6	0	-0.532383	1.350931	1.079702
5	1	0	1.826064	-1.165979	1.458734
6	1	0	2.087204	0.586647	1.456537
7	6	0	1.447117	-0.216214	-0.480751
8	1	0	-1.924339	-0.995790	1.458399
9	1	0	-0.537101	-2.098229	1.459674
10	6	0	-0.910218	-1.145935	-0.479703
11	1	0	0.098270	2.167079	1.454932
12	1	0	-1.550102	1.516858	1.455602
13	6	0	-0.536779	1.360286	-0.482375
14	1	0	2.471201	-0.369231	-0.845919
15	6	0	0.534445	-1.356205	-0.985121
16	6	0	0.906258	1.139202	-0.987837
17	1	0	-1.554453	-1.956921	-0.844093
18	6	0	-1.440820	0.213515	-0.986776
19	1	0	-0.916640	2.323034	-0.848880
20	1	0	0.916948	-2.325645	-0.639657
21	1	0	0.544979	-1.384475	-2.082639
22	1	0	1.554892	1.955751	-0.644404
23	1	0	0.924225	1.160685	-2.085429
24	1	0	-1.469601	0.216552	-2.084330
25	1	0	-2.471932	0.367582	-0.642492

Adamantyl anion

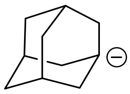

E = -390.088041

G_{corr} = 0.193992

Standard orientation:

Center Number	Atomic Number	Atomic Type	Coordinates (Angstroms) X	Y	Z
1	6	0	-0.001949	-0.002587	1.640197
2	6	0	-1.385568	-0.319717	1.072219
3	6	0	0.415485	1.356125	1.076692
4	6	0	0.966830	-1.041026	1.073766
5	1	0	-2.140897	0.413532	1.419051
6	1	0	-1.744827	-1.310024	1.416725
7	6	0	-1.422119	-0.326060	-0.491037
8	1	0	1.428044	1.642730	1.424603
9	1	0	-0.262735	2.161282	1.423369
10	6	0	0.428801	1.396026	-0.486527
11	1	0	0.709381	-2.062320	1.418902
12	1	0	2.003064	-0.856492	1.421097
13	6	0	0.994831	-1.067791	-0.489511
14	1	0	-2.433083	-0.557908	-0.881874
15	6	0	-0.988452	1.065193	-1.000764
16	6	0	-0.425222	-1.387540	-1.003351
17	1	0	0.733640	2.388664	-0.874242
18	6	0	1.416844	0.326708	-0.999660
19	1	0	1.702165	-1.826941	-0.879333
20	1	0	-1.699040	1.828029	-0.641549
21	1	0	-1.019250	1.101075	-2.103971
22	1	0	-0.731357	-2.384627	-0.645596
23	1	0	-0.437955	-1.431418	-2.106608
24	1	0	1.463179	0.338754	-2.102866
25	1	0	2.432790	0.559674	-0.639863

Pro-R approach

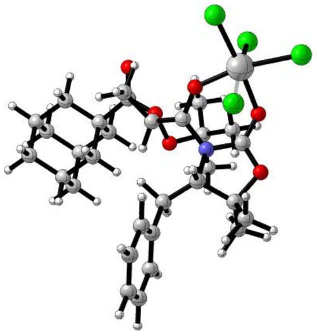

E = -4365.1446134

G_{corr} = 0.610787

Standard orientation:

Center Number	Atomic Number	Atomic Type	Coordinates (Angstroms) X	Y	Z
1	6	0	-0.960867	-0.029114	-0.601592
2	8	0	-2.090801	-0.665342	-0.789669
3	22	0	-3.889697	-0.618991	-0.125184
4	6	0	0.194581	-0.352978	-1.273575
5	1	0	1.026608	0.333179	-1.130979
6	6	0	0.078422	-0.937092	-2.664922
7	1	0	0.906762	-1.617563	-2.886814

8	1	0	0.079995	-0.137857	-3.415279
9	1	0	-0.860329	-1.483542	-2.766555
10	6	0	-0.123059	2.121693	0.556189
11	17	0	-4.014080	1.605996	-1.106867
12	17	0	-4.703713	-1.668302	-1.993491
13	17	0	-3.520819	-2.613439	1.073896
14	7	0	-0.966482	0.918466	0.475693
15	6	0	-1.990640	1.012354	1.353424
16	8	0	-3.013527	0.315378	1.411852
17	6	0	-0.484460	2.611956	1.997651
18	6	0	-0.653476	4.112592	2.191631
19	1	0	0.290421	4.634818	2.018370
20	1	0	-0.975508	4.304623	3.219256
21	1	0	-1.410159	4.518837	1.518703
22	6	0	0.466879	2.014251	3.037266
23	1	0	1.451892	2.487061	2.956696
24	1	0	0.586228	0.938867	2.888110
25	1	0	0.076730	2.194457	4.043031
26	8	0	-1.806060	2.018727	2.218136
27	6	0	-0.426882	3.062460	-0.634500
28	1	0	-1.446481	3.447091	-0.543321
29	1	0	-0.440903	2.420025	-1.522645
30	6	0	0.565975	4.185393	-0.850102
31	6	0	1.931897	3.921946	-1.041760
32	6	0	0.141070	5.520329	-0.914550
33	6	0	2.841848	4.954546	-1.271042
34	1	0	2.288207	2.894730	-1.029752
35	6	0	1.046076	6.556759	-1.148785
36	1	0	-0.914153	5.746007	-0.784944
37	6	0	2.402804	6.278945	-1.322874
38	1	0	3.893719	4.722853	-1.416886
39	1	0	0.688835	7.582064	-1.196233
40	1	0	3.109530	7.084256	-1.503383
41	1	0	0.931385	1.833850	0.541179
42	17	0	-5.920900	-0.260004	0.924293
43	8	0	0.973770	-1.796113	-0.524550
44	8	0	1.514945	-3.128952	-0.624712
45	6	0	1.785231	-1.275083	0.540751
46	8	0	1.279988	-0.945930	1.581109
47	6	0	0.538627	-4.146012	-0.233683
48	6	0	0.145989	-4.008446	1.239228
49	1	0	-0.407880	-3.085473	1.417007
50	1	0	-0.507590	-4.838599	1.524382
51	1	0	1.031814	-4.029492	1.883690
52	6	0	-0.687990	-4.082590	-1.146487
53	1	0	-1.256444	-3.164807	-0.988007
54	1	0	-0.386521	-4.146590	-2.196986
55	1	0	-1.359745	-4.918023	-0.926041
56	6	0	1.337480	-5.434912	-0.475895
57	1	0	1.669770	-5.493419	-1.516779
58	1	0	2.216577	-5.484268	0.175068
59	1	0	0.705237	-6.303551	-0.265468
60	6	0	3.295447	-1.163326	0.277686
61	6	0	3.776169	0.201018	0.851230
62	6	0	4.022557	-2.294376	1.066487
63	6	0	5.303454	0.342133	0.699409
64	1	0	3.481979	0.275162	1.902779
65	1	0	3.275922	1.022357	0.320815
66	6	0	5.549905	-2.151275	0.915559
67	1	0	3.732906	-2.237654	2.122678
68	1	0	3.688772	-3.262941	0.684747
69	6	0	6.003774	-0.789714	1.478133
70	1	0	5.612462	1.314900	1.103911
71	1	0	6.038346	-2.962418	1.471358
72	1	0	7.094107	-0.687984	1.392615
73	1	0	5.757941	-0.721114	2.545981
74	6	0	5.927480	-2.243159	-0.577399
75	1	0	7.016897	-2.167916	-0.696828
76	6	0	5.684634	0.252745	-0.792151
77	1	0	6.770171	0.369892	-0.912282
78	6	0	3.697333	-1.248345	-1.213586
79	1	0	3.360184	-2.199024	-1.632411
80	1	0	3.201117	-0.452230	-1.782090
81	6	0	5.228759	-1.108403	-1.355033
82	1	0	5.493234	-1.174689	-2.418142
83	1	0	5.626334	-3.217729	-0.982484
84	1	0	5.211468	1.070224	-1.352580

Pro-S approach

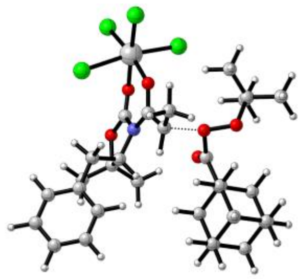

E = -4365.135522

G_{corr} = 0.613868

Standard orientation:

Center Number	Atomic Number	Atomic Type	Coordinates (Angstroms) X	Y	Z
1	6	0	0.844530	-0.284970	-0.555115
2	8	0	1.629152	-1.242300	-1.049912
3	22	0	3.225843	-2.094859	-0.570127
4	6	0	-0.484916	-0.203321	-0.868551
5	1	0	-0.996644	0.683606	-0.519852
6	6	0	-0.873373	-0.765297	-2.227742
7	1	0	-1.930401	-0.601712	-2.461187
8	1	0	-0.286350	-0.276068	-3.009951
9	1	0	-0.663390	-1.835107	-2.288953
10	6	0	1.744673	2.027748	0.216731
11	17	0	4.362667	0.002229	-1.318471
12	17	0	3.496357	-3.101294	-2.609837
13	17	0	2.044294	-3.903974	0.388044
14	7	0	1.476366	0.577262	0.422795
15	6	0	2.536464	0.088358	1.113272
16	8	0	2.923979	-1.091918	1.134949
17	6	0	2.586628	2.344027	1.490108
18	6	0	3.752764	3.287209	1.218344
19	1	0	3.384066	4.273741	0.923982
20	1	0	4.365361	3.395817	2.118553
21	1	0	4.377787	2.880654	0.419751
22	6	0	1.770075	2.757845	2.714804
23	1	0	1.379069	3.772304	2.599008
24	1	0	0.933796	2.073271	2.884014
25	1	0	2.414893	2.735776	3.597809
26	8	0	3.176859	1.036056	1.800922
27	17	0	5.235149	-2.807895	0.378339
28	8	0	-1.655254	-1.199568	0.071609

29	8	0	-1.939810	-0.639612	1.369032
30	6	0	-2.888544	-1.766890	-0.440005
31	8	0	-2.816069	-2.858945	-0.937207
32	6	0	0.489346	2.875014	-0.027788
33	1	0	-0.018852	2.424395	-0.886033
34	1	0	-0.195444	2.798175	0.822669
35	6	0	0.769502	4.330868	-0.349005
36	6	0	0.266431	5.359689	0.458483
37	6	0	1.509575	4.684479	-1.489088
38	6	0	0.501983	6.701428	0.148530
39	1	0	-0.324234	5.106709	1.335753
40	6	0	1.749863	6.022541	-1.799571
41	1	0	1.899535	3.905515	-2.139010
42	6	0	1.248254	7.037670	-0.980758
43	1	0	0.100496	7.481369	0.790201
44	1	0	2.327737	6.272273	-2.685119
45	1	0	1.435380	8.079909	-1.224023
46	1	0	2.403116	2.104063	-0.658149
47	6	0	-1.509313	-1.534575	2.467993
48	6	0	0.009063	-1.582101	2.560603
49	1	0	0.305576	-2.167727	3.436912
50	1	0	0.455578	-2.061694	1.687918
51	1	0	0.414161	-0.573941	2.669563
52	6	0	-2.115082	-0.790134	3.666554
53	1	0	-1.757625	0.243417	3.700818
54	1	0	-3.208612	-0.781718	3.628273
55	1	0	-1.806166	-1.288255	4.590091
56	6	0	-2.093954	-2.940035	2.321865
57	1	0	-3.185923	-2.924143	2.257825
58	1	0	-1.688252	-3.444561	1.443166
59	1	0	-1.816272	-3.528951	3.201571
60	6	0	-4.159299	-0.899620	-0.461938
61	6	0	-4.949507	-1.281421	-1.747936
62	6	0	-5.050692	-1.249208	0.767282
63	6	0	-6.281432	-0.509789	-1.814607
64	1	0	-5.125055	-2.361138	-1.756332
65	1	0	-4.339197	-1.057097	-2.631203
66	6	0	-6.385262	-0.479335	0.696851
67	1	0	-5.235212	-2.330414	0.785150
68	1	0	-4.519633	-0.989317	1.686824
69	6	0	-7.144408	-0.865856	-0.587576
70	1	0	-6.810579	-0.794830	-2.733131
71	1	0	-6.988690	-0.738973	1.576577
72	1	0	-8.105394	-0.335860	-0.632499
73	1	0	-7.370349	-1.940217	-0.583996
74	6	0	-3.899884	0.627333	-0.474770
75	1	0	-3.345245	0.921444	0.419333
76	1	0	-3.286126	0.893582	-1.343055
77	6	0	-6.001454	1.006296	-1.824394
78	1	0	-5.408740	1.274913	-2.708539
79	1	0	-6.944293	1.566148	-1.888712
80	6	0	-6.100250	1.036448	0.690657
81	1	0	-7.044024	1.597715	0.667021
82	1	0	-5.578117	1.326024	1.611992
83	6	0	-5.240842	1.391343	-0.539970
84	1	0	-5.031254	2.468671	-0.543788
